# Single Cell RNA-Sequencing of Pluripotent States Unlocks Modular Transcriptional Variation

**DOI:** 10.1016/j.stem.2015.09.011

**Published:** 2015-10-01

**Authors:** Aleksandra A. Kolodziejczyk, Jong Kyoung Kim, Jason C.H. Tsang, Tomislav Ilicic, Johan Henriksson, Kedar N. Natarajan, Alex C. Tuck, Xuefei Gao, Marc Bühler, Pentao Liu, John C. Marioni, Sarah A. Teichmann

**Affiliations:** 1 European Molecular Biology Laboratory, European Bioinformatics Institute (EMBL-EBI), Wellcome Trust Genome Campus, Hinxton, Cambridge CB10 1SD, UK; 2 Wellcome Trust Sanger Institute, Wellcome Trust Genome Campus, Hinxton, Cambridge CB10 1SA, UK; 3 Friedrich Miescher Institute for Biomedical Research, Maulbeerstrasse 66, 4058 Basel, Switzerland; 4 University of Cambridge, Cancer Research UK Cambridge Institute, Robinson Way, Cambridge, CB2 0RE, UK

## Abstract

Embryonic stem cell (ESC) culture conditions are important for maintaining long-term self-renewal, and they influence cellular pluripotency state. Here, we report single cell RNA-sequencing of mESCs cultured in three different conditions: serum, 2i, and the alternative ground state a2i. We find that the cellular transcriptomes of cells grown in these conditions are distinct, with 2i being the most similar to blastocyst cells and including a subpopulation resembling the two-cell embryo state. Overall levels of intercellular gene expression heterogeneity are comparable across the three conditions. However, this masks variable expression of pluripotency genes in serum cells and homogeneous expression in 2i and a2i cells. Additionally, genes related to the cell cycle are more variably expressed in the 2i and a2i conditions. Mining of our dataset for correlations in gene expression allowed us to identify additional components of the pluripotency network, including Ptma and Zfp640, illustrating its value as a resource for future discovery.

## Highlights

• mESCs grown in three different conditions show distinct transcriptomes
• Global intercellular variation is at similar levels in all three conditions
• 2i and a2i conditions maintain pluripotency with metabolic variation
• Correlation analysis identifies additional pluripotency network genes

## Graphical Abstract

**Figure:**
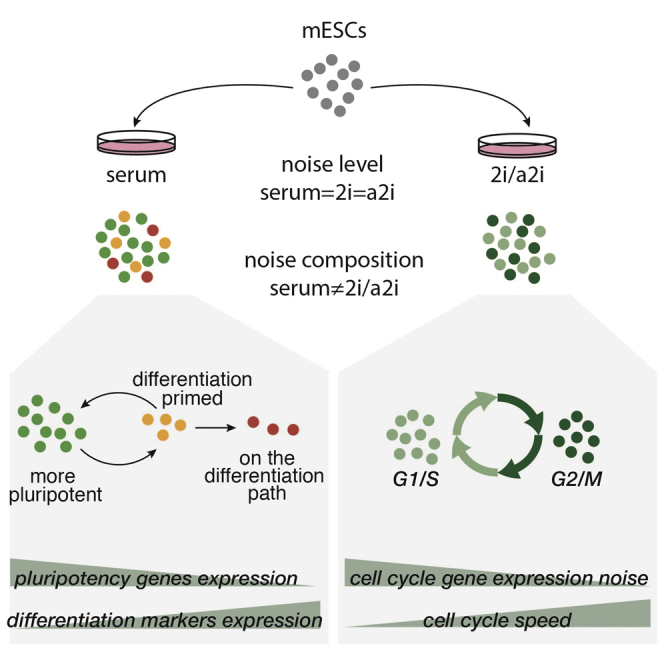

## Teaser

Teichmann, Marioni, and colleagues report full-transcript single cell RNA-sequencing of mESCs cultured in three different conditions: serum, 2i, and the alternative ground state a2i. They find that overall levels of intercellular heterogeneity are comparable across the three conditions, but different sets of genes are variably expressed.

## Introduction

Mouse embryonic stem cells (mESCs) are derived ex vivo from the inner cell mass of the developing blastocyst. They are characterized by their capacity for in vitro self-renewal and the preservation of developmental pluripotency to reconstitute embryonic lineages (Bradley et al., 1984; Evans and Kaufman, 1981; Martin, 1981). Genetic studies have established the role of *Oct4* (Nichols et al., 1998), *Sox2* (Avilion et al., 2003), *Nanog* (Chambers et al., 2003; Mitsui et al., 2003) and *Esrrb* (Festuccia et al., 2012) as the signature core factors in the pluripotency transcriptional network of mESCs (Chen et al., 2008; Loh et al., 2006; Marson et al., 2008).

Maintenance of self-renewal in vitro is dependent on the interplay between extracellular cues and the pluripotency network. This is conventionally achieved through combinatorial stimulation of the JAK-STAT pathway and ID proteins by cytokine leukemia inhibitory factor (LIF) and fetal calf serum (serum)/bone morphogenetic proteins (BMPs), respectively (Smith et al., 1988; Williams et al., 1988; Ying et al., 2003). mESCs propagated in serum/LIF conditions remain exposed to differentiation cues from autocrine fibroblast growth factor 4 (FGF4) or LIF through the RAS-ERK signaling pathway (Burdon et al., 1999; Kunath et al., 2007; Niwa et al., 2009; Ying et al., 2008), although genetic and chemical inhibition of the FGF-ERK pathway alone is able to prevent differentiation (Kunath et al., 2007). These findings led to the establishment of the concept of “ground state pluripotency,” where differentiation cues are shielded, and the pluripotency network is intrinsically stable (Nichols and Smith, 2009; Ying et al., 2008).

With additional inhibition of glycogen synthase kinase 3 (GSK3), ground state mESCs can be robustly maintained in vitro in the chemically defined 2i condition. Dual inhibition of GSK3 and ERK promotes self-renewal by alleviating TCF3-mediated repression, activating *Esrrb* expression, reducing degradation of KLF2 (Martello et al., 2012; Wray et al., 2011; Yeo et al., 2014), and inducing *Tfcp2l1* in concert with LIF (Ye et al., 2013). Substituting ERK kinase inhibition with inhibition of members of the SRC tyrosine kinase family can enable maintenance of an alternative ground state, alternative 2i, or a2i (Li et al., 2011; Shimizu et al., 2012). As SRC tyrosine kinase inhibition only partially reduces phosphorylation of ERK kinase (Shimizu et al., 2012), its effect on differentiation is not limited to convergent upstream inhibition of the FGF-ERK pathway. It has instead been suggested to block the epithelial-mesenchymal transition downstream of both the calcineurin-NFAT and the FGF-ERK pathways (Li et al., 2011) and stop differentiation by mechanical stress through an ERK-independent mechanism (Shimizu et al., 2012). Thus, the self-renewing pluripotent state of mESCs can be achieved through manipulation of key signaling pathways in vitro.

Despite sharing a common origin and defining properties, mESCs propagated under different culture conditions also differ (Ficz et al., 2013; Marks et al., 2012). For instance, serum/LIF-maintained mESCs are morphologically heterogeneous and show transcriptional fluctuation of certain pluripotency factors such as *Nanog* (Chambers et al., 2007; Kalmar et al., 2009), *Dppa3* (Hayashi et al., 2008), and *Rex1* (*Zfp42*) (Toyooka et al., 2008), unlike mESCs maintained in 2i conditions. These fluctuations have been proposed to represent a dynamic equilibrium between self-renewing and differentiation-poised states and thus be instrumental in regulating exit from pluripotency (Chang et al., 2008). However, others speculate that they arise through the use of fluorescent reporter systems and therefore are of unclear biological relevance (Chang et al., 2008; Faddah et al., 2013; Reynolds et al., 2012). The presence of transcriptionally heterogeneous subpopulations, prevalent bivalent chromatin domains, increased methylation content, and reduced RNA polymerase pausing compared to 2i mESCs has led to the notion that serum-maintained mESCs exist in a metastable pluripotent state (Marks et al., 2012), implying higher transcriptional cell-to-cell variation than the 2i state. Recently, a rare population of mESCs expressing markers of the two-cell stage of embryonic development was described (Macfarlan et al., 2012). These so-called 2C-like cells express the MERVL endogenous retrovirus and chimeric transcripts that arise via retroviral insertion in different places in the genome, and they are uniquely capable of differentiating into extraembryonic tissues. Our molecular understanding of the divergent pluripotent states, however, remains quite limited.

Single cell RNA-sequencing technology is increasingly used to deconstruct heterogeneous populations, lineage trajectories, and determinants of cell fate, questions that are central to the stem cell field (Etzrodt et al., 2014). Recently, Kumar et al. (2014) reported the single-cell transcriptome of serum/LIF-maintained mESCs and global transcriptome changes resulting from a range of chemical and genetic perturbations. Here, we performed single cell RNA-sequencing of mESCs cultured in serum/LIF, 2i/LIF, and the alternative ground state, a2i/LIF. This approach allowed us to compare the subpopulation structures and provide a deep characterization of cell-to-cell variation in gene expression levels across these three pluripotent states.

## Results

**Figure 1:**
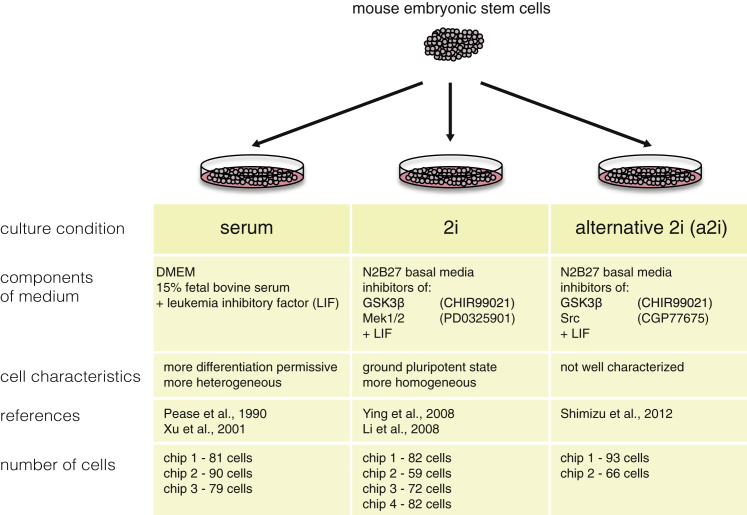
Experimental Scheme of Hybrid mESCs in Three Culture Conditions Schematic of experimental setup and cell culture conditions used in our study.

To examine features of gene expression heterogeneity across pluripotent states, we cultured an F1 hybrid (C57BL/6Ncr male x 129S6/SvEvTac female) mESC cell line (George et al., 2007) in three different conditions: (1) three replicates of serum + LIF, (2) four replicates of 2i + LIF, and (3) two replicates of a2i + LIF, which we will refer to as serum (serum1, serum2, and serum3), 2i (2i1, 2i2, 2i3, and 2i4) and a2i (a2i1 and a2i2) henceforth (Figure 1). In total, we collected 704 single-cell transcriptomes across these three conditions by using the Fluidigm C1 system and applying the SMARTer Kit to obtain cDNA and the Nextera XT Kit for Illumina library preparation.

After quality control analysis on each individual cell (Figures S1A–S1H), 250 serum cells, 295 2i cells, and 159 a2i cells remained. On average, we sequenced over 9 million reads per cell. Over 80% of reads mapped to the *Mus musculus* genome (GRCm38) and over 60% to exons (mapping overview in Figures S1G and S1H). We also performed standard bulk RNA-sequencing for each condition. As in previous studies, when we averaged gene expression levels across the single cells profiled in each condition, we observed that the mean expression levels recapitulated the bulk gene expression levels with a Spearman rank correlation coefficient of around 0.9 (Figures S1D and S1E).

### Transcriptome-wide Cell-to-Cell Variation Is Similar across the Three Culture Conditions

**Figure 2:**
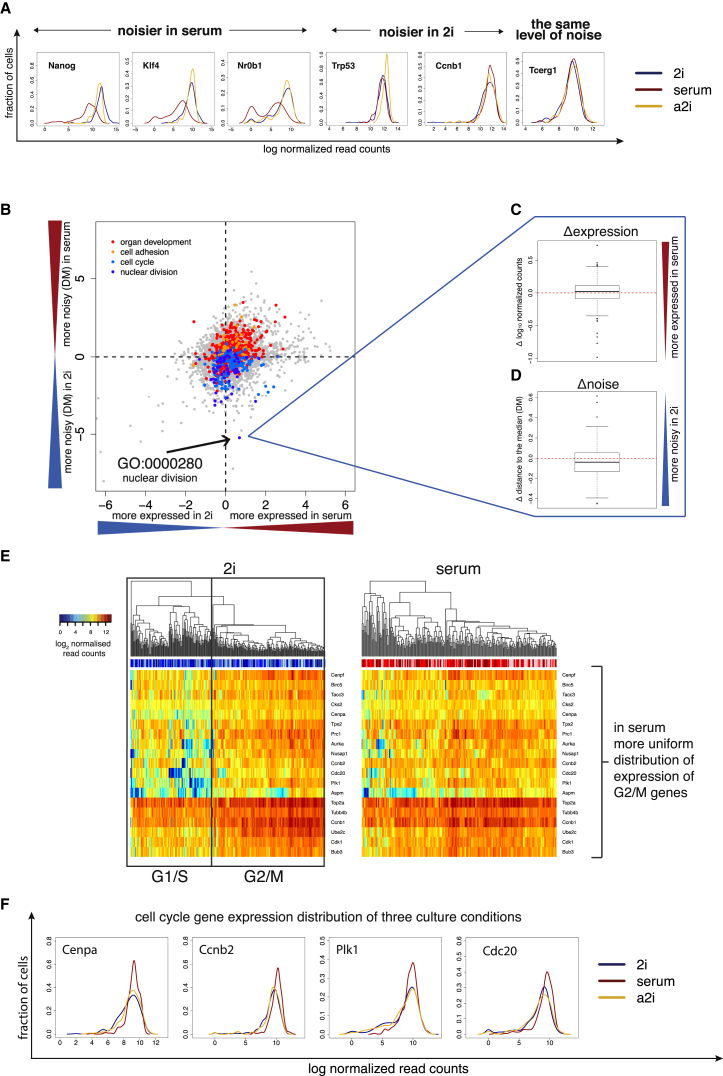
Global Cell-to-Cell Variation in Gene Expression (A) Gene expression distributions of genes, which are noisier in 2i than serum, that have similar noise profiles in serum (red), 2i (blue), and a2i (yellow). Distributions of gene expression were smoothed using the kernel density estimation function in R with default parameters. Tcerg1 does not have significantly different expression profiles between culture conditions (two-sided KS test p value for 2i and a2i comparison is 0.82, and for 2i and serum, 0.16). By contrast, other genes, such as Ccnb1, are more heterogeneous in 2i (p = 7 × 10^−4^ by two-sided KS test between 2i and serum), while some, such as Nanog, Klf4, or Nr0b1, are more heterogeneous in serum (p < 10^−15^ by two-sided KS test between 2i and serum for genes shown). (B) Comparison of the levels of gene expression and noise for gene ontology (GO) categories between serum and 2i (excluding 2i replicates containing 2C-like cells). The logarithm (log10) of p values from two-sided paired t tests applied to mean normalized read counts (x axis) and DMs (y axis) was computed for each GO category and plotted against each other by multiplying the sign of the t statistic. (C and D) Example of a GO category (GO:0000280, nuclear division) that is noisier in 2i (C) and is similarly expressed between the two conditions (D). (E) Heatmaps showing the expression of cell-cycle-related genes in serum and 2i, with a distinct separation into G1/S versus G2/M cells in 2i, with less distinction between individual cells in serum. (F) Gene expression profiles for key cell-cycle genes in all conditions show more heterogeneity in 2i.

An advantage of the single-cell approach is that we can study the distribution of expression levels across the population, thereby capturing cell-to-cell variability in gene expression (Figure 2A). To compare global levels of gene expression heterogeneity between the three different culture conditions, we used the coefficient of variation (CV) of normalized read counts (Figure S2). However, the CV of a gene depends strongly on its mean expression level and length, making it difficult to interpret differences between conditions. To account for the confounding factor of expression level, we therefore developed a measure of cell-to-cell variation by calculating the distance between the squared CV of each gene and a running median (Figures S2E and S2F). This is derived from the scatterplot of the mean normalized read counts versus the squared CV values, as in (Newman et al., 2006). We refer to this expression-level normalized measure of gene expression heterogeneity as distance to the median (DM) (refer to Supplemental Experimental Procedures for details).

Given the heterogeneous morphology of mESCs cultured in serum (Marks et al., 2012; Toyooka et al., 2008), as well as the heterogeneous expression of pluripotency factors (Canham et al., 2010; Hayashi et al., 2008; Kalmar et al., 2009; Singh et al., 2007), it was surprising that transcriptome-wide DM values are not significantly different across the three culture conditions (p = 0.625 by the Freidman rank sum test) (Figures S2B–S2D).

This prompted us to ask whether the levels of heterogeneity for genes belonging to individual functional categories are also consistent between conditions. We first performed gene set enrichment analysis for each culture condition to test whether genes belonging to Gene Ontology (GO) terms are enriched among those genes with extreme DM values. We observed that genes involved in translation, ribosome, RNA binding, structural molecule activity, and mRNA processing have a lower level of gene expression heterogeneity for all conditions (Figure S3D). In contrast, genes involved in plasma membrane, metal ion binding, lysosome, and integral component of membrane exhibit higher variation than expected by chance in all three conditions (p < 10^−4^). To gain more insight into how gene expression heterogeneity for functional categories differs between culture conditions, we compared the DM values of genes in pairs of culture conditions for each GO term (excluding 2i replicates containing 2C-like cells; for discussion of 2C-like cells, see below) (Figures 2B–2D, Figures S3A and S3B). We found that 712 GO terms (out of a total of 19,107 terms) exhibit a significant difference in levels of gene expression heterogeneity in at least one pairwise comparison (p < 0.01). For example, the expression of genes involved in “organ development” (p = 3.3 × 10^−4^) and “cell adhesion” (p = 4.8 × 10^−4^) is more heterogeneous in serum than in the inhibitory conditions (2i and a2i): these terms contain many pluripotency factors.

In contrast, genes involved in “cell cycle” (p = 5.4 × 10^−3^) and “nuclear division” (p = 5.9 × 10^−6^) have higher levels of gene expression heterogeneity in 2i compared to serum (Figures 2B–2D, Figures S3A and S3B). When we included 2i replicates containing 2C-like cells, we observed a similar trend (Figure S3C). When clustering cells based on cell-cycle genes only, we found that 2i cells separate into two groups: one with high expression of G2 and M genes and a second with lower expression of these genes (Figures 2E and 2F). Cells in serum and 2i also show different doubling kinetics with a rapid initial growth rate in 2i (24 hr). At the time of harvest, however (48 hr after plating), the doubling time of cells in 2i is 25 hr and in serum it is 11 hr, indicating that cells grown in 2i cycle more slowly, probably due to a longer G1 phase (refer to Supplemental Experimental Procedures).

As an independent validation, we performed the same analysis using data published previously (Grün et al., 2014). Consistent with our observations, global levels of gene expression heterogeneity between cells grown in 2i and in serum were comparable, while GO categories for development and differentiation were more heterogeneous in serum than in 2i, and cell-cycle genes were more heterogeneous in 2i than in serum (Figures S3E–S3G, Supplemental Experimental Procedures, Table S2).

### Three Subpopulations Can Be Delineated in Serum-Grown mESCs

**Figure 3:**
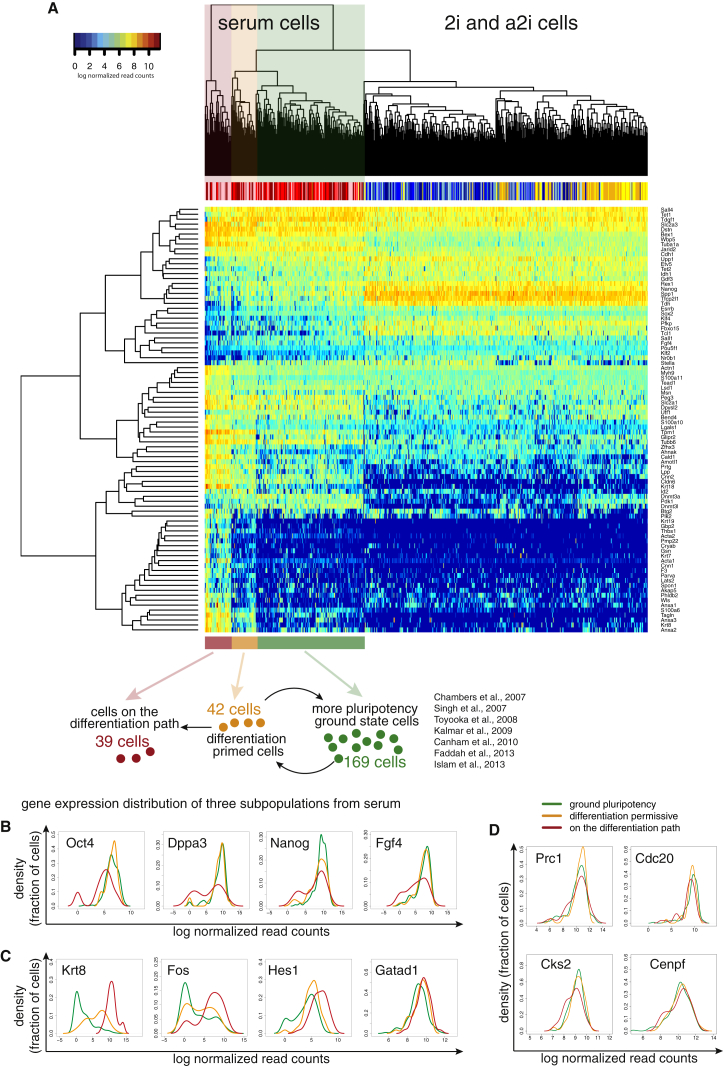
Population Structure in Serum, 2i, and a2i Cells (A) Clustering of cells in three culture conditions using a panel of pluripotency factors and differentiation markers. Correlations between cells and genes were calculated using Spearman correlation. Below the heatmap we show a model of the subpopulations of cells grown in serum. The schematic shows cells that express differentiation markers (red), cells that are primed for differentiation while remaining pluripotent (orange), and cells that are closest to the ground state of pluripotency (green). (B and C) Gene expression distributions of genes that become downregulated (B) and upregulated (C) upon differentiation. Expression is shown as log_2_ size factor normalized counts. Oct4 expression is similar in cells closer to the ground state of pluripotency (green) and cells that are primed for differentiation (yellow), and it is much lower in cells we defined as moving toward differentiation (red). (D) Gene expression distributions of cell-cycle genes.

Genes with heterogeneous expression, especially those with clear bimodal expression (Figure 2A), may indicate the existence of underlying subpopulations. Indeed, hierarchical clustering of subsets of known pluripotency genes and differentiation markers reveals that serum-grown cells split into three distinct groups (Figure 3A). Similar to others, we found heterogeneous expression of *Nanog* (Faddah et al., 2013; Kalmar et al., 2009; MacArthur et al., 2012; Singh et al., 2007), *Esrrb* (van den Berg et al., 2008), and *Zfp42* (Toyooka et al., 2008) in serum, as well as heterogeneous expression of *Nr0b1* and *Utf1.*

One subpopulation consists of 39 cells (15%) that express higher levels of markers of differentiation, for example *Fos* or *Hes1*, and high levels of cytoskeletal genes such as keratins (*Krt8* and *Krt18*), actins (*Acta1* and *Acta2*), and annexins (*Anxa1*, *Anxa2*, and *Anxa3*). At the same time, these 39 cells have low levels or no expression of transcription factors involved in the maintenance of pluripotency (e.g., *Nanog*, *Sox2*, and *Oct4*) (Figures 3B and 3C), suggesting that these cells have exited pluripotency and committed to differentiation. A second group consists of 42 cells (17%) with somewhat lower expression levels of some pluripotency genes, such as *Dppa3* and *Nanog*, and some expression of differentiation genes, yet high expression of *Oct4* and *Sox2*. These cells may correspond to a previously described “differentiation permissive” set (Chambers et al., 2007; Islam et al., 2014; Kalmar et al., 2009). The largest group, which consists of 169 cells (68%), expresses the highest levels of pluripotency factors and exhibits very low expression of keratins or actins (Figures 3B and 3C).

We observe that the 39-cell and 42-cell populations, which have begun to move forward on the differentiation pathway, have heterogeneous expression of cell-cycle genes (Figure 3D). A shift in the distribution of the expression of G2/M genes, such as Cks2 or Cdc20, toward lower levels suggests that there are relatively more G1/S cells in these two groups as well. We inferred that more differentiated cells have a relatively longer G1 phase, as we sample more cells in G1 from this subpopulation relative to more pluripotent cells. This indicates that the 39-cell and 42-cell subsets that we identified proliferate more slowly than Nanog-high ground state pluripotent cells (Figure 3D). Moreover, we performed principal component analysis (PCA) of our data together with cells from an mESC-to-NPC (neural progenitor cell) differentiation time course (Bibel et al., 2007). We observed that cells belonging to the differentiating subpopulation overlap with cells that are differentiating toward NPCs (Figure S4). This strongly supports our earlier hypothesis that these cells are indeed progressing down a differentiation pathway (Figure 3A).

### Ground State mESCs Cultured in Different Media Have Non-Overlapping Transcriptomes

**Figure 4:**
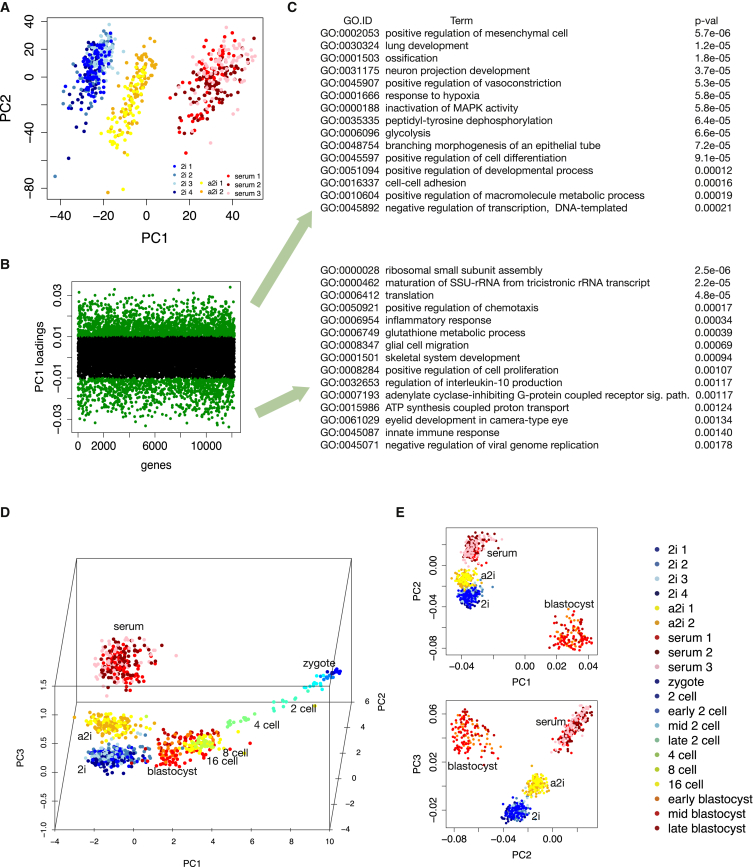
Clustering of mESCs Grown in Serum, 2i, and a2i Media (A) All cells (n = 704) grown in the three different culture conditions are projected onto the first two principal components. All genes with mean normalized read counts larger than ten were considered and principal component analysis (PCA) was performed. (B) Distribution of genes contributing to PC1. (C) GO enrichment analysis of genes most strongly contributing to PC1 separation. (D) PCA loading plot of the Spearman’s rank correlation coefficients from mESCs and single cells of mouse preimplantation embryos (Deng et al., 2014) showing the mapping of mESCs in mouse development stages. The cells are visualized by loadings of the first three principal components of the Spearman’s rank correlation matrix between cells, where we used the same expression cutoff as that employed by Deng et al. (E) PCA of Spearman’s rank correlation matrix between cells from three conditions and blastocyst. The first three components are shown.

Kalmar et al. (2009) suggested that mESCs grown in 2i are similar, or potentially identical, to the Nanog-high mESC subpopulation cultured in serum. To investigate whether ground state mESCs in serum (i.e. the cells we identified as most pluripotent within serum-only media) have a similar transcriptome to 2i or a2i mESCs, we clustered each population based on their global expression profiles. PCA (Figure 4A) demonstrates three separate clusters, revealing that ground state mESCs grown under different culture conditions in fact have distinct transcriptome identities. This is consistent with observations comparing bulk RNA-sequencing of Rex1-high (Zfp42-high) cells in serum and cells in 2i (Marks et al., 2012).

The Spearman correlation coefficient of mean gene expression levels between cells grown in the two inhibitory conditions is 0.95; between 2i and serum, it’s 0.88; and between a2i and serum, it’s 0.91. While these results suggest that 2i and a2i cells are more similar to each other than to serum-cultured cells, differences do exist between the two populations. Differences between 2i and a2i arise from the use of different inhibitors (Shimizu et al., 2012). Inhibition of Mek1/2 results in dephosphorylation of Erk1/2, while inhibition of Src does not have this effect, as we show by western blotting (Figure S1K).

To examine what differences in gene expression between the culture conditions explain the separation into distinct clusters, we performed GO enrichment analysis. We found that genes involved in development and differentiation, MAPK signaling, and basic metabolism are responsible for the separation (Figures 4B and 4C). To identify specific genes, we used DESeq, where each cell was considered a replicate of its culture condition, to test for significant differences in expression (Anders and Huber, 2010) as described in the Supplemental Experimental Procedures. There is a substantial amount of technical noise in single-cell data, so we considered only genes that are expressed on average above 50 normalized counts, as the technical bias is most pronounced for lowly expressed genes (Brennecke et al., 2013). This results in 4,587 differentially expressed genes between serum-grown cells and 2i-grown cells, 3,056 between serum and alternative 2i, and 2,061 genes between the two inhibitory conditions (the list of DE genes is available at http://www.ebi.ac.uk/teichmann-srv/espresso; Figure S5).

The two most enriched GO categories in genes differentially expressed between serum and 2i are in utero embryonic development (GO:0001701) and positive regulation of transcription from RNA polymerase II promoter (GO:0045944) (Table S1). Many of the transcription factors in the latter are key genes involved in pluripotency, such as *Nanog*, *Oct4*, *Klf4*, and *Sox2*. The differences between the two inhibitory conditions are smaller, and key terms are related to cell cycle, metabolism, and translation. Importantly, Oct4, Sox2, and Klf4 are not differentially expressed between 2i and a2i. Additionally, while Nanog is significantly differentially expressed, the expression level difference between 2i and a2i is smaller than between 2i and serum (log fold change = −0.71, adj p < 10^−6^ and log fold change = 2.4, adj p < 10^−98^, respectively). We observed the same pattern of differential gene expression in the bulk RNA-sequencing experiments (Table S1).

We hypothesized that differences between 2i and a2i, which are related to cell cycle and metabolism, may originate from different proportions of G1/S to G2/M phase cells in each condition. Indeed, using pre-defined cell cycle marker genes, we found that roughly 60% of cells in 2i are in G2/M and only 35% of cells are in G2/M in a2i. We therefore split the cells in each culture condition into G2/M and G1/S subgroups and compared G2/M cells from 2i with G2/M cells from a2i, and we did similarly for the G1/S subgroups. Subsequently, we considered the intersection of genes that were differentially expressed in each comparison and performed GO enrichment and KEGG pathway enrichment analyses. Overall, there are 97 genes with higher expression in 2i and 449 genes with higher expression in a2i. The genes that are upregulated in a2i are involved in RNA processing and transport, translation, and basic metabolism (Figure S6A).

The fact that, even after accounting for cell cycle, differentially expressed genes relate to basic cellular processes led us to explore whether cells cultured in a2i have more mRNA than cells cultured in 2i. To do this we exploited an external spike in molecules that we added to one batch of the cells (2i2, a2i2, and serum 3). The same number of molecules was added to each cell lysate, meaning that the ratio of all reads mapped to the spike ins to all reads mapped to exons can be considered as a proxy for cellular mRNA content (Ding et al., 2015; Stegle et al., 2015). Confirming the reliability of our approach, when we divided 2i and a2i cells into G1/S and G2/M subpopulations and compared their mRNA content, we found that cells in G2/M have significantly more mRNA than cells in G1/S (Figure S6B). Importantly, we observed that cells in 2i contain significantly fewer mRNA molecules than cells in serum and a2i (Wilcoxon test p < 10^−15^ for both comparisons), which supports the differential expression of genes involved in basic cellular processes.

### mESC Transcriptomes Are Similar, but Not Identical, to Those of Blastocyst Cells

It has been suggested that the pluripotent state of 2i cells resembles the cell state of early epiblast cells in the blastocyst (Boroviak et al., 2014; Nichols and Smith, 2009). The recent availability of single cell RNA-sequencing data from different stages of mouse embryonic development allowed us to assess the relationship of in vitro ESCs and in vivo blastomeres (Deng et al., 2014). Our cells were prepared with a very similar protocol, so we used PCA to overlay our data with the published embryonic time course. As expected, mESCs are most similar to the blastocyst stage cells from which they were derived, but they do not overlap (Figures 4D and 4E). The difference between in vivo blastocyst cells and cells cultured in 2i may originate from differences in the mouse strains and/or sequencing protocols, as well as transcriptome changes resulting from in vitro adaptation. mESCs grown in the inhibitory conditions are the most similar to the in vivo blastocyst cells, while serum cultured cells are somewhat more distant (Figures 4D and 4E), which has also been shown previously using cell ensembles (Boroviak et al., 2014).

The dispersion of mESCs in each culture condition is smaller than the dispersion between cells in the blastocyst. This may be explained by noting that mESCs are derived by clonal expansion and cultured in homogeneous conditions relative to the complexity of cellular niches within the embryo. Moreover, blastocyst cells were obtained from several embryos, thus adding an additional factor that may increase heterogeneity. We quantified global transcriptome noise using the DM measure to compare the heterogeneity of blastocyst cells from three stages (early, mid, and late) versus mESCs cultured in 2i. In all comparisons, blastocyst cells are significantly more heterogeneous than the cultured cells (p < 10^−4^ by Wilcoxon signed rank test).

### Identification and Characterization of 2C-like Cells in 2i Medium

**Figure 5:**
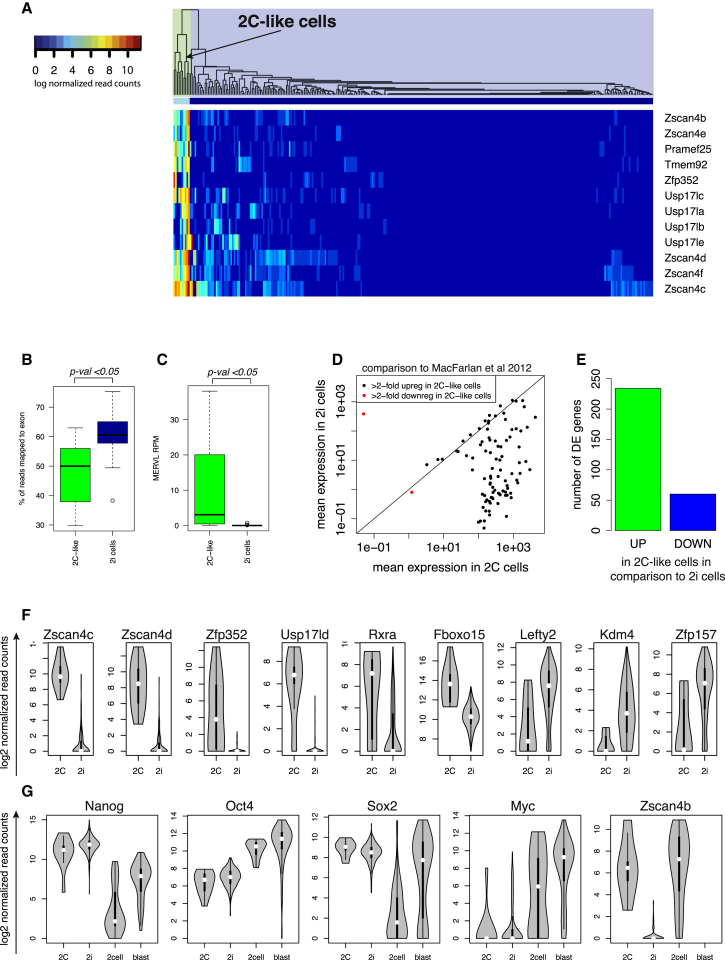
2C-like Population (A) Clustering of cells grown in 2i using markers of the 2C-like state (Macfarlan et al., 2012). Correlations were calculated using Spearman correlation. The dendrogram divides cells into two groups, one of which contains ten cells expressing 2C-markers. (B) Boxplot showing percentage of reads mapping to the exons in both subpopulations of cells in 2i. p was calculated using a Wilcoxon test. (C) Boxplot showing RPM (reads per million) mapping to the MERVL retrovirus in both subpopulations of cells in 2i. p was calculated using a Wilcoxon test. (D) Mean expression of genes reported to be at least 2-fold upregulated or downregulated in 2C-like cells (Macfarlan et al., 2012) in cells that we identified as 2C-like cells and in the remaining 2i cells. (E) Barplot showing the number of significantly (DESeq, adjusted p < 0.05) upregulated and downregulated genes in 2C-like cells. (F) Gene expression distributions of genes that become upregulated or downregulated in 2C-like cells (2C) in comparison to remaining cells grown in 2i media (2i). (G) Expression of key pluripotency genes in 2C-like cells (2C), the rest of cells grown in 2i media (2i), cells from the two-cell stage (2cell), and cells from the blastocyst stage (blast) of the embryo.

To find 2C-like cells in our samples, we examined the expression profile of genes shown previously to have at least 10-fold enrichment in 2C-like marker genes relative to the remaining mESCs (Macfarlan et al., 2012). Hierarchical clustering suggested the presence of ten 2C-like cells in 2i, and none in the a2i or serum culture conditions (2C-like cells may still be present in a2i and serum, but at a very low rate) (Figure 5A).

Globally, the transcriptomes of 2C cells are altered, and only about 50% of reads on average map to exons, compared to 60% in the remaining population in 2i (Figure 5B). Additionally, we observed substantial MERVL expression in 2C-like cells and no expression in the remaining cells (Figure 5C). Subsequently, we calculated the mean expression level of genes identified by Macfarlan et al. (2012) as differentially expressed in 2C-like cells and observed a similar pattern in our data (Figure 5D). Interestingly, we also observe that 2C-like cells have more upregulated genes than downregulated genes (Figure 5E).

It should be noted that globally, 2C-like cells are more similar to 2i cells and blastocyst cells than to cells from the two-cell stage of the in vivo embryo. 2C-like cells cluster together with 2i cells (Figure 5A), and there are only 294 differentially expressed genes between 2C-like cells and the remaining 2i cells (examples in Figure 5F). In comparison, we find 1,700 genes between 2C-like cells and blastocyst and 1,779 between 2C-like cells and two-cell stage cells (for differential expression results see http://www.ebi.ac.uk/teichmann-srv/espresso). In terms of expression of Nanog, Oct4, Sox2, and Myc, 2C-like cells are also similar to 2i cells in comparison to the two-cell and blastocyst stages of the embryo (Figure 5G).

### Transcriptional Regulatory Interactions in mESCs Revealed by Gene-to-Gene Correlations

Above, we mined our high-throughput single cell RNA-sequencing data from the perspective of comparing in vitro and in vivo pluripotent cell populations. We next examined its potential as a rich resource for analyzing correlations in gene expression across culture conditions as a strategy to identify candidate regulators of pluripotency. This allows us to develop hypotheses about the transcriptional regulatory networks that regulate pluripotency in mESCs, which is known to be highly interconnected and complex (Boyer et al., 2005; Kim et al., 2008; Loh et al., 2006).

**Figure 6:**
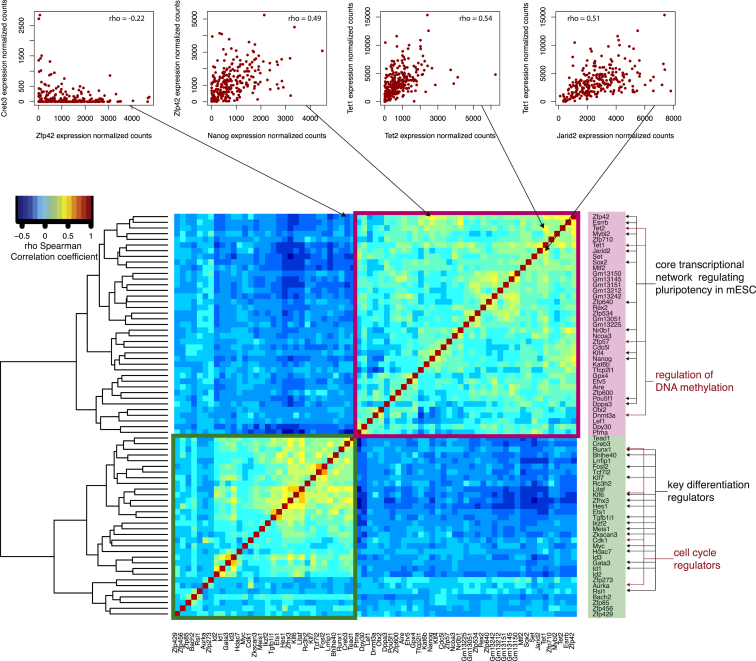
Spearman Correlation Matrix of Transcription Factors and Key Pluripotency Genes The heatmap shows the correlation coefficients between a set of transcription factors and other key genes involved in pluripotency. Above are examples of genes with expression patterns that correlate positively and negatively (from the left: Zfp42 and Creb3, Zfp42 and Nanog, Tet1 and Tet2, Tet1 and Jarid2).

We found that in serum-cultured mESCs, Nanog expression correlates positively with transcription factors (*Esrrb*, *Klf4*, *Oct4/Pou5f1*, *Sox2*, and *Zfp42*), genes involved in DNA methylation (*Dnmt3a*, *Tet1*, and *Tet2*), and other genes such as nuclear receptor *Nr0b1* and histone lysine acetyltransferase *Kat6b*. Nanog is negatively correlated with differentiation regulators including transcription factors *Gata3* and *Klf7* (Figure 6). These findings concur with known interactions in the pluripotency regulatory network, where *Nanog* regulates *Esrrb* (Boyer et al., 2005), *Zfp42* (Shi et al., 2006), and *Klf4* (Zhang et al., 2010). Beyond confirming known interactions, we identified correlations between characterized pluripotency genes and candidate components of the pluripotency transcriptional regulatory network.

**Figure 7:**
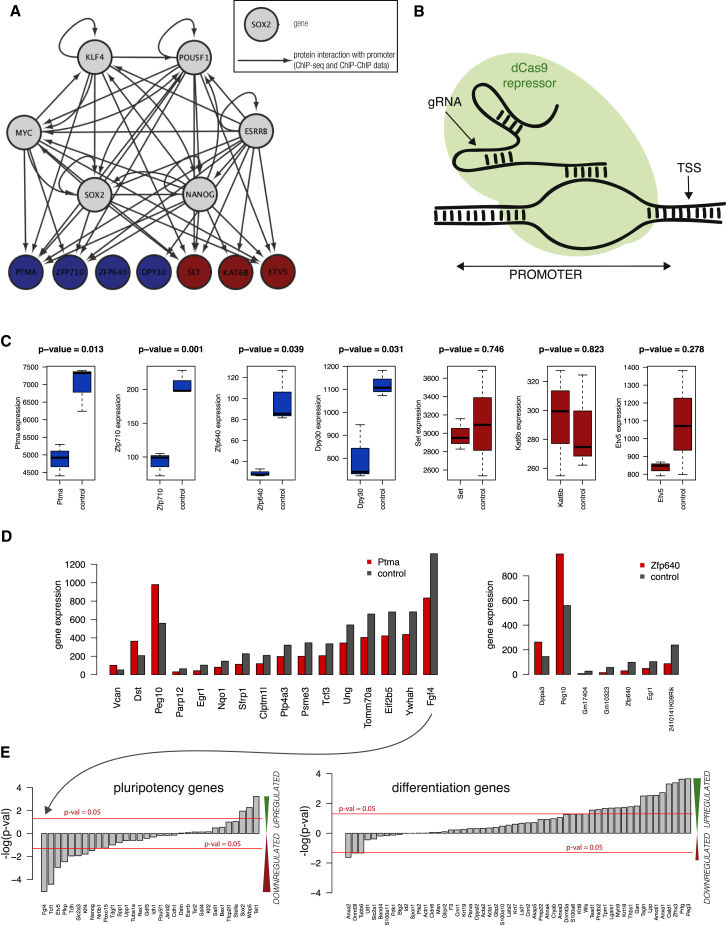
Validation of Putative Members of the Pluripotency Network (A) Network showing known interactions of core pluripotency factors with the novel candidate genes. Data obtained from ChIP-seq and ChIP-ChIP experiments from ESCAPE database. (B) Schematic showing experimental design. Catalytically inactive Cas9 and gRNA bind to the promoter of the targeted gene, occluding it and competing for binding with transcription factors and polymerases. (C) Expression level of repressed genes in samples and control. Targets with significant repression are in blue. (D) Barplot of gene expression levels of significantly differentially expressed genes in Ptma- and Zfp640-repressed samples (DESeq, multiple hypotheses testing adjusted p < 0.05). (E) Barplots showing the logarithm of p values for differential expression from DESeq of pluripotency (left) and differentiation (right) genes in the Ptma knockdown samples. For genes that are downregulated, the numbers are negative, and they are positive for upregulated genes. The red line indicates a p threshold of 0.05.

Of the candidate genes we selected seven genes for validation: Ptma, Zfp640, Zfp710, Dpy30, Set, Etv5, and Kat6b. First, using ChIP-seq and ChIP-ChIP data from the ESCAPE database, we found that the promoters of six of the candidate genes are bound by core pluripotency genes (Figure 7A) (Xu et al., 2013). To provide insight into the functional role of these genes, we downregulated their expression using a CRISPR/dCas9 repressor that targeted their promoters (Figure 7B) (Gao et al., 2014) before examining changes in their transcriptomes using bulk RNA-sequencing.

We narrowed down our analysis to four cases that showed significant repression of the targeted gene (Figure 7C) and performed differential expression analysis between samples and control gRNA using DESeq. After correcting for multiple hypothesis testing, we found significantly differentially expressed genes in two cases: Ptma and Zfp640 (Figure 7E). In the samples with repressed Ptma, we observed a decrease in the expression of pluripotency genes and an increase in the expression of genes associated with differentiation (Figure 7D, where pluripotency and differentiation genes are as in Figure 3). Zfp710 and Zfp640 show a similar but milder phenotype, while for Dpy30 there is no clear change in the expression of pluripotency genes (Figure S6C). The lack of effect of Dpy30 downregulation on pluripotency gene expression is consistent with a previous report (Jiang et al., 2011). Overall, these results suggest that Ptma and Zfp640, and potentially also Zfp710, are candidate genes involved in regulating the exit from pluripotency.

## Discussion

Here, using single cell RNA-sequencing, we quantified features of cell-to-cell gene expression heterogeneity in mESCs cultured in three different culture conditions. Previous studies had assumed, based on expression of key pluripotency genes, that cells cultured in mESCs are more heterogeneous. Surprisingly, we found that on a global level, cells grown in 2i, a2i, and serum are indistinguishable in terms of transcriptome-wide heterogeneity. Gene expression heterogeneity in specific subsets of genes instead uniquely defines each pluripotent state.

Our results show that mESCs form transcriptomically distinct cell populations depending upon the growth medium (serum, 2i, or a2i), with cells cultured in 2i and a2i being the most similar to each other. When compared to single cells from different stages of mouse embryonic development, all three sets of cultured mESCs are closest to cells from the blastocyst stage, which is the stage from which the cells were extracted originally. The 2i and a2i cultured ESCs seem more similar to the blastocyst cells than serum cells. Additionally, we observed that 2C-like cells are globally more similar to blastocysts than to two-cell stage embryonic cells.

Recently, single cell RNA-sequencing of serum-grown mESCs (Islam et al., 2014) showed a subpopulation with low *Nanog* expression. Additionally, a qPCR study using a panel of 48 pluripotency markers showed that cells cultured in serum exist in two distinct states, with a small number of cells appearing to reside in an intermediate state (Papatsenko et al., 2015). We extended this analysis to identify two smaller subsets of differentiated-committed and intermediate mESCs and a larger self-renewing population. The first shows clear downregulation of *Oct4* and *Sox2* and a slower cell cycle, suggestive of irreversible commitment. In contrast, the intermediate population with higher expression of *Oct4* and *Sox2* may retain the capacity to reacquire pluripotency. Importantly, we also found that the mESC subset that expresses high levels of Nanog in serum is not similar to “ground state pluripotency” 2i cells.

a2i medium has been described as an alternative ground state that can be achieved through the use of a different inhibitor (Shimizu et al., 2012). As expected, a2i is not identical to 2i, but we believe that it is rightfully called an alternative ground state: on the transcriptome level, especially with respect to pluripotency genes, a2i cells are similar to 2i and in vivo blastocyst cells. In 2i and a2i media, there are no subpopulations of differentiating mESCs; hence, pluripotency genes are expressed more homogeneously. Despite these similarities, it is intriguing to note that a2i cells have a cellular RNA content similar to serum-cultured cells, while 2i cells contain about half as much RNA on average, independent of cell-cycle stage. It should be noted that Myc is differentially upregulated in a2i cells compared to 2i cells. As Myc has recently been shown to behave as a transcriptional amplifier of active genes (Lin et al., 2012; Nie et al., 2012), it provides a potential mechanistic basis for the elevated mRNA content in a2i cells.

We observed a relationship between variability in the expression levels of cell-cycle genes and the length of the cell cycle. mESCs cultured in serum have the lowest level of gene expression heterogeneity and mESCs in 2i have the highest, which correlates negatively with doubling times in culture (doubling times were quickest for serum and slowest for 2i). For dividing populations where the cell cycle is very slow, such as HSCs, it is possible to assign cells to one of four cell-cycle stages, but this is more challenging for cells that cycle more quickly (Tsang et al., 2015).

In 2i, but not in a2i, we observed a subpopulation of 2C-like cells that also contribute to heterogeneity within the 2i population. As they are similar to the majority of 2i cells and rare, their contribution to the global heterogeneity of 2i cells is much smaller than the three distinct subpopulations in serum. It is worth noting that our results show that 2C-like cells are not particularly similar to cells at the two-cell stage of the embryo, as was suggested previously.

Finally, our data and methodology allowed us to find new genes involved in the pluripotency network, which we validated using CRISPR repression. We found that downregulating Zfp640, Zfp710, and Ptma affected the expression of both pluripotency and differentiation genes. Ptma repression resulted in the strongest deviation from control samples, and we infer that these cells deviate from pluripotency toward a differentiated state. Interestingly, Ptma is a well-known gene encoding prothymosin alpha, which upon cleavage becomes thymosin alpha, a peptide that has been well studied in the context of immunity and that is used in the treatment of Hepatitis B and C and cancer (Ciancio and Rizzetto, 2010; Garaci et al., 2012; Ioannou et al., 2012). The mode of action of Ptma has been studied in cancer and immune cells, and it has been shown to play a role in proliferation through mechanisms involving chromatin remodeling and interaction with numerous pathways associated with pluripotency maintenance such as the JAK-STAT pathway, the PI3K-Akt pathway, and the NF-κB pathway (George and Brown, 2010; Guo et al., 2015; Yang et al., 2004).

In summary, single-cell transcriptomics has allowed us to gain deep insights into the subpopulation structure within mESC cultures. These results emphasize the power of transcriptomics at single-cell resolution for understanding multiple biological processes.

## Experimental Procedures

### Cell Culture of mESCs

The G4 (C57BL/6Ncr x 129S6/SvEvTac) mouse hybrid (George et al., 2007) ESCs were obtained from Mount Sinai Hospital and were maintained on STO feeders in serum-containing media at 5% CO_2_ and 37°C. They were sub-cloned, and a line with normal karyotype was selected for further analysis. The cells were split onto gelatinized plates (10 cm, Corning) and expanded in serum-containing media or chemically defined media (standard 2i or alternative 2i) for at least three passages. Cells were harvested by trypsinization (0.05% trypsin/EDTA, GIBCO) for 10 min, at which point they reached 70%–80% confluence for single-cell capture.

The three media are as follows:

(1) Serum-containing media: Knockout DMEM (GIBCO), 1X penicillin-streptomycin-glutamine (GIBCO), 1X non-essential amino acids (GIBCO), 100 U/ml recombinant human leukemia inhibitory factor (Millipore), 15% fetal bovine serum (HyClone), 0.1mM β-mercaptoethanol (Sigma).
(2) Standard 2i media: N2B27 basal media (NDiff 227, StemCells), 100 U/ml recombinant human LIF (Millipore), 1 μM PD0325901 (Stemgent), 3 μM CHIR99021 (Stemgent).
(3) Alternative 2i media: N2B27 basal media (NDiff 227, StemCells), 100 U/ml recombinant human LIF (Millipore), 1 μM CGP77675 (Sigma), 3 μM CHIR99021 (Stemgent).

### cDNA Library Preparation from Single Cells using the Fluidigm C1

For each culture condition, 4,000 cells were loaded on to a 10–17 μm Fluidigm C1 Single-Cell Auto Prep IFC, and cell capture was performed according to the manufacturer’s instructions. The capture efficiency was inspected using a microscope to remove samples from the analysis with more than one cell captured. Upon capture, reverse transcription and cDNA preamplification were performed in the 10–17 μm Fluidigm C1 Single-Cell Auto Prep IFC using the SMARTer PCR cDNA Synthesis Kit (Clontech) and the Advantage 2 PCR Kit (Ramsköld et al., 2012). cDNA was harvested and diluted to a range of 0.1–0.3 ng/μl and Nextera libraries were prepared using the Nextera XT DNA Sample Preparation Kit and the Nextera Index Kit (Illumina) following the instructions in the Fluidigm manual “Using the C1™ Single-Cell Auto Prep System to Generate mRNA from Single Cells and Libraries for Sequencing.” Libraries from one chip were pooled, and paired-end 100 bp sequencing was performed on four lanes of an Illumina HiSeq2000.

### Bulk RNA-Sequencing

Bulk RNA-sequencing libraries were prepared and sequenced using the Wellcome Trust Sanger Institute sample preparation pipeline with Illumina’s TruSeq RNA Sample Preparation v2 Kit. RNA was extracted from 1–2 million cells using the QIAGEN RNA Purification Kit on a QiaCube robot. The quality of the RNA sample was checked using gel electrophoresis. For library preparation, poly-A RNA was purified from total RNA using oligo-dT magnetic pull-down. Subsequently, mRNA was fragmented using metal-ion catalyzed hydrolysis. The cDNA was synthesized using random hexamer priming, and end repair was performed to obtain blunt ends. A-tailing was done to enable subsequent ligation of Illumina paired-end sequencing adapters, and samples were multiplexed at this stage. The resulting library was amplified using 10 cycles of PCR, substituting the Kapa Hifi polymerase for the polymerase in the Illumina TruSeq Kit. Samples were diluted to 4nM, and 100 bp paired-end sequencing was carried out on an Illumina HiSeq2000. Sequencing Quality Control was performed by the Sanger sequencing facility.

### Mapping Reads

Paired-end reads were mapped simultaneously to the *Mus musculus* genome (Ensembl version 38.73) using GSNAP (version gmap-2014-05-15_v2) using default parameters. Subsequently we counted reads for each gene with htseq-count and normalized them with size factors calculated from DESeq as reported previously (Brennecke et al., 2013). We also applied location and scale adjustments to the normalized read counts to remove technical variation among multiple batches (Supplemental Experimental Procedures).

### Quality Control of Cells

To exclude poor quality cells from the downstream analysis, we removed cells according to the following criteria: (1) empty capture sites or capture sites with multiple cells or debris, as defined by visual inspection of the chip; (2) cells that had fewer than 500,000 reads mapped to exons; and (3) cells that had over 10% reads mapped to mitochondrial genes (refer to Supplemental Experimental Procedures for details).

### Candidate Gene Expression Repression with CRISPR

45 guide RNAs targeting promoter regions of 7 candidate genes (Ptma, Set, Zfp640, Zfp710, Kat6b, Dpy30, and Etv5) were cloned into gRNA-mCherry plasmid (for a list of sequences, refer to Table S3). GFP-Oct4 reporter strain ESCs (Silva et al., 2008) were transfected with (1) Cas9 repressor-BFP, (2) transposase, and (3) a cocktail of gRNA plasmids (Gao et al., 2014) targeting the gene of interest in a 1:1 ratio using Lipofectamine2000 (Life Technologies). Subsequently, cells were cultured in medium containing 15% serum and LIF for 4 days before 10,000 mCherry and BFP-positive cells were sorted for each sample. RNA was extracted using QIAGEN RNeasy Mini Kit. The SmartSeq2 protocol was used for reverse transcription and amplification of cDNA (Picelli et al., 2014). Sequencing libraries were prepared using Nextera XT Kit according to the manufacturer’s guidelines, barcoded with Nextera XT Dual Index Kit, and sequenced on an Illumina HiSeq2500 in rapid mode.

## Author Contributions

A.A.K. carried out single-cell and bulk RNA-sequencing experiments, analyzed and interpreted data, and prepared figures and the manuscript; J.K.K. developed and carried out statistical and bioinformatics analyses and figure preparation and contributed to manuscript preparation; J.T. cultured cells, performed the doubling time experiment, designed the CRISPR repression experiment, interpreted data, and contributed to the manuscript; T.I. carried out bioinformatics and statistical analyses, including that involved in figure preparation; J.H. developed the website; K.N.N. performed NPC single-cell differentiation time course experiments; A.C.T. performed NPC single-cell differentiation time course experiments; X.G. performed CRISPR repression experiments; M.B. supported NPC single-cell differentiation time course experiments; P.L. advised on cell culture conditions and ESC biology; J.C.M. developed and advised on statistics and bioinformatics methods and analysis and contributed to manuscript preparation; and S.A.T. designed experiments, advised on analysis, and contributed to manuscript preparation.

## Accession Numbers

Sequencing data are available in the ArrayExpress database (http://www.ebi.ac.uk/arrayexpress) under accession number E-MTAB-2600.

## Supplemental Information

Document S1. Figures S1–S7 and Supplemental Experimental Procedures

Table S1. Enriched Gene Ontology Categories in Each Condition, Related to Figure 4 Results of Gene Ontology analysis of differentially expressed genes in three comparisons: serum with 2i, 2i with a2i, and serum with a2i.

Table S2. Monoallelic Genes, Related to Figure 1 List of genes that exhibit monoallelic expression in all culture conditions.

Table S3. gRNA Sequences, Related to Figure 7 Primer sequences used for cloning into gRNA vector.

Document S2. Article plus Supplemental Information
