# Supplementary material for: Single Cell RNA-Sequencing of Pluripotent States Unlocks Modular Transcriptional Variation
Source: Cell Stem Cell. 2015 Oct 1;17(4):471–85. doi: 10.1016/j.stem.2015.09.011 (PMC4595712; doi:10.1016/j.stem.2015.09.011)
Supplement: Document S1. Figures S1–S7 and Supplemental Experimental Procedures [file mmc1.pdf]

Cell Stem Cell

Supplemental Information

Single Cell RNA-Sequencing of Pluripotent States Unlocks Modular Transcriptional Variation

Aleksandra A. Kolodziejczyk, Jong Kyoung Kim, Jason C.H. Tsang, Tomislav Ilicic, Johan Henriksson, Kedar N. Natarajan, Alex C. Tuck, Xuefei Gao, Marc Bühler, Pentao Liu, John C. Marioni, and Sarah A. Teichmann

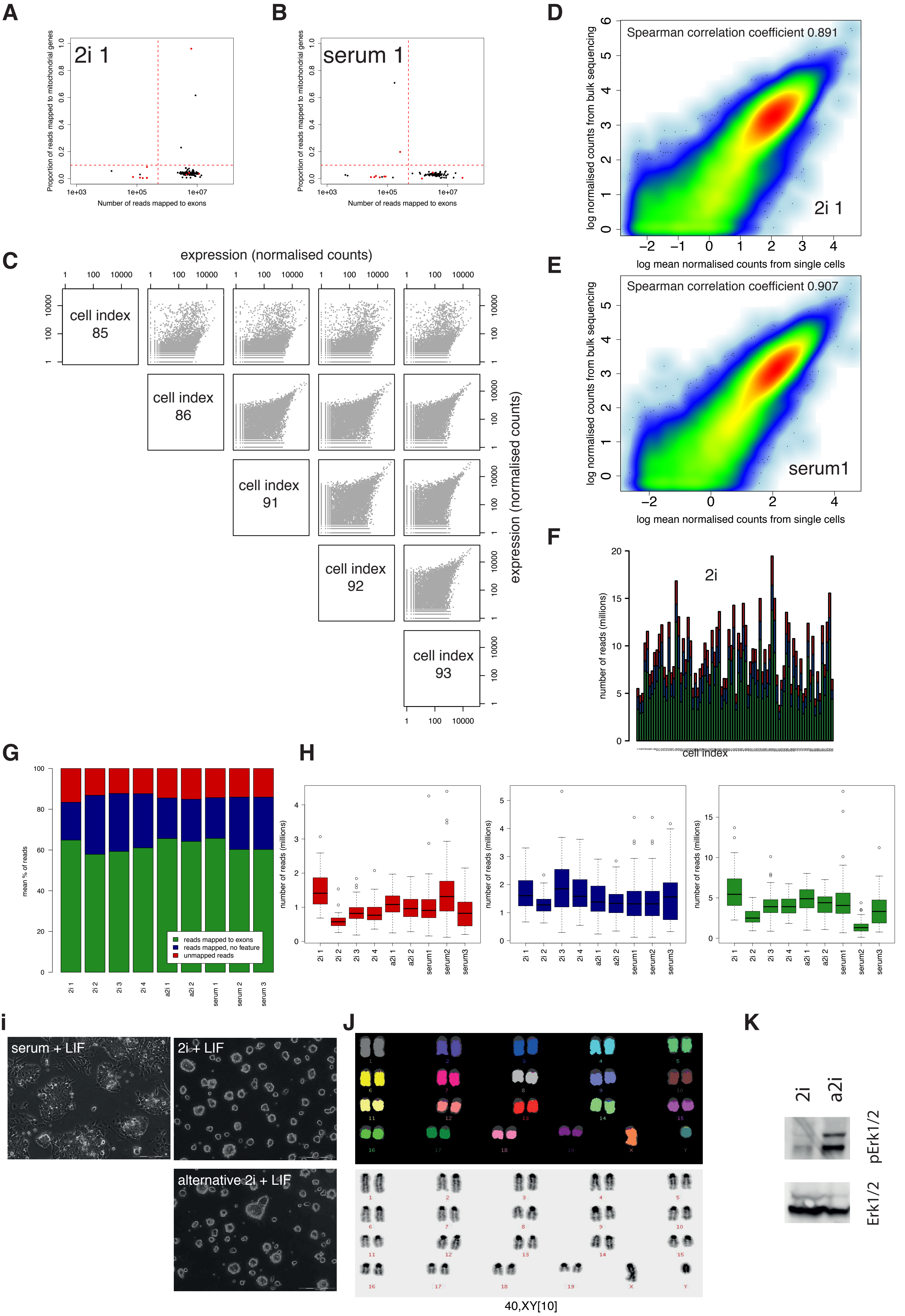

A
2i 1
Proportion of reads mapped to mitochondrial genes
Number of reads mapped to exons
B
serum 1
D
Spearman correlation coefficient 0.891
log normalised counts from bulk sequencing
log mean normalised counts from single cells
2i 1
C
expression (normalised counts)
cell index 85
cell index 86
cell index 91
cell index 92
cell index 93
E
Spearman correlation coefficient 0.907
serum1
F
2i
number of reads (millions)
cell index
G
mean % of reads
reads mapped to exons
reads mapped, no feature
unmapped reads
2i 1
2i 2
2i 3
2i 4
a2i 1
a2i 2
serum 1
serum 2
serum 3
H
number of reads (millions)
i
serum + LIF
2i + LIF
alternative 2i + LIF
J
40,XY[10]
K
2i
a2i
pErk1/2
Erk1/2

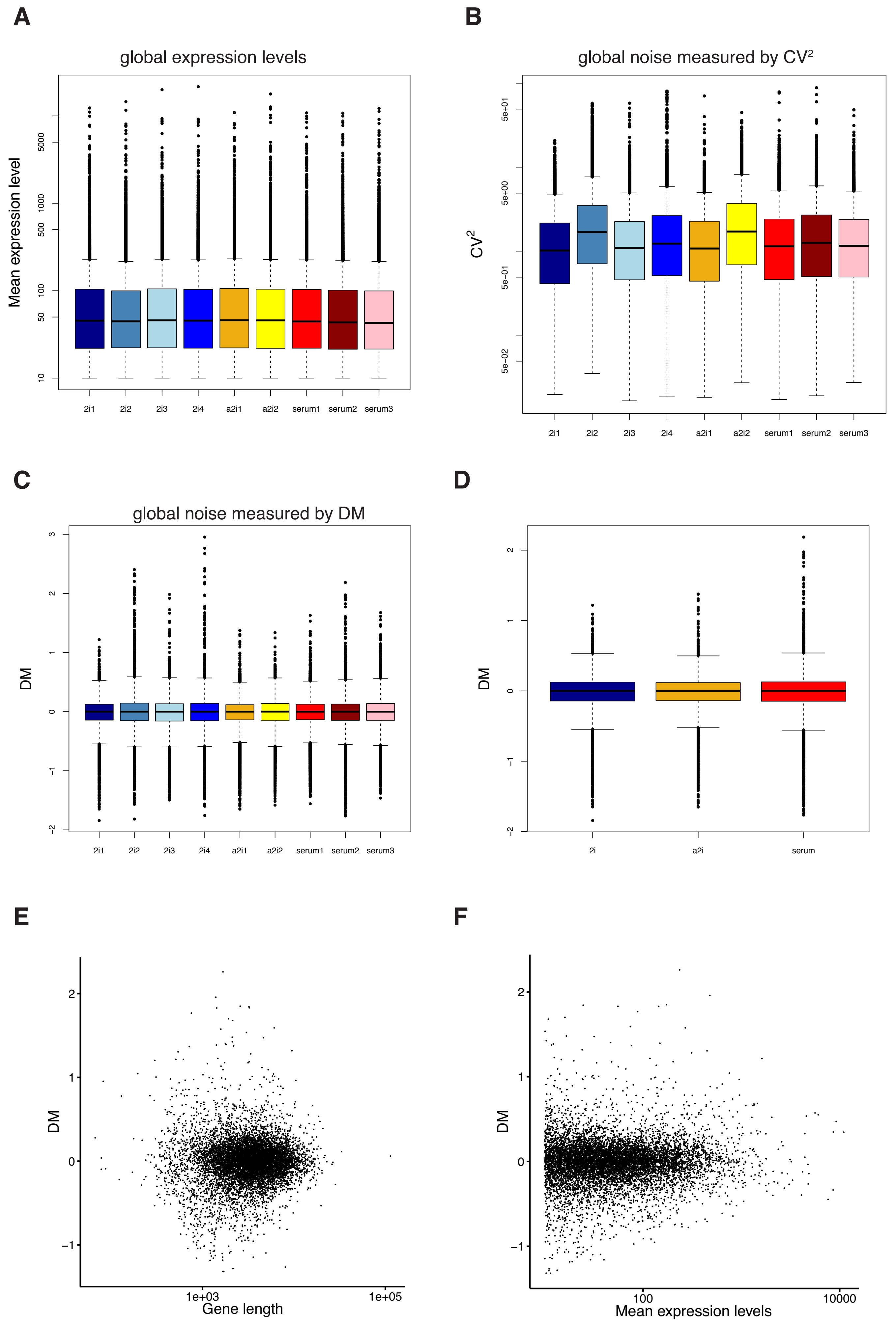

A
global expression levels
Mean expression level
10
50
100
500
1000
5000
2i1
2i2
2i3
2i4
a2i1
a2i2
serum1
serum2
serum3
B
global noise measured by CV²
CV²
5e−02
5e−01
5e+00
5e+01
2i1
2i2
2i3
2i4
a2i1
a2i2
serum1
serum2
serum3
C
global noise measured by DM
DM
−2
−1
0
1
2
3
2i1
2i2
2i3
2i4
a2i1
a2i2
serum1
serum2
serum3
D
DM
−2
−1
0
1
2
2i
a2i
serum
E
DM
−1
0
1
2
1e+03
1e+05
Gene length
F
DM
−1
0
1
2
100
10000
Mean expression levels

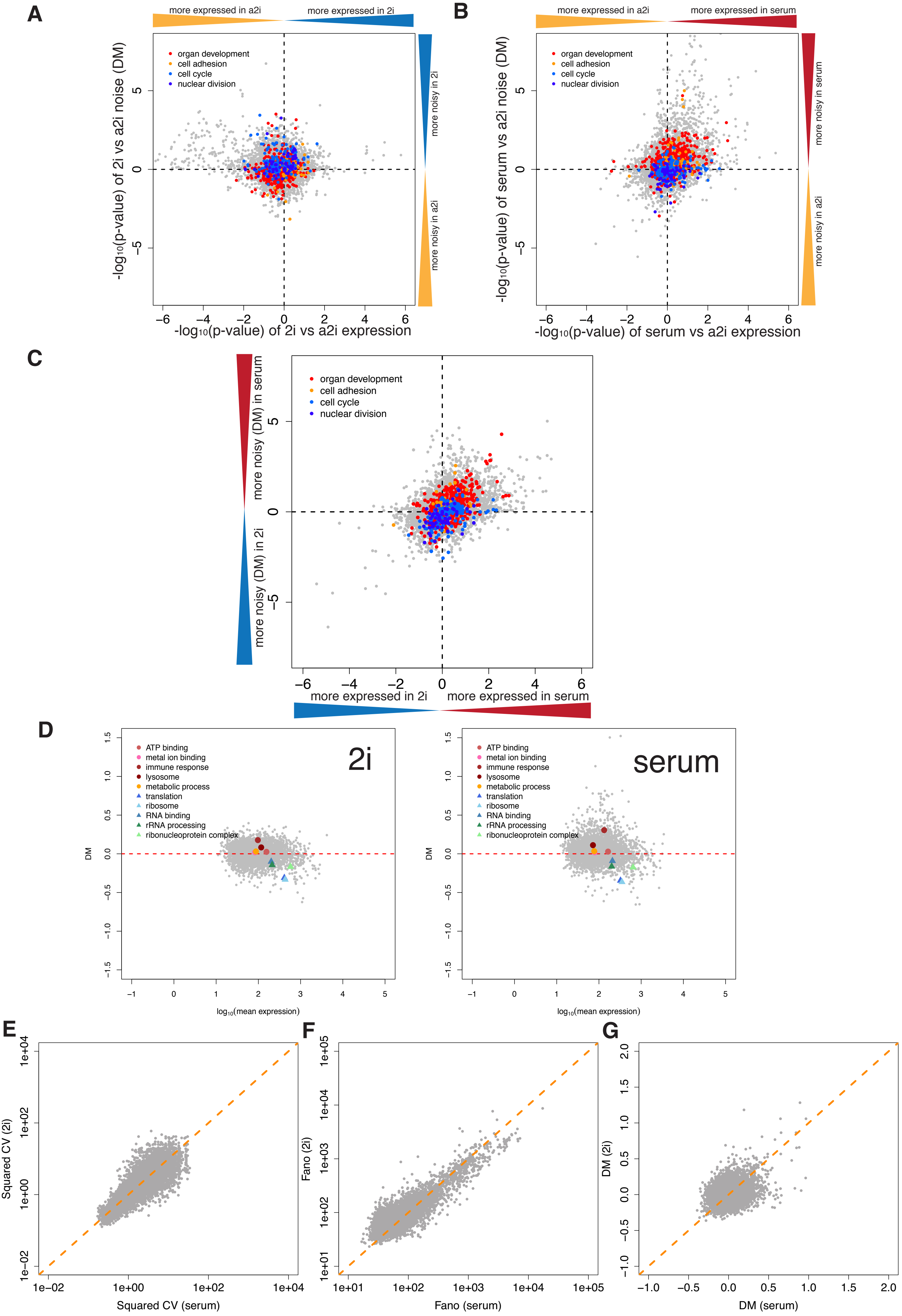
A
more expressed in a2i
more expressed in 2i
more noisy in 2i
more noisy in a2i
organ development
cell adhesion
cell cycle
nuclear division
-log10(p-value) of 2i vs a2i noise (DM)
-log10(p-value) of 2i vs a2i expression
B
more expressed in a2i
more expressed in serum
more noisy in serum
more noisy in a2i
-log10(p-value) of serum vs a2i noise (DM)
-log10(p-value) of serum vs a2i expression
C
more noisy (DM) in serum
more noisy (DM) in 2i
more expressed in 2i
more expressed in serum
D
ATP binding
metal ion binding
immune response
lysosome
metabolic process
translation
ribosome
RNA binding
rRNA processing
ribonucleoprotein complex
2i
serum
DM
log10(mean expression)
E
Squared CV (2i)
Squared CV (serum)
F
Fano (2i)
Fano (serum)
G
DM (2i)
DM (serum)

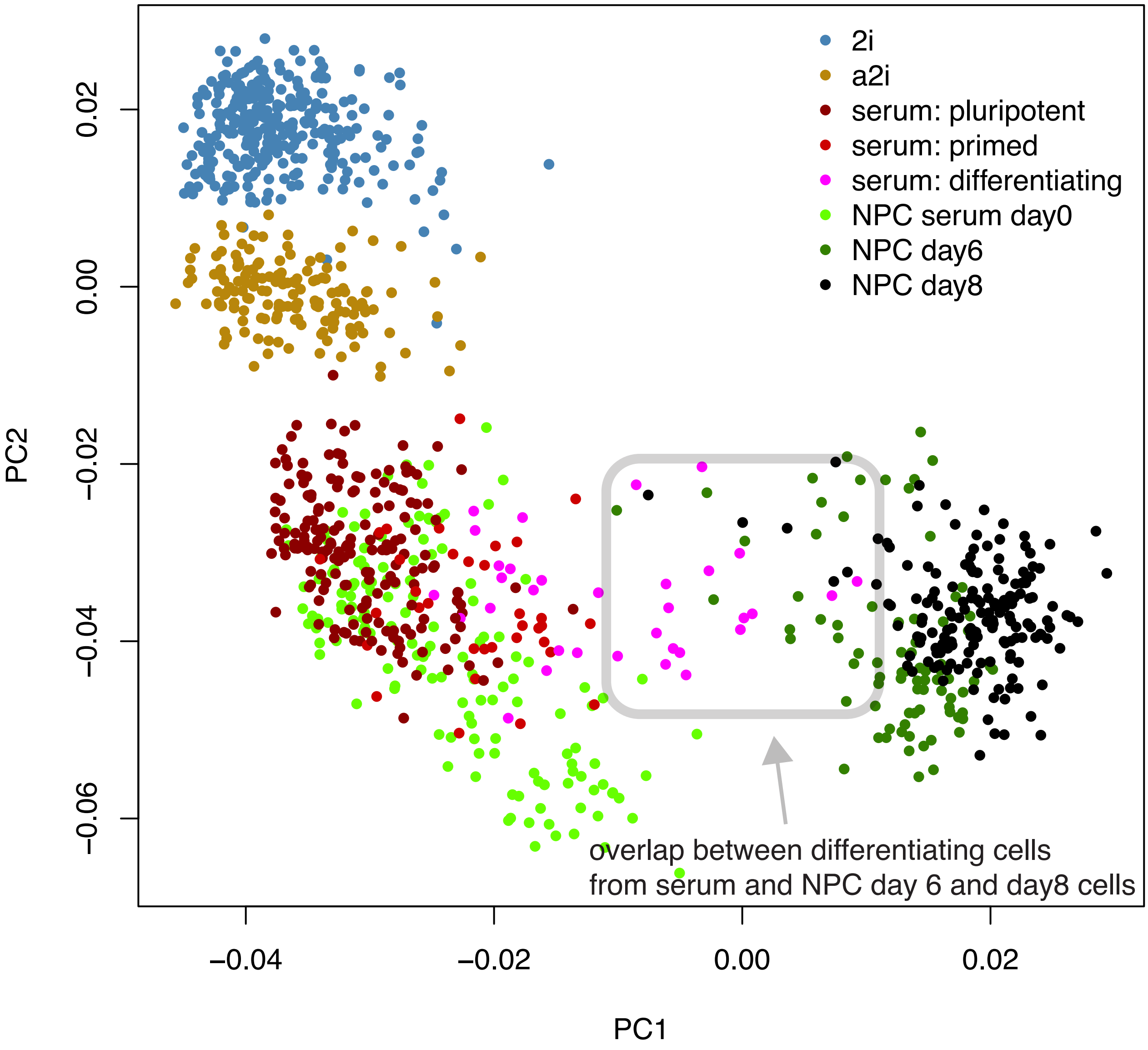
2i
a2i
serum: pluripotent
serum: primed
serum: differentiating
NPC serum day0
NPC day6
NPC day8
overlap between differentiating cells
from serum and NPC day 6 and day8 cells
PC2
0.02
0.00
−0.02
−0.04
−0.06
PC1
−0.04
−0.02
0.00
0.02

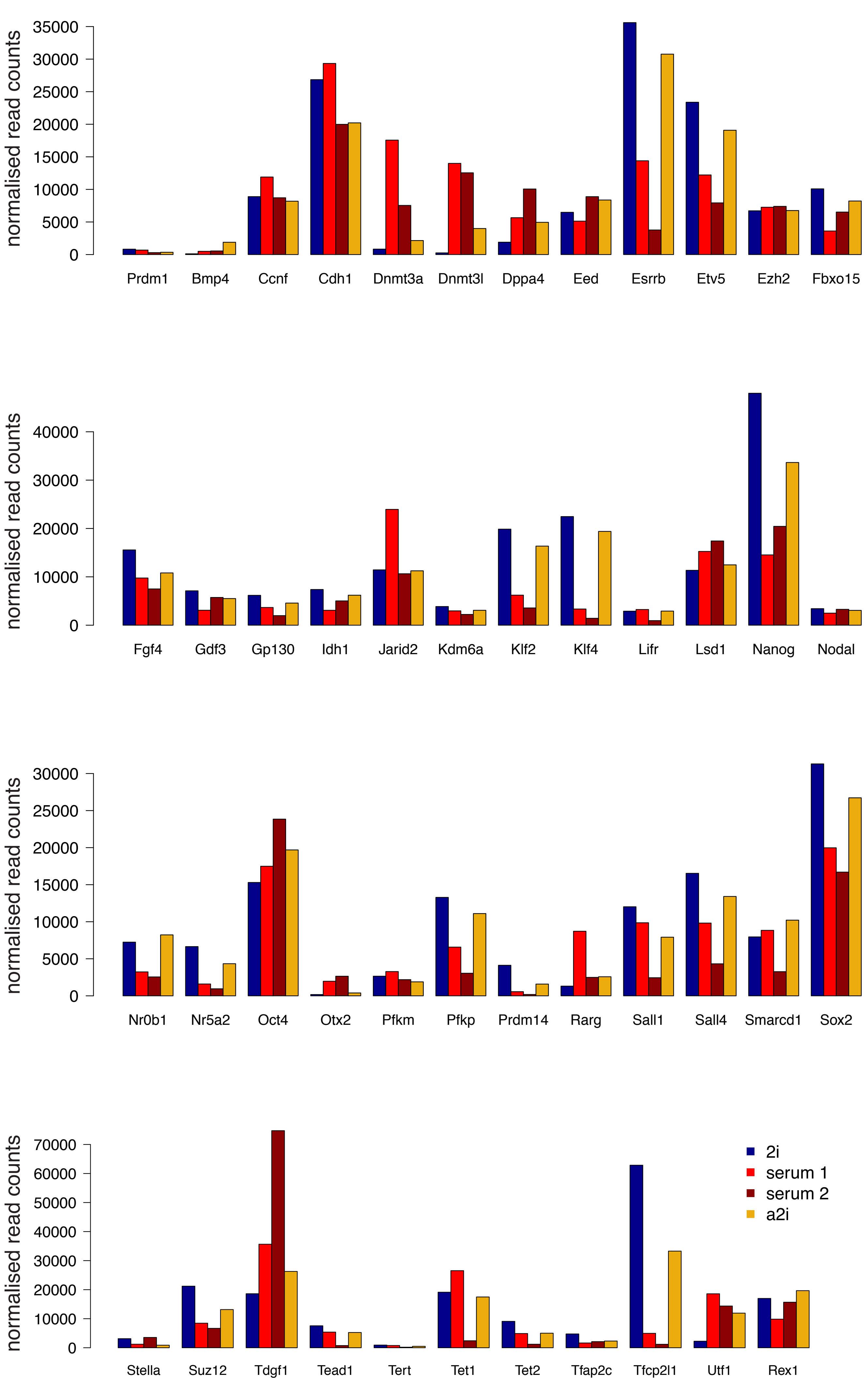
normalised read counts
35000
30000
25000
20000
15000
10000
5000
0
Prdm1
Bmp4
Ccnf
Cdh1
Dnmt3a
Dnmt3l
Dppa4
Eed
Esrrb
Etv5
Ezh2
Fbxo15
normalised read counts
40000
30000
20000
10000
0
Fgf4
Gdf3
Gp130
Idh1
Jarid2
Kdm6a
Klf2
Klf4
Lifr
Lsd1
Nanog
Nodal
normalised read counts
30000
25000
20000
15000
10000
5000
0
Nr0b1
Nr5a2
Oct4
Otx2
Pfkm
Pfkp
Prdm14
Rarg
Sall1
Sall4
Smarcd1
Sox2
normalised read counts
70000
60000
50000
40000
30000
20000
10000
0
Stella
Suz12
Tdgf1
Tead1
Tert
Tet1
Tet2
Tfap2c
Tfcp2l1
Utf1
Rex1
2i
serum 1
serum 2
a2i

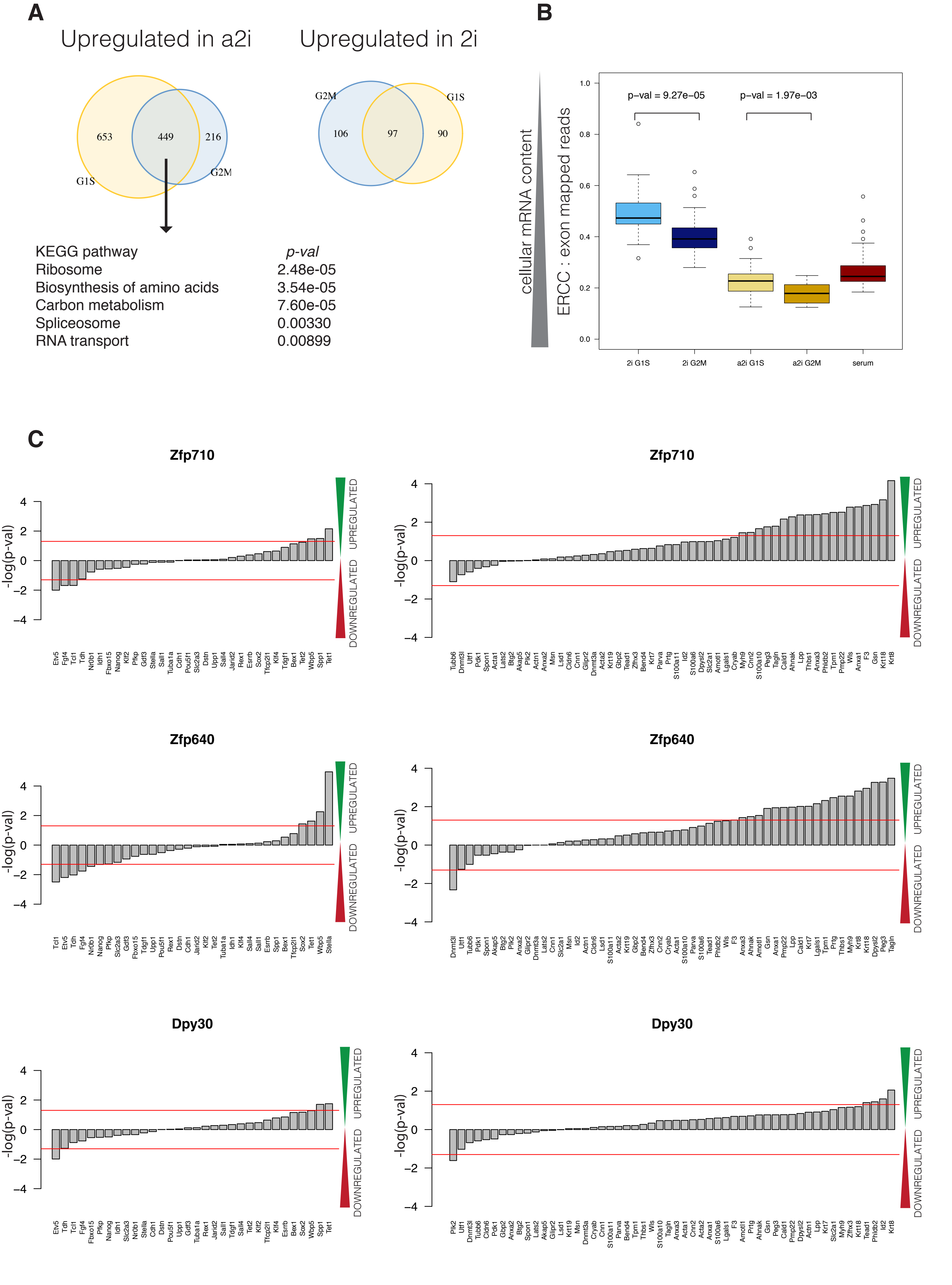
A
Upregulated in a2i
Upregulated in 2i
653
449
216
G1S
G2M
G2M
G1S
106
97
90
KEGG pathway
p-val
Ribosome 2.48e-05
Biosynthesis of amino acids 3.54e-05
Carbon metabolism 7.60e-05
Spliceosome 0.00330
RNA transport 0.00899
B
cellular mRNA content
ERCC : exon mapped reads
p-val = 9.27e-05
p-val = 1.97e-03
2i G1S
2i G2M
a2i G1S
a2i G2M
serum
C
Zfp710
Zfp640
Dpy30
-log(p-val)
UPREGULATED
DOWNREGULATED

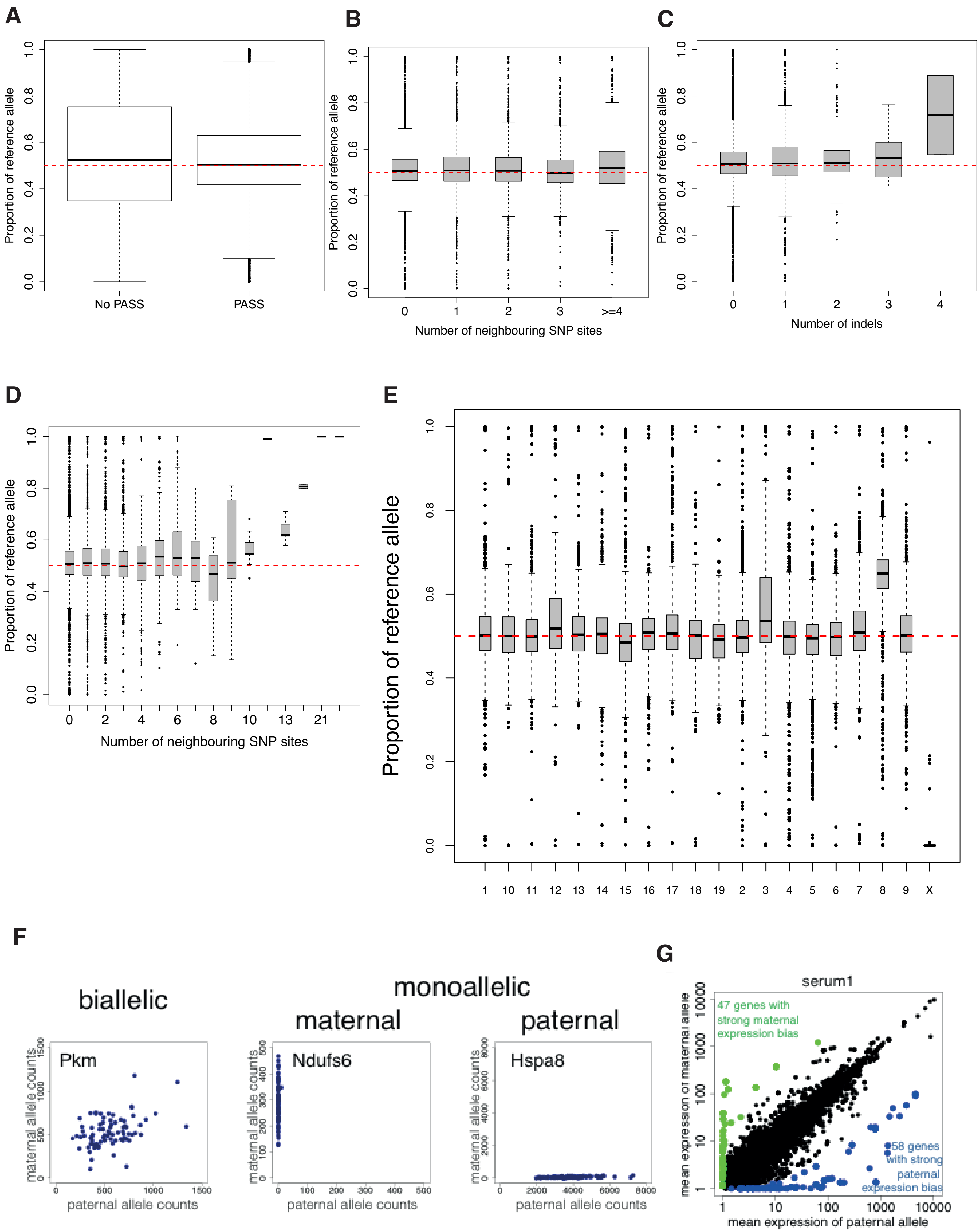

A
Proportion of reference allele
No PASS
PASS
B
Proportion of reference allele
Number of neighbouring SNP sites
C
Proportion of reference allele
Number of indels
D
Proportion of reference allele
Number of neighbouring SNP sites
E
Proportion of reference allele
F
biallelic
monoallelic
maternal
paternal
Pkm
Ndufs6
Hspa8
maternal allele counts
paternal allele counts
G
serum1
47 genes with strong maternal expression bias
58 genes with strong paternal expression bias
mean expression of maternal allele
mean expression of paternal allele

Figure S1. Quality control and mapping statistics, related to Figure 1

(A-B) Measures taken for quality control are (1) the number of reads mapping to exons being above 0.5 million, and (2) the proportion of reads mapped to mitochondrial genes being below 10%. Red dashed lines represent the thresholds we used. Red cells represent double cells, no cells or debris upon examination of the C1 chip. Cells cultured in 2i 1 are in panel A and serum 1 cells in B. (C) Correlation of gene expression levels in single cells. Expression levels correlate with each other as shown representatively for cells index 86, 91, 92 and 93 (serum 1). We removed cell 85, as it is substantially different than any other cell, suggesting failure of the experimental protocol. (D-E) Comparison of gene expression levels between bulk and single cells. Scatter plot between expression level in bulk experiment and mean of gene expression from single cells in each condition: 2i (D), serum1 (E). (F-H) Mapping statistics for each cell for each sample. GSNAP was used for mapping, and HTSeq for generating read count tables. The average percentage of reads mapping to Ensembl exons (green), to the genome outside Ensembl annotated regions (blue) and unmapped reads (red) for each of nine experiments. (I) Morphology and karyotype of G4 mES cell line. Light microscopy photograph depicting morphology of cells grown in three conditions. (J) Karyotyping result of the G4 (C57BL/6Ncr x 129S6/SvEvTac) mouse hybrid embryonic stem cells, showing no chromosomal aberrations. (K) Western blot of phosphorylation state of Erk1/2 – samples from left to right: 2i, a2i.

Figure S2. Gene expression noise measures, related to Figure 2

(A-C) Comparison of global gene expression across three culture conditions. Gene expression (A), squared coefficient of variation (B) and DM (C) distribution in all conditions. Gene expression is quantified in terms of counts normalised by reads per million. (D-E) Comparison of DM of all expressed genes among conditions processed in the same batch not including 2C-like cells. (E-F) DM (y-axis) is not correlated with gene length (E, Spearman's rho=0.0206) or mean normalized read counts (F, Spearman's rho=0.0200).

Figure S3. Gene expression noise comparison, related to Figure 2

(A-B) Comparison of gene expression noise for GO categories between 2i and a2i (A), or between serum and a2i (B). See the legend of Figure 6 for details. (C) Comparison of gene expression noise

for GO categories between 2i and a2i including 2C-like cells into 2i population (D) Specific functional categories of genes have a higher or lower level of noise in 2i1, and serum2. In the scatter plot, each point represents a GO term and some GO terms are determined to be significant (higher or lower noise than expected by chance) if a *P*-value from gene set enrichment analysis using DM is lower than 10^{-4}. See main text for details. (E-G) Noise comparison between 2i and serum data published in Grun et al., 2014. Scatterplots showing squared CV (E), Fano factor (F) and DM (G) for each gene between two conditions.

Figure S4. Differentiating mESC cells from serum culture overlap on PCA with differentiating NPC cells, related to Figure 3

mESC and NPC differentiating cells projected on the first two principal components. All genes with mean normalized counts larger than 50 were considered and PCA was performed on the Spearman's rank correlation matrix between cells.

Figure S5. Expression of key pluripotency factors in bulk mRNA sequencing data, related to Figure 5

Barplots showing size factor normalized counts for 64 genes involved in pluripotency

Figure S6. Comparison between 2i and a2i grown cells, related to Figure 5

(A) Venn diagrams showing number of differentially expressed genes between G1/S stage and G2/M stage 2i and a2i cells, KEGG pathway analysis (done using DAVID http://david.abcc.ncifcrf.gov/). (B) Comparison of mRNA content in cells using ratio of reads mapping to ERCCs (constant number of molecules spiked in in three conditions) to all exon mapped reads. (C) Barplots showing sign(log(fold change)) * –log(p-value) for differential expression of pluripotency (left) and differentiation genes. The genes that are downregulated have negative numbers, and genes that are upregulated positive. Red line indicates p-value 0.05.

Figure S7. Quality control for SNPs and, related to Figure 1

(A) SNPs not annotated as "PASS" in the FILTER column of the VCF file are in general biased toward the reference allele in the bulk data of the serum condition. (B-D) The number of

neighbouring SNP sites (B-C) and indels (D) is positively correlated with the proportion of the reference allele. (E) SNPs on chromosome 3, 8, and 12 are biased toward the reference allele. See Supplemental Methods for details. (F) Examples of genes from three gene expression categories: (1) biallelic, (2) monoallelic maternal and (3) monoallelic paternal. (G) Global overview of mean gene expression of both alleles for each gene in serum. Expression levels shown are numbers of reads mapping to the longest transcript of a gene. Genes identified as monoallelic maternal are highlighted in green and monoallelic paternal genes in blue.

Table S1. Enriched Gene Ontology categories in each condition, related to Figure 4.
Results of Gene Ontology analysis of differentially expressed genes in three comparisons, serum with 2i, 2i with a2i and serum with a2i.

Table S2. Monoallelic genes, related to Figure 1.
List of genes that exhibit monoallelic expression in all culture conditions

Table S3. gRNA sequences, related to Figure 7.
Primer sequences used for cloning into gRNA vector

Extended Experimental Procedures

Bulk RNA sequencing

Bulk mRNA sequencing libraries were prepared and sequenced using the Wellcome Trust Sanger Institute sample preparation pipeline with Illumina's TruSeq RNA Sample Preparation v2 kit. RNA was extracted from 1-2 million cells using the Qiagen RNA Purification Kit on a QiaCube robot. The quality of the RNA sample was checked using gel electrophoresis. For library preparation, poly-A RNA was purified from total RNA using oligo-dT magnetic pull-down. Subsequently, mRNA was fragmented using metal-ion catalyzed hydrolysis. The cDNA was synthesized using random hexamer priming, and end repair was performed to obtain blunt ends. A-tailing was done to enable subsequent ligation of Illumina paired-end sequencing adapters, and samples were multiplexed at this stage. The resulting library was amplified using 10 cycles of PCR, substituting the Kapa Hifi polymerase for the polymerase in the Illumina TruSeq kit. Samples were diluted to 4nM, and 100bp paired end sequencing carried out on an Illumina HiSeq2000. Sequencing Quality Control was performed by the Sanger sequencing facility.

Gene-level alignment of reads

For each cell, paired-end reads were aligned to the *Mus musculus* genome (GRCm38) using GSNAP (version gmap-2014-05-15_v2) with default options (Wu and Nacu, 2010). To detect splice junctions in reads, we used a set of known splice sites from the GTF file for GRCm38 provided by Ensembl (release 73). Only reads uniquely mapped to the genome were counted for each gene using htseq-count and the same GTF file (Anders et al., 2014).

Allele-level alignment of reads

To count reads at each SNP between C57BL/6Ncr and 129S6/SvEvTac, we constructed a set of SNPs common to all the 129 strains by taking an intersection of SNPs present in the three 129 strains (129S1/SvImJ, 129S5/SvEvBrd and 129P2/OlaHsd) that are available in the Sanger mouse genomes project (Keane et al., 2011). We also confirmed that the resulting 5,038,206 SNPs do not overlap with SNPs in C57BL/6NJ. The genome of the 129 strain was constructed by computationally mutating the SNP positions between 129 and C57BL/6J. The paired-end reads were mapped to both the reference and mutated genomes using GSNAP (version gmap-2014-05-15_v2) with default options (Wu and Nacu, 2010). Reads mapped to multiple locations were excluded. We extracted 120,036 exonic SNPs using the GTF file for GRCm38 provided by Ensembl (release 73) and found that a total of 31,695 transcripts have at least one exonic SNP. We counted reads for each exonic SNP using mpileup of samtools (version 0.1.19) (Li et al., 2009).

Quality control on SNPs

The 120,036 exonic SNPs were further examined to avoid bias in measures of allele-specific expression. First, we found that SNPs not annotated as “PASS” in the FILTER column of the VCF file provided by the Mouse Genomes Project (version 3) were biased toward the reference allele in the bulk data of serum (hereafter we will use the same data to test the quality of SNPs), suggesting SNPs that do not pass all filter criteria for a genotype call are unreliable. Second, the extent of unequal allelic abundance increased with the number of neighbouring SNP sites (i.e. the number of other SNP sites located within the SNP-flanking region, ± read length), which is consistent with a previous study showing the impact of the density of neighbouring SNP sites on the allelic abundance (Stevenson et al., 2013). Third,

increasing the number of insertions or deletions (indels) between 129 and C57BL/6J within the SNP-flanking region impaired the measures of allelic abundance, suggesting that the presence of indels can prevent the alignment of reads from 129 alleles to the mutated genome. Finally, SNPs on chromosomes 3, 8, and 12 were biased toward the reference allele. To exclude SNPs affected by the systematic bias toward the reference allele, we used the following criteria:

1) SNP quality annotation: only SNPs annotated as "PASS" were considered.

2) Density of neighbouring SNP sites: SNPs with the number of neighbouring SNP sites larger than or equal to 4 were removed.

3) Indels: SNPs with at least one indel nearby were removed.

4) Chromosome: SNPs on chromosome 3, 8, 12, and X (since the mouse is male) were removed.

In total, we have 66,291 exonic SNPs and 25,104 transcripts with at least one such SNP. As a quality check for the final exonic SNPs, we investigated the proportion of reads mapped to the maternal alleles of the X chromosome and found that 97.6-98.9% of reads mapped to SNPs on the X chromosome (i.e. that passed the first three criteria above) were correctly assigned to the maternal allele (129S6/SvEvTac) in the bulk data of the three culture conditions.

Identification of monoallelic genes

To identify genes with monoallelic expression, we computed the following three quantities for each transcript with at least one exonic SNP: 1) Proportion of paternal allele in the bulk

(αb), 2) P-value of binomial exact test in the bulk (P), where the null hypothesis is that the two alleles are equally expressed in the bulk, 3) Average proportion of paternal allele in the single cells (αs). If a transcript has multiple SNPs, we summed the read counts of these SNPs. A transcript is called monoallelic if ($\alpha b<0.05$ or $\alpha b>0.95$) and $P\leq0.01$ and ($\alpha s<0.05$ or $\alpha s>0.95$).

Expression of maternal *versus* paternal alleles defines monoallelic expression patterns.

In all analyses so far, we consider expression of a gene as the sum of expression from the maternal and paternal alleles. However, the SNPs between the two parental strains of our hybrid mESCs allow us to map reads to either the maternal or paternal allele for many genes, and quantify allele specific expression (Methods, Figure S7). To avoid potential confounding factors we karyotyped the cells and observed no abnormalities (Figure S3B). There are 13,642 genes with at least one SNP. After filtering lowly expressed genes and genes with uncertain SNPs, we were left with an average of 4,201 genes per condition for which we can distinguish maternal and paternal expression.

The previous literature on allele-specific expression of genes in mESCs contains various results, some of which appear to be contradictory. RNA-FISH for *Nanog* in 2i medium showed that *Nanog* expression is biallelic in most cells, while its expression in serum was reported to be lower and mostly monoallelic. Interestingly, other genes that have similar expression profiles to *Nanog* in our experiments, such as *Rex1*, were observed to be biallelically expressed according to RNA-FISH (Miyanari and Torres-Padilla, 2012). At the protein level, reporter constructs suggest that both alleles of *Nanog* are expressed. It has been pointed out that burst-like transcription rather than the traditional concept of monoallelic expression may be the origin of expression of one allele per cell (Faddah et al., 2013; Filipczyk et al., 2013).

Monoallelic expression is defined as all cells expressing only one allele. We found 155 monoallelically-expressed genes (91 paternal and 63 maternal)(Full list in Table S2). This corresponds to only 2% of the genes in our data set (Figure 7). 53 genes are consistently monoallelically expressed in all conditions. Monoallelic expression can be due to imprinting, or it can be a random process, mediated by a different, unknown mechanism, including burst-like kinetics of transcription.

We do not observe big differences in the number of monoallelic genes identified between conditions. There are 96 monoallelic genes in 2i1, 105 in serum1, 93 in serum2 and 81 in a2i1. We found only one instance when a transcript was identified as monoallelic in one condition, and biallelic in another. Some genes that are monoallelically expressed in one condition have low expression level in the other and do not pass through our expression level filter, so are not classified as either monoallelic or biallelic. Based on these results, monoallelic expression seems to be independent of the culture condition.

Quality control of cells

To exclude poor quality libraries from downstream analysis, we first removed cells that correspond to empty capture sites, capture sites with multiple cells, or capture sites containing cell debris on the C1 chip by visually inspecting them under microscope. Second, it has been known that some cells suffer from cell rupture during the process of microfluidic cell capture (Islam et al., 2014). To identify these abnormal cells, we calculated two quantities for each cell: the number of reads mapped to exons, and the proportion of reads (of all reads mapped to exons) mapped to 37 genes on the mitochondrial chromosome. We identified two populations of cells in serum1 in terms of the above two quantities and most of

the cells corresponding to empty cells or cell debris are in one of the two populations. Based on this, we set the following criteria to remove abnormal cells:

1) Cells that have fewer than 500,000 reads mapped to exons

2) Cells that have greater than 10% reads mapped to mitochondrial genes

We also validated the above criteria using the cells in 2i1, and found that 7 of 9 cells (empty or cell debris identified by visual inspection) were not accepted (Figure S1AB). Finally, we compared the normalised read counts of genes between cells in the same condition, and found that in one cell (cell “85” in the first replicate of serum) there was a problem in library preparation and many genes were abnormally amplified (Figure S1C). We removed the cell from further analysis. In summary, we have the following number of cells for the analysis: 81, 90 and 79 for serum replicates, 82, 59,72 and 82 for 2i replicates, 93 and 66 for a2i replicates, where the total number of cells across conditions is 704.

Adjusting for batch effects

To remove technical variation across multiple batches, we applied location and scale adjustments to the normalized read counts by using the ComBat function of the sva package of R with default options (Johnson et al., 2007). We first log10-transformed the normalized read counts (after removing lowly expressed genes whose mean normalised read counts are less than 10) and after adding a pseudo count of 1. Second, we adjusted for batch effects using ComBat with the known batch covariate and sample conditions. Finally, we re-transformed the batch-adjusted expression values x back to the original scale (10^x-1). If the re-transformed values are less than 0 or the original read counts are 0, we set the re-transformed values to 0.

Differential expression analysis

Differentially expressed genes were identified from bulk data and single cell data using the DESeq package. Genes that differed in expression by two fold and with a multiple testing adjusted p-value was < 0.05 were considered differentially expressed. For single cell differential expression analysis each cell was used as a replicate of the condition it came from and genes that had mean expression below 50 (DESeq size factor normalised) counts were removed.

Calculating DM as a measure of noise

To account for the confounding effects of gene length and mean expression level on the CV, we computed the DM values for each gene using rolling medians of the squared CV. First, we computed gene lengths by taking the union of all exons within a gene based on the Ensembl annotation. Here we excluded all exons annotated as "retained_intron". We also removed lowly expressed genes whose mean normalised read counts (reads per million) are less than 10, since we cannot distinguish biological noise from technical noise for these genes. Second, we computed rolling medians from the scatter plot between the mean normalised read counts and the squared CV values, where the x- and y-axis are log10 transformed. Third, we calculated the mean-corrected residual of the squared CV of gene i to its corresponding rolling median $f(i)$ such that

$$r(i) = \log_{10} CV(i)^2 - f(i).$$

Finally, to correct for the effect of gene length on the mean corrected residual, we calculated the difference between the mean corrected residual of the squared CV of gene i and its expected residual by using the following formula

$$DM(i) = r(i) - g(i),$$

where $g(i)$ is the rolling median of gene i from the scatter plot between $r(i)$ and log10 transformed gene lengths. To compute the rolling medians, we used the rollapply function of the zoo package of R (Zeileis and Grothendieck, 2005) and the following parameters: the number of genes in the window is 50 and the number of overlapping genes between adjacent windows is 25. This relative noise measure, which is referred to as DM, does not depend on either gene expression levels or gene lengths (Spearman's ρ=0.0200 for gene expression levels and ρ=0.0206 for gene length in the serum condition).

Analyzing the published single cell RNA-seq data of Grun *et al.* 2014

We performed noise analysis using data published previously (Grun et al., 2014) and validated our observation that there is no significant difference in noise levels between cells grown in 2i and serum. We observed that development and differentiation related GO categories were noisier in serum, while cell cycle related ones were noisier in 2i.

The expression levels of 10,809 genes that are expressed in both conditions are significantly higher in 2i than in the serum condition ($P<2.2\times10^{-16}$ by the Wilcoxon signed-rank test), while the estimated biological variance differs significantly in the same direction ($P<2.2\times10^{-16}$). This leads to a significant difference in the distribution of CV values between conditions ($P<2.2\times10^{-16}$, higher in serum, Figure S8A) but to a significant difference in the distribution of Fano factors in the opposite direction ($P<2.2\times10^{-16}$, higher in 2i, Figure S8B). To properly account for the confounding effect of expression level on noise measures, we used the abundance-corrected noise measure (DM). We observed no

significant difference of DM values between the two culture conditions (P=0.8740, Figure S8C). Since we cannot conclude that the expression of genes across the whole dynamic range of expression levels is more heterogeneous in the serum condition than in 2i, we asked whether noise levels in specific functional categories differ between the two culture conditions. We found that 92 Gene Ontology terms (out of 17,849) show a significant difference in their DM values (P<0.01 by the paired t-test). Notably, genes that play a role in mitosis have higher levels of noise in 2i compared to serum. In contrast, genes involved in development are more heterogeneous in serum than in 2i.

Testing the absolute level of cell-to-cell variation of a functional category within a culture condition

To test whether genes belonging to a defined functional category have a high or low level of expression heterogeneity within a culture condition, we performed gene set enrichment analysis using the Piano package of Bioconductor (Varemo et al., 2013). We used the DM values for gene-level statistics and calculated the mean DM values as a gene-set statistic for each GO term. The associations between Ensembl gene IDs and GO terms were obtained from the biomaRt package of Bioconductor (Kasprzyk, 2011). Since gene set enrichment analysis tends to bias towards large or small categories in terms of their number of genes, we considered only gene sets with between 3 and 2,000 genes. The *P*-value for each GO term was then computed by randomly taking a set of genes of the same size as in the GO term, and by repeating this 10,000 times.

Testing the relative difference in expression heterogeneity of a functional category across culture conditions

To explore further the difference of the three culture conditions in terms of gene expression noise, we compared two sets of DM values for each GO term between two culture conditions using the two-sided paired t-test. We only considered GO terms with at least 2 genes having DM values. The associations between GO terms and their offspring terms were obtained from the GO.db annotation package of Bioconductor

(http://www.bioconductor.org/packages/release/data/annotation/html/GO.db.html).

Doubling time estimation of mouse embryonic stem cells in different conditions

Fifty thousands G4 mouse ES cells were plated in single wells on gelatinized 6-well plates, and maintained in the three culture conditions of interest (total 12 wells for each culture condition): serum-containing media, standard 2i media and alternative 2i media. Three wells were harvested and quantified on a hemocytometer every 24 hours for 4 days to estimate the doubling time of mouse ES cells in each condition.

Western Blotting

Cells were cultured in standard 2i media: N2B27 basal media (NDiff 227, StemCells), 1 μM PD0325901 (Stemgent), 3 μM CHIR99021 (Stemgent) and alternative 2i media: N2B27 basal media (NDiff 227, StemCells), 1 μM CGP77675 (Sigma), 3 μM CHIR99021 (Stemgent). Samples were prepared from 5mln of cells, lysed, and diluted in to final volume of 200ul 1x Laemmli buffer. 10ul of each sample was run on 10% Mini-PROTEAN® TGX™ Precast Protein Gel and blotted to nitrocellulose membrane. The efficiency of blotting was inspected using Ponceau stain. Blots were blocked with 5%BSA in TBS and washed with 0.05% Tween20 TBS. Primary antibodies used were p44/42 MAPK (Erk1/2) #9102 and Phospho-p44/42 MAPK (Erk1/2) (Thr202/Tyr204) #9101 from Cell Signalling. HRP conjugated

secondary antibodies were used and Thermo Scientific Pierce ECL Western Blotting Substrates were used for detection.

References

Anders, S., Pyl, P.T., and Huber, W. (2014). HTSeq – A Python framework to work with high-throughput sequencing data. bioRxiv preprint.
Faddah, D.A., Wang, H., Cheng, A.W., Katz, Y., Buganim, Y., and Jaenisch, R. (2013). Single-cell analysis reveals that expression of nanog is biallelic and equally variable as that of other pluripotency factors in mouse ESCs. Cell stem cell *13*, 23-29.
Filipczyk, A., Gkatzis, K., Fu, J., Hoppe, P.S., Lickert, H., Anastassiadis, K., and Schroeder, T. (2013). Biallelic expression of nanog protein in mouse embryonic stem cells. Cell stem cell *13*, 12-13.
Grun, D., Kester, L., and van Oudenaarden, A. (2014). Validation of noise models for single-cell transcriptomics. Nat Methods *11*, 637-640.
Islam, S., Zeisel, A., Joost, S., La Manno, G., Zajac, P., Kasper, M., Lönnerberg, P., and Linnarsson, S. (2014). Quantitative single-cell rna-seq with unique molecular identifiers. Nat Methods *11*, 163.
Johnson, W.E., Li, C., and Rabinovic, A. (2007). Adjusting batch effects in microarray expression data using empirical Bayes methods. Biostatistics *8*, 118-127.
Kasprzyk, A. (2011). BioMart: driving a paradigm change in biological data management. Database *2011*.
Keane, T.M., Goodstadt, L., Danecek, P., White, M.A., Wong, K., Yalcin, B., Heger, A., Agam, A., Slater, G., Goodson, M.*, et al.* (2011). Mouse genomic variation and its effect on phenotypes and gene regulation. Nature *477*, 289-294.
Li, H., Handsaker, B., Wysoker, A., Fennell, T., Ruan, J., Homer, N., Marth, G., Abecasis, G., Durbin, R., and Genome Project Data Processing, S. (2009). The Sequence Alignment/Map format and SAMtools. Bioinformatics *25*, 2078-2079.
Miyanari, Y., and Torres-Padilla, M.E. (2012). Control of ground-state pluripotency by allelic regulation of Nanog. Nature *483*, 470-473.
Stevenson, K.R., Coolon, J.D., and Patricia J Wittkopp, P.J. (2013). Sources of bias in measures of allele-specific expression derived from RNA-seq data aligned to a single reference genome. BMC genomics *14*.
Varemo, L., Nielsen, J., and Nookaew, I. (2013). Enriching the gene set analysis of genome-wide data by incorporating directionality of gene expression and combining statistical hypotheses and methods. Nucleic acids research *41*, 4378-4391.
Wu, T.D., and Nacu, S. (2010). Fast and SNP-tolerant detection of complex variants and splicing in short reads. Bioinformatics *26*, 873-881.
Zeileis, A., and Grothendieck, G. (2005). zoo: S3 Infrastructure for Regular and Irregular Time Series. J Stat Soft *14*.
