# Supplementary material for: Single Cell RNA-Sequencing of Pluripotent States Unlocks Modular Transcriptional Variation
Source: Cell Stem Cell. 2015 Oct 1;17(4):471–85. doi: 10.1016/j.stem.2015.09.011 (PMC4595712; doi:10.1016/j.stem.2015.09.011)
Supplement: Document S2. Article plus Supplemental Information [file mmc5.pdf]

Resource

Cell Stem Cell

Single Cell RNA-Sequencing of Pluripotent States Unlocks Modular Transcriptional Variation

Graphical Abstract

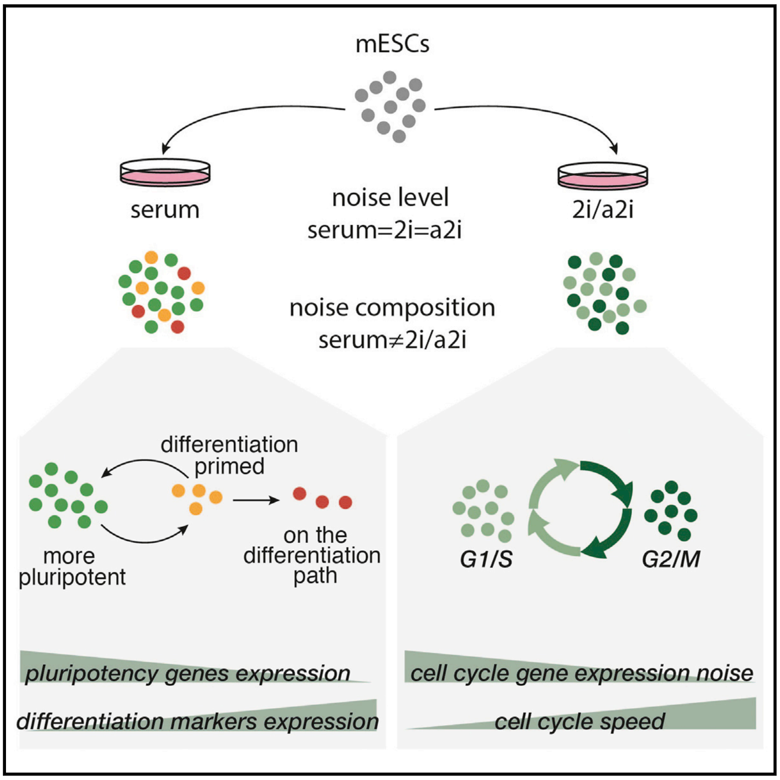

Authors

Aleksandra A. Kolodziejczyk,
Jong Kyoung Kim,
Jason C.H. Tsang, ..., Pentao Liu,
John C. Marioni, Sarah A. Teichmann

Correspondence

marioni@ebi.ac.uk (J.C.M.),
st9@sanger.ac.uk (S.A.T.)

In Brief

Teichmann, Marioni, and colleagues report full-transcript single cell RNA-sequencing of mESCs cultured in three different conditions: serum, 2i, and the alternative ground state a2i. They find that overall levels of intercellular heterogeneity are comparable across the three conditions, but different sets of genes are variably expressed.

Highlights

- mESCs grown in three different conditions show distinct transcriptomes
- Global intercellular variation is at similar levels in all three conditions
- 2i and a2i conditions maintain pluripotency with metabolic variation
- Correlation analysis identifies additional pluripotency network genes

Kolodziejczyk et al., 2015, Cell Stem Cell *17*, 471–485
October 1, 2015 ©2015 The Authors
http://dx.doi.org/10.1016/j.stem.2015.09.011

Cell Stem Cell
Resource

Single Cell RNA-Sequencing of Pluripotent States Unlocks Modular Transcriptional Variation

Aleksandra A. Kolodziejczyk,[1,2,5] Jong Kyoung Kim,[1,5] Jason C.H. Tsang,[2] Tomislav Ilicic,[1,2] Johan Henriksson,[1] Kedar N. Natarajan,[1,2] Alex C. Tuck,[1,3] Xuefei Gao,[2] Marc Bühler,[3] Pentao Liu,[2] John C. Marioni,[1,2,4,*] and Sarah A. Teichmann[1,2,*]

[1]European Molecular Biology Laboratory, European Bioinformatics Institute (EMBL-EBI), Wellcome Trust Genome Campus, Hinxton, Cambridge CB10 1SD, UK
[2]Wellcome Trust Sanger Institute, Wellcome Trust Genome Campus, Hinxton, Cambridge CB10 1SA, UK
[3]Friedrich Miescher Institute for Biomedical Research, Maulbeerstrasse 66, 4058 Basel, Switzerland
[4]University of Cambridge, Cancer Research UK Cambridge Institute, Robinson Way, Cambridge, CB2 0RE, UK
[5]Co-first author
*Correspondence: marioni@ebi.ac.uk (J.C.M.), st9@sanger.ac.uk (S.A.T.)
http://dx.doi.org/10.1016/j.stem.2015.09.011
This is an open access article under the CC BY license (http://creativecommons.org/licenses/by/4.0/).

SUMMARY

Embryonic stem cell (ESC) culture conditions are important for maintaining long-term self-renewal, and they influence cellular pluripotency state. Here, we report single cell RNA-sequencing of mESCs cultured in three different conditions: serum, 2i, and the alternative ground state a2i. We find that the cellular transcriptomes of cells grown in these conditions are distinct, with 2i being the most similar to blastocyst cells and including a subpopulation resembling the two-cell embryo state. Overall levels of intercellular gene expression heterogeneity are comparable across the three conditions. However, this masks variable expression of pluripotency genes in serum cells and homogeneous expression in 2i and a2i cells. Additionally, genes related to the cell cycle are more variably expressed in the 2i and a2i conditions. Mining of our dataset for correlations in gene expression allowed us to identify additional components of the pluripotency network, including Ptma and Zfp640, illustrating its value as a resource for future discovery.

INTRODUCTION

Mouse embryonic stem cells (mESCs) are derived ex vivo from the inner cell mass of the developing blastocyst. They are characterized by their capacity for in vitro self-renewal and the preservation of developmental pluripotency to reconstitute embryonic lineages (Bradley et al., 1984; Evans and Kaufman, 1981; Martin, 1981). Genetic studies have established the role of *Oct4* (Nichols et al., 1998), *Sox2* (Avilion et al., 2003), *Nanog* (Chambers et al., 2003; Mitsui et al., 2003) and *Esrrb* (Festuccia et al., 2012) as the signature core factors in the pluripotency transcriptional network of mESCs (Chen et al., 2008; Loh et al., 2006; Marson et al., 2008).

Maintenance of self-renewal in vitro is dependent on the interplay between extracellular cues and the pluripotency network. This is conventionally achieved through combinatorial stimulation of the JAK-STAT pathway and ID proteins by cytokine leukemia inhibitory factor (LIF) and fetal calf serum (serum)/bone morphogenetic proteins (BMPs), respectively (Smith et al., 1988; Williams et al., 1988; Ying et al., 2003). mESCs propagated in serum/LIF conditions remain exposed to differentiation cues from autocrine fibroblast growth factor 4 (FGF4) or LIF through the RAS-ERK signaling pathway (Burdon et al., 1999; Kunath et al., 2007; Niwa et al., 2009; Ying et al., 2008), although genetic and chemical inhibition of the FGF-ERK pathway alone is able to prevent differentiation (Kunath et al., 2007). These findings led to the establishment of the concept of "ground state pluripotency," where differentiation cues are shielded, and the pluripotency network is intrinsically stable (Nichols and Smith, 2009; Ying et al., 2008).

With additional inhibition of glycogen synthase kinase 3 (GSK3), ground state mESCs can be robustly maintained in vitro in the chemically defined 2i condition. Dual inhibition of GSK3 and ERK promotes self-renewal by alleviating TCF3-mediated repression, activating *Esrrb* expression, reducing degradation of KLF2 (Martello et al., 2012; Wray et al., 2011; Yeo et al., 2014), and inducing *Tfcp2l1* in concert with LIF (Ye et al., 2013). Substituting ERK kinase inhibition with inhibition of members of the SRC tyrosine kinase family can enable maintenance of an alternative ground state, alternative 2i, or a2i (Li et al., 2011; Shimizu et al., 2012). As SRC tyrosine kinase inhibition only partially reduces phosphorylation of ERK kinase (Shimizu et al., 2012), its effect on differentiation is not limited to convergent upstream inhibition of the FGF-ERK pathway. It has instead been suggested to block the epithelial-mesenchymal transition downstream of both the calcineurin-NFAT and the FGF-ERK pathways (Li et al., 2011) and stop differentiation by mechanical stress through an ERK-independent mechanism (Shimizu et al., 2012). Thus, the self-renewing pluripotent state of mESCs can be achieved through manipulation of key signaling pathways in vitro.

Despite sharing a common origin and defining properties, mESCs propagated under different culture conditions also differ (Ficz et al., 2013; Marks et al., 2012). For instance, serum/LIF-maintained mESCs are morphologically heterogeneous and

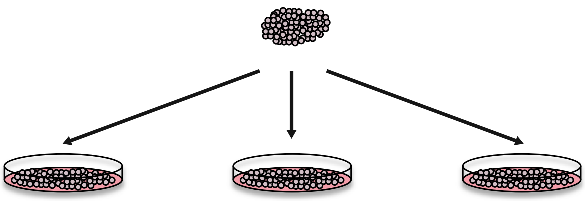

culture condition	serum	2i	alternative 2i (a2i)
components of medium	DMEM<br>15% fetal bovine serum<br>+ leukemia inhibitory factor (LIF)	N2B27 basal media<br>inhibitors of:<br>GSK3β (CHIR99021)<br>Mek1/2 (PD0325901)<br>+ LIF	N2B27 basal media<br>inhibitors of:<br>GSK3β (CHIR99021)<br>Src (CGP77675)<br>+ LIF
cell characteristics	more differentiation permissive<br>more heterogeneous	ground pluripotent state<br>more homogeneous	not well characterized
references	Pease et al., 1990<br>Xu et al., 2001	Ying et al., 2008<br>Li et al., 2008	Shimizu et al., 2012
number of cells	chip 1 - 81 cells<br>chip 2 - 90 cells<br>chip 3 - 79 cells	chip 1 - 82 cells<br>chip 2 - 59 cells<br>chip 3 - 72 cells<br>chip 4 - 82 cells	chip 1 - 93 cells<br>chip 2 - 66 cells

Figure 1. Experimental Scheme of Hybrid mESCs in Three Culture Conditions

Schematic of experimental setup and cell culture conditions used in our study.

show transcriptional fluctuation of certain pluripotency factors such as *Nanog* (Chambers et al., 2007; Kalmar et al., 2009), *Dppa3* (Hayashi et al., 2008), and *Rex1* (*Zfp42*) (Toyooka et al., 2008), unlike mESCs maintained in 2i conditions. These fluctuations have been proposed to represent a dynamic equilibrium between self-renewing and differentiation-poised states and thus be instrumental in regulating exit from pluripotency (Chang et al., 2008). However, others speculate that they arise through the use of fluorescent reporter systems and therefore are of unclear biological relevance (Chang et al., 2008; Faddah et al., 2013; Reynolds et al., 2012). The presence of transcriptionally heterogeneous subpopulations, prevalent bivalent chromatin domains, increased methylation content, and reduced RNA polymerase pausing compared to 2i mESCs has led to the notion that serum-maintained mESCs exist in a metastable pluripotent state (Marks et al., 2012), implying higher transcriptional cell-to-cell variation than the 2i state. Recently, a rare population of mESCs expressing markers of the two-cell stage of embryonic development was described (Macfarlan et al., 2012). These so-called 2C-like cells express the MERVL endogenous retrovirus and chimeric transcripts that arise via retroviral insertion in different places in the genome, and they are uniquely capable of differentiating into extraembryonic tissues. Our molecular understanding of the divergent pluripotent states, however, remains quite limited.

Single cell RNA-sequencing technology is increasingly used to deconstruct heterogeneous populations, lineage trajectories, and determinants of cell fate, questions that are central to the stem cell field (Etzrodt et al., 2014). Recently, Kumar et al. (2014) reported the single-cell transcriptome of serum/LIF-maintained mESCs and global transcriptome changes resulting from a range of chemical and genetic perturbations. Here, we performed single cell RNA-sequencing of mESCs cultured in serum/LIF, 2i/LIF, and the alternative ground state, a2i/LIF. This approach allowed us to compare the subpopulation structures and provide a deep characterization of cell-to-cell variation in gene expression levels across these three pluripotent states.

RESULTS

To examine features of gene expression heterogeneity across pluripotent states, we cultured an F1 hybrid (C57BL/6Ncr male x 129S6/SvEvTac female) mESC cell line (George et al., 2007) in three different conditions: (1) three replicates of serum + LIF, (2) four replicates of 2i + LIF, and (3) two replicates of a2i + LIF, which we will refer to as serum (serum1, serum2, and serum3), 2i (2i1, 2i2, 2i3, and 2i4) and a2i (a2i1 and a2i2) henceforth (Figure 1). In total, we collected 704 single-cell transcriptomes across these three conditions by using the Fluidigm C1 system and applying the SMARTer Kit to obtain cDNA and the Nextera XT Kit for Illumina library preparation.

After quality control analysis on each individual cell (Figures S1A–S1H), 250 serum cells, 295 2i cells, and 159 a2i cells remained. On average, we sequenced over 9 million reads per cell. Over 80% of reads mapped to the *Mus musculus* genome (GRCm38) and over 60% to exons (mapping overview in Figures S1G and S1H). We also performed standard bulk RNA-sequencing for each condition. As in previous studies, when we averaged gene expression levels across the single cells profiled in each condition, we observed that the mean expression levels recapitulated the bulk gene expression levels with a Spearman rank correlation coefficient of around 0.9 (Figures S1D and S1E).

Transcriptome-wide Cell-to-Cell Variation Is Similar across the Three Culture Conditions

An advantage of the single-cell approach is that we can study the distribution of expression levels across the population, thereby capturing cell-to-cell variability in gene expression (Figure 2A). To compare global levels of gene expression heterogeneity between the three different culture conditions, we used the coefficient of variation (CV) of normalized read counts (Figure S2). However, the CV of a gene depends strongly on its mean expression level and length, making it difficult to interpret differences between conditions. To account for the confounding factor of expression level, we therefore developed a measure of cell-to-cell variation by calculating the distance between the squared CV of each gene and a running median (Figures S2E and S2F). This is derived from the scatterplot of the mean normalized read counts versus the squared CV values, as in (Newman et al., 2006). We refer to this expression-level normalized measure of gene expression heterogeneity as distance to the median (DM) (refer to Supplemental Experimental Procedures for details).

Given the heterogeneous morphology of mESCs cultured in serum (Marks et al., 2012; Toyooka et al., 2008), as well as the

heterogeneous expression of pluripotency factors (Canham et al., 2010; Hayashi et al., 2008; Kalmar et al., 2009; Singh et al., 2007), it was surprising that transcriptome-wide DM values are not significantly different across the three culture conditions ($p = 0.625$ by the Freidman rank sum test) (Figures S2B–S2D).

This prompted us to ask whether the levels of heterogeneity for genes belonging to individual functional categories are also consistent between conditions. We first performed gene set enrichment analysis for each culture condition to test whether genes belonging to Gene Ontology (GO) terms are enriched among those genes with extreme DM values. We observed that genes involved in translation, ribosome, RNA binding, structural molecule activity, and mRNA processing have a lower level of gene expression heterogeneity for all conditions (Figure S3D). In contrast, genes involved in plasma membrane, metal ion binding, lysosome, and integral component of membrane exhibit higher variation than expected by chance in all three conditions ($p < 10^{-4}$). To gain more insight into how gene expression heterogeneity for functional categories differs between culture conditions, we compared the DM values of genes in pairs of culture conditions for each GO term (excluding 2i replicates containing 2C-like cells; for discussion of 2C-like cells, see below) (Figures 2B–2D, Figures S3A and S3B). We found that 712 GO terms (out of a total of 19,107 terms) exhibit a significant difference in levels of gene expression heterogeneity in at least one pairwise comparison ($p < 0.01$). For example, the expression of genes involved in "organ development" ($p = 3.3 \times 10^{-4}$) and "cell adhesion" ($p = 4.8 \times 10^{-4}$) is more heterogeneous in serum than in the inhibitory conditions (2i and a2i): these terms contain many pluripotency factors.

In contrast, genes involved in "cell cycle" ($p = 5.4 \times 10^{-3}$) and "nuclear division" ($p = 5.9 \times 10^{-6}$) have higher levels of gene expression heterogeneity in 2i compared to serum (Figures 2B–2D, Figures S3A and S3B). When we included 2i replicates containing 2C-like cells, we observed a similar trend (Figure S3C). When clustering cells based on cell-cycle genes only, we found that 2i cells separate into two groups: one with high expression of G2 and M genes and a second with lower expression of these genes (Figures 2E and 2F). Cells in serum and 2i also show different doubling kinetics with a rapid initial growth rate in 2i (24 hr). At the time of harvest, however (48 hr after plating), the doubling time of cells in 2i is 25 hr and in serum it is 11 hr, indicating that cells grown in 2i cycle more slowly, probably due to a longer G1 phase (refer to Supplemental Experimental Procedures).

As an independent validation, we performed the same analysis using data published previously (Grün et al., 2014). Consistent with our observations, global levels of gene expression heterogeneity between cells grown in 2i and in serum were comparable, while GO categories for development and differentiation were more heterogeneous in serum than in 2i, and cell-cycle genes were more heterogeneous in 2i than in serum (Figures S3E–S3G, Supplemental Experimental Procedures, Table S2).

Three Subpopulations Can Be Delineated in Serum-Grown mESCs

Genes with heterogeneous expression, especially those with clear bimodal expression (Figure 2A), may indicate the existence of underlying subpopulations. Indeed, hierarchical clustering of subsets of known pluripotency genes and differentiation markers reveals that serum-grown cells split into three distinct groups (Figure 3A). Similar to others, we found heterogeneous expression of *Nanog* (Faddah et al., 2013; Kalmar et al., 2009; MacArthur et al., 2012; Singh et al., 2007), *Esrrb* (van den Berg et al., 2008), and *Zfp42* (Toyooka et al., 2008) in serum, as well as heterogeneous expression of *Nr0b1* and *Utf1*.

One subpopulation consists of 39 cells (15%) that express higher levels of markers of differentiation, for example *Fos* or *Hes1*, and high levels of cytoskeletal genes such as keratins (*Krt8* and *Krt18*), actins (*Acta1* and *Acta2*), and annexins (*Anxa1*, *Anxa2*, and *Anxa3*). At the same time, these 39 cells have low levels or no expression of transcription factors involved in the maintenance of pluripotency (e.g., *Nanog*, *Sox2*, and *Oct4*) (Figures 3B and 3C), suggesting that these cells have exited pluripotency and committed to differentiation. A second group consists of 42 cells (17%) with somewhat lower expression levels of some pluripotency genes, such as *Dppa3* and *Nanog*, and some expression of differentiation genes, yet high expression of *Oct4* and *Sox2*. These cells may correspond to a previously described "differentiation permissive" set (Chambers et al., 2007; Islam et al., 2014; Kalmar et al., 2009). The largest group, which consists of 169 cells (68%), expresses the highest levels of pluripotency factors and exhibits very low expression of keratins or actins (Figures 3B and 3C).

We observe that the 39-cell and 42-cell populations, which have begun to move forward on the differentiation pathway, have heterogeneous expression of cell-cycle genes (Figure 3D). A shift in the distribution of the expression of G2/M genes, such as Cks2 or Cdc20, toward lower levels suggests that there are relatively more G1/S cells in these two groups as well. We inferred that more differentiated cells have a relatively longer G1 phase, as we sample more cells in G1 from this subpopulation relative to more pluripotent cells. This indicates that the 39-cell and 42-cell subsets that we identified proliferate more slowly than Nanog-high ground state pluripotent cells (Figure 3D). Moreover, we performed principal component analysis (PCA) of our data together with cells from an mESC-to-NPC (neural progenitor cell) differentiation time course (Bibel et al., 2007). We observed that cells belonging to the differentiating subpopulation overlap with cells that are differentiating toward NPCs (Figure S4). This strongly supports our earlier hypothesis that these cells are indeed progressing down a differentiation pathway (Figure 3A).

Ground State mESCs Cultured in Different Media Have Non-Overlapping Transcriptomes

Kalmar et al. (2009) suggested that mESCs grown in 2i are similar, or potentially identical, to the Nanog-high mESC subpopulation cultured in serum. To investigate whether ground state mESCs in serum (i.e. the cells we identified as most pluripotent within serum-only media) have a similar transcriptome to 2i or a2i mESCs, we clustered each population based on their global expression profiles. PCA (Figure 4A) demonstrates three separate clusters, revealing that ground state mESCs grown under different culture conditions in fact have distinct transcriptome identities. This is consistent with observations comparing bulk RNA-sequencing of Rex1-high (Zfp42-high) cells in serum and cells in 2i (Marks et al., 2012).

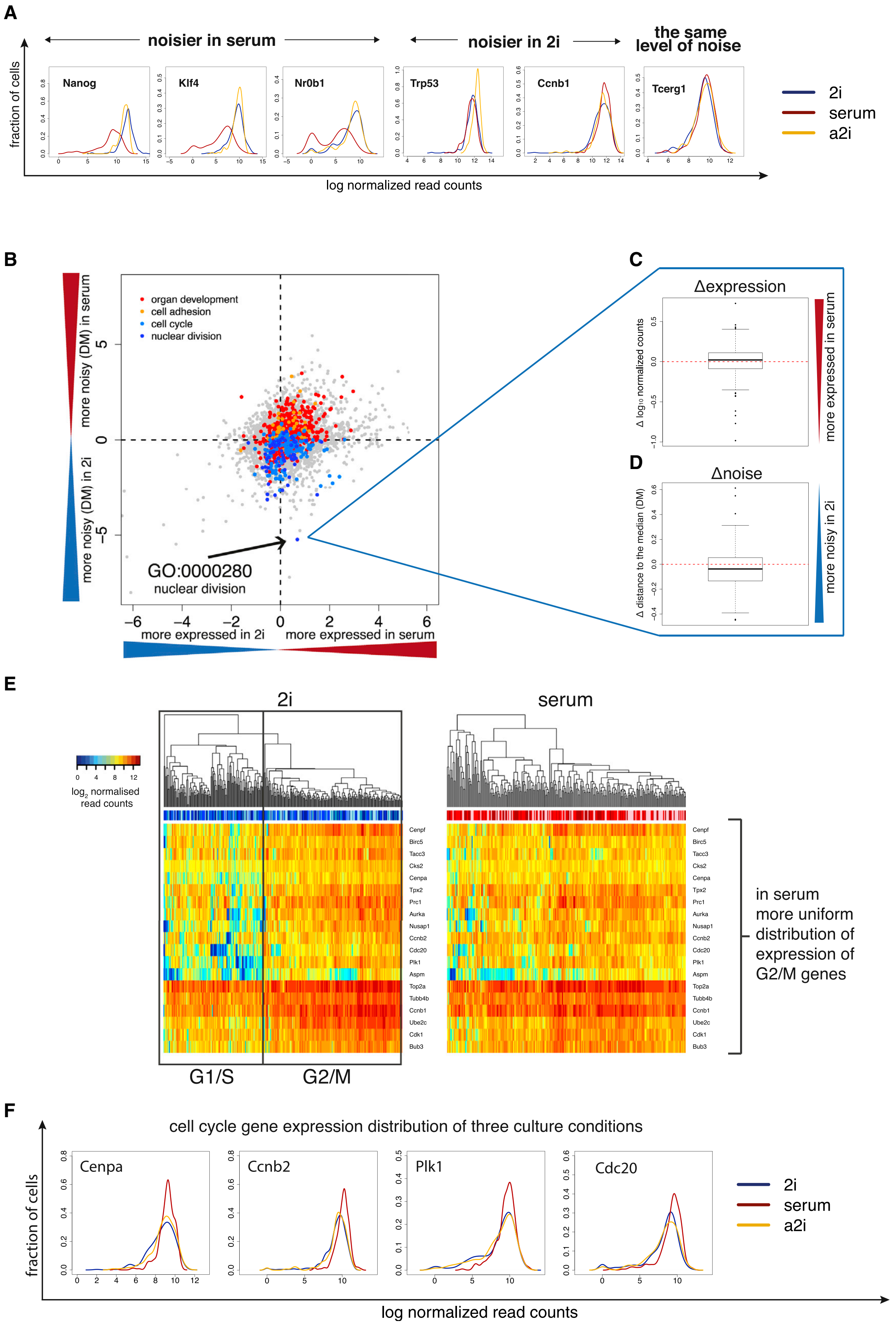

(legend on next page)

The Spearman correlation coefficient of mean gene expression levels between cells grown in the two inhibitory conditions is 0.95; between 2i and serum, it's 0.88; and between a2i and serum, it's 0.91. While these results suggest that 2i and a2i cells are more similar to each other than to serum-cultured cells, differences do exist between the two populations. Differences between 2i and a2i arise from the use of different inhibitors (Shimizu et al., 2012). Inhibition of Mek1/2 results in dephosphorylation of Erk1/2, while inhibition of Src does not have this effect, as we show by western blotting (Figure S1K).

To examine what differences in gene expression between the culture conditions explain the separation into distinct clusters, we performed GO enrichment analysis. We found that genes involved in development and differentiation, MAPK signaling, and basic metabolism are responsible for the separation (Figures 4B and 4C). To identify specific genes, we used DESeq, where each cell was considered a replicate of its culture condition, to test for significant differences in expression (Anders and Huber, 2010) as described in the Supplemental Experimental Procedures. There is a substantial amount of technical noise in single-cell data, so we considered only genes that are expressed on average above 50 normalized counts, as the technical bias is most pronounced for lowly expressed genes (Brennecke et al., 2013). This results in 4,587 differentially expressed genes between serum-grown cells and 2i-grown cells, 3,056 between serum and alternative 2i, and 2,061 genes between the two inhibitory conditions (the list of DE genes is available at http://www.ebi.ac.uk/teichmann-srv/espresso; Figure S5).

The two most enriched GO categories in genes differentially expressed between serum and 2i are in utero embryonic development (GO:0001701) and positive regulation of transcription from RNA polymerase II promoter (GO:0045944) (Table S1). Many of the transcription factors in the latter are key genes involved in pluripotency, such as *Nanog*, *Oct4*, *Klf4*, and *Sox2*. The differences between the two inhibitory conditions are smaller, and key terms are related to cell cycle, metabolism, and translation. Importantly, Oct4, Sox2, and Klf4 are not differentially expressed between 2i and a2i. Additionally, while Nanog is significantly differentially expressed, the expression level difference between 2i and a2i is smaller than between 2i and serum (log fold change = -0.71, adj $p < 10^{-6}$ and log fold change = 2.4, adj $p < 10^{-98}$, respectively). We observed the same pattern of differential gene expression in the bulk RNA-sequencing experiments (Table S1).

We hypothesized that differences between 2i and a2i, which are related to cell cycle and metabolism, may originate from different proportions of G1/S to G2/M phase cells in each condition. Indeed, using pre-defined cell cycle marker genes, we found that roughly 60% of cells in 2i are in G2/M and only 35% of cells are in G2/M in a2i. We therefore split the cells in each culture condition into G2/M and G1/S subgroups and compared G2/M cells from 2i with G2/M cells from a2i, and we did similarly for the G1/S subgroups. Subsequently, we considered the intersection of genes that were differentially expressed in each comparison and performed GO enrichment and KEGG pathway enrichment analyses. Overall, there are 97 genes with higher expression in 2i and 449 genes with higher expression in a2i. The genes that are upregulated in a2i are involved in RNA processing and transport, translation, and basic metabolism (Figure S6A).

The fact that, even after accounting for cell cycle, differentially expressed genes relate to basic cellular processes led us to explore whether cells cultured in a2i have more mRNA than cells cultured in 2i. To do this we exploited an external spike in molecules that we added to one batch of the cells (2i2, a2i2, and serum 3). The same number of molecules was added to each cell lysate, meaning that the ratio of all reads mapped to the spike ins to all reads mapped to exons can be considered as a proxy for cellular mRNA content (Ding et al., 2015; Stegle et al., 2015). Confirming the reliability of our approach, when we divided 2i and a2i cells into G1/S and G2/M subpopulations and compared their mRNA content, we found that cells in G2/M have significantly more mRNA than cells in G1/S (Figure S6B). Importantly, we observed that cells in 2i contain significantly fewer mRNA molecules than cells in serum and a2i (Wilcoxon test $p < 10^{-15}$ for both comparisons), which supports the differential expression of genes involved in basic cellular processes.

mESC Transcriptomes Are Similar, but Not Identical, to Those of Blastocyst Cells

It has been suggested that the pluripotent state of 2i cells resembles the cell state of early epiblast cells in the blastocyst (Boroviak et al., 2014; Nichols and Smith, 2009). The recent availability of single cell RNA-sequencing data from different stages of mouse embryonic development allowed us to assess the relationship of in vitro ESCs and in vivo blastomeres (Deng et al., 2014). Our cells were prepared with a very similar protocol, so we used PCA to overlay our data with the published embryonic time course. As expected, mESCs are most similar to the blastocyst stage cells from which they were derived, but they do not overlap (Figures 4D and 4E). The difference between in vivo

Figure 2. Global Cell-to-Cell Variation in Gene Expression

(A) Gene expression distributions of genes, which are noisier in 2i than serum, that have similar noise profiles in serum (red), 2i (blue), and a2i (yellow). Distributions of gene expression were smoothed using the kernel density estimation function in R with default parameters. Tcerg1 does not have significantly different expression profiles between culture conditions (two-sided KS test p value for 2i and a2i comparison is 0.82, and for 2i and serum, 0.16). By contrast, other genes, such as Ccnb1, are more heterogeneous in 2i ($p = 7 \times 10^{-4}$ by two-sided KS test between 2i and serum), while some, such as Nanog, Klf4, or Nr0b1, are more heterogeneous in serum ($p < 10^{-15}$ by two-sided KS test between 2i and serum for genes shown).

(B) Comparison of the levels of gene expression and noise for gene ontology (GO) categories between serum and 2i (excluding 2i replicates containing 2C-like cells). The logarithm (log10) of p values from two-sided paired t tests applied to mean normalized read counts (x axis) and DMs (y axis) was computed for each GO category and plotted against each other by multiplying the sign of the t statistic.

(C and D) Example of a GO category (GO:0000280, nuclear division) that is noisier in 2i (C) and is similarly expressed between the two conditions (D).

(E) Heatmaps showing the expression of cell-cycle-related genes in serum and 2i, with a distinct separation into G1/S versus G2/M cells in 2i, with less distinction between individual cells in serum.

(F) Gene expression profiles for key cell-cycle genes in all conditions show more heterogeneity in 2i.

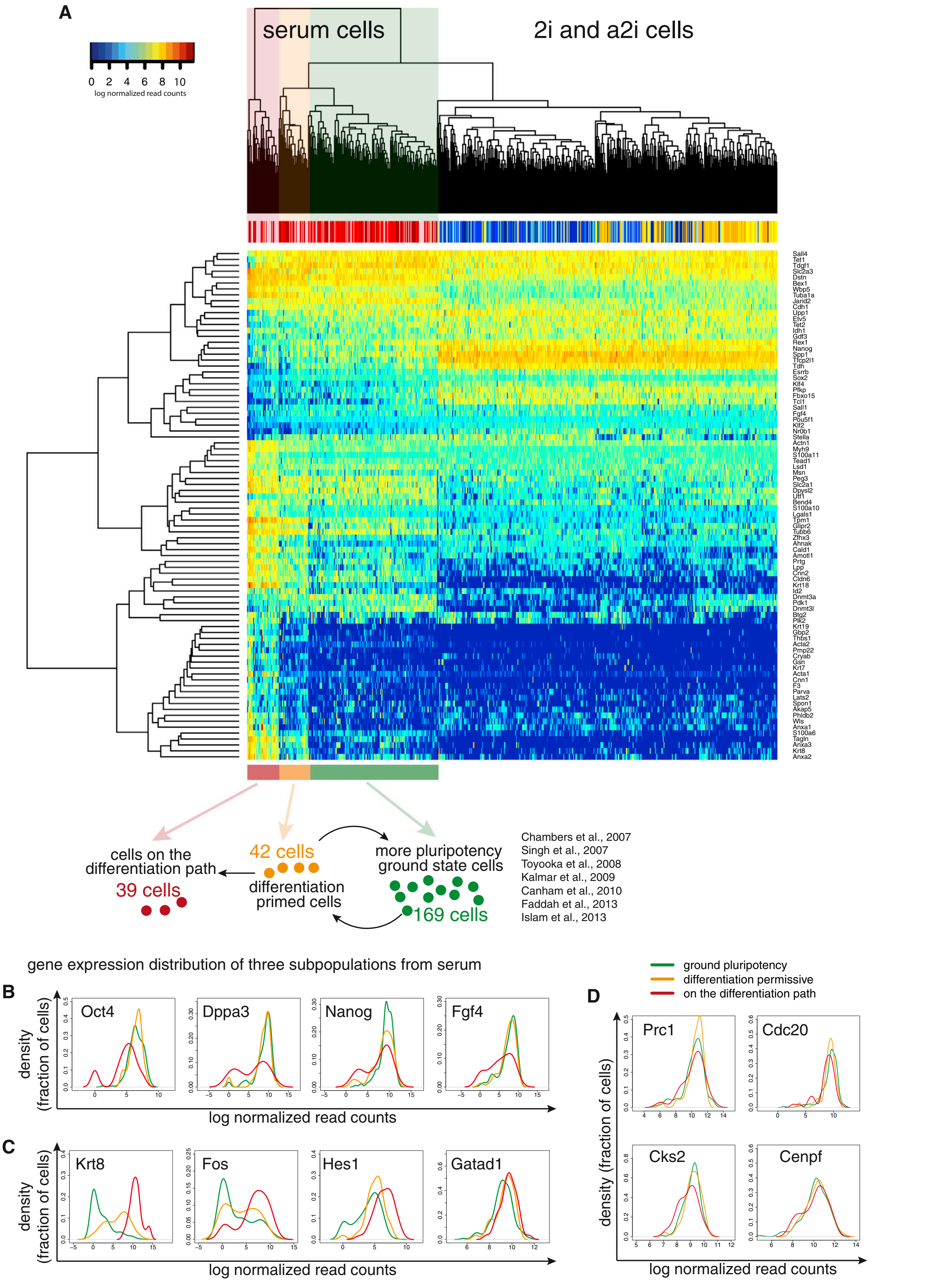

(legend on next page)

blastocyst cells and cells cultured in 2i may originate from differences in the mouse strains and/or sequencing protocols, as well as transcriptome changes resulting from in vitro adaptation. mESCs grown in the inhibitory conditions are the most similar to the in vivo blastocyst cells, while serum cultured cells are somewhat more distant (Figures 4D and 4E), which has also been shown previously using cell ensembles (Boroviak et al., 2014).

The dispersion of mESCs in each culture condition is smaller than the dispersion between cells in the blastocyst. This may be explained by noting that mESCs are derived by clonal expansion and cultured in homogeneous conditions relative to the complexity of cellular niches within the embryo. Moreover, blastocyst cells were obtained from several embryos, thus adding an additional factor that may increase heterogeneity. We quantified global transcriptome noise using the DM measure to compare the heterogeneity of blastocyst cells from three stages (early, mid, and late) versus mESCs cultured in 2i. In all comparisons, blastocyst cells are significantly more heterogeneous than the cultured cells ($p < 10^{-4}$ by Wilcoxon signed rank test).

Identification and Characterization of 2C-like Cells in 2i Medium

To find 2C-like cells in our samples, we examined the expression profile of genes shown previously to have at least 10-fold enrichment in 2C-like marker genes relative to the remaining mESCs (Macfarlan et al., 2012). Hierarchical clustering suggested the presence of ten 2C-like cells in 2i, and none in the a2i or serum culture conditions (2C-like cells may still be present in a2i and serum, but at a very low rate) (Figure 5A).

Globally, the transcriptomes of 2C cells are altered, and only about 50% of reads on average map to exons, compared to 60% in the remaining population in 2i (Figure 5B). Additionally, we observed substantial MERVL expression in 2C-like cells and no expression in the remaining cells (Figure 5C). Subsequently, we calculated the mean expression level of genes identified by Macfarlan et al. (2012) as differentially expressed in 2C-like cells and observed a similar pattern in our data (Figure 5D). Interestingly, we also observe that 2C-like cells have more upregulated genes than downregulated genes (Figure 5E).

It should be noted that globally, 2C-like cells are more similar to 2i cells and blastocyst cells than to cells from the two-cell stage of the in vivo embryo. 2C-like cells cluster together with 2i cells (Figure 5A), and there are only 294 differentially expressed genes between 2C-like cells and the remaining 2i cells (examples in Figure 5F). In comparison, we find 1,700 genes between 2C-like cells and blastocyst and 1,779 between 2C-like cells and two-cell stage cells (for differential expression results see http://www.ebi.ac.uk/teichmann-srv/espresso). In terms of expression of Nanog, Oct4, Sox2, and Myc, 2C-like cells are also similar to 2i cells in comparison to the two-cell and blastocyst stages of the embryo (Figure 5G).

Transcriptional Regulatory Interactions in mESCs Revealed by Gene-to-Gene Correlations

Above, we mined our high-throughput single cell RNA-sequencing data from the perspective of comparing in vitro and in vivo pluripotent cell populations. We next examined its potential as a rich resource for analyzing correlations in gene expression across culture conditions as a strategy to identify candidate regulators of pluripotency. This allows us to develop hypotheses about the transcriptional regulatory networks that regulate pluripotency in mESCs, which is known to be highly interconnected and complex (Boyer et al., 2005; Kim et al., 2008; Loh et al., 2006).

We found that in serum-cultured mESCs, Nanog expression correlates positively with transcription factors (*Esrrb*, *Klf4*, *Oct4/Pou5f1*, *Sox2*, and *Zfp42*), genes involved in DNA methylation (*Dnmt3a*, *Tet1*, and *Tet2*), and other genes such as nuclear receptor *Nr0b1* and histone lysine acetyltransferase *Kat6b*. Nanog is negatively correlated with differentiation regulators including transcription factors *Gata3* and *Klf7* (Figure 6). These findings concur with known interactions in the pluripotency regulatory network, where *Nanog* regulates *Esrrb* (Boyer et al., 2005), *Zfp42* (Shi et al., 2006), and *Klf4* (Zhang et al., 2010). Beyond confirming known interactions, we identified correlations between characterized pluripotency genes and candidate components of the pluripotency transcriptional regulatory network.

Of the candidate genes we selected seven genes for validation: Ptma, Zfp640, Zfp710, Dpy30, Set, Etv5, and Kat6b. First, using ChIP-seq and ChIP-ChIP data from the ESCAPE database, we found that the promoters of six of the candidate genes are bound by core pluripotency genes (Figure 7A) (Xu et al., 2013). To provide insight into the functional role of these genes, we downregulated their expression using a CRISPR/dCas9 repressor that targeted their promoters (Figure 7B) (Gao et al., 2014) before examining changes in their transcriptomes using bulk RNA-sequencing.

We narrowed down our analysis to four cases that showed significant repression of the targeted gene (Figure 7C) and performed differential expression analysis between samples and control gRNA using DESeq. After correcting for multiple hypothesis testing, we found significantly differentially expressed genes in two cases: Ptma and Zfp640 (Figure 7E). In the samples with repressed Ptma, we observed a decrease in the expression of pluripotency genes and an increase in the expression of genes associated with differentiation (Figure 7D, where pluripotency

Figure 3. Population Structure in Serum, 2i, and a2i Cells

(A) Clustering of cells in three culture conditions using a panel of pluripotency factors and differentiation markers. Correlations between cells and genes were calculated using Spearman correlation. Below the heatmap we show a model of the subpopulations of cells grown in serum. The schematic shows cells that express differentiation markers (red), cells that are primed for differentiation while remaining pluripotent (orange), and cells that are closest to the ground state of pluripotency (green).

(B and C) Gene expression distributions of genes that become downregulated (B) and upregulated (C) upon differentiation. Expression is shown as $\log_2$ size factor normalized counts. Oct4 expression is similar in cells closer to the ground state of pluripotency (green) and cells that are primed for differentiation (yellow), and it is much lower in cells we defined as moving toward differentiation (red).

(D) Gene expression distributions of cell-cycle genes.

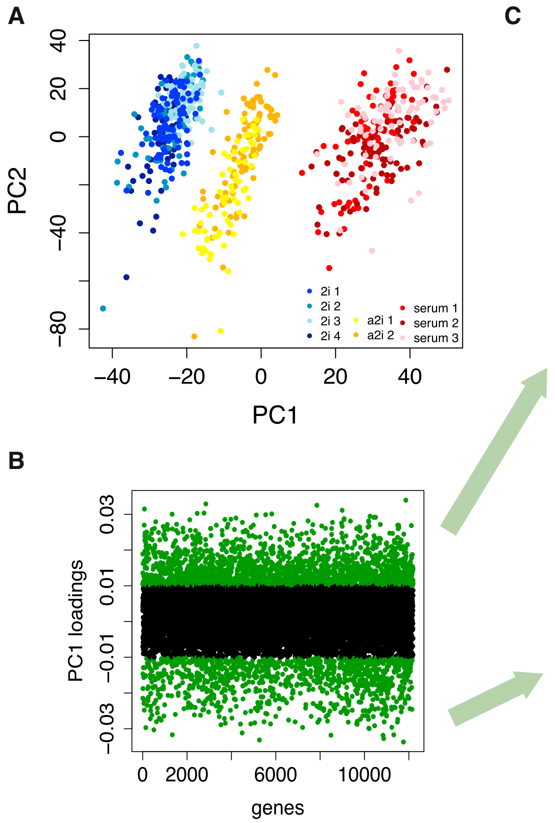

C

GO.ID	Term	p-val
GO:0002053	positive regulation of mesenchymal cell	5.7e-06
GO:0030324	lung development	1.2e-05
GO:0001503	ossification	1.8e-05
GO:0031175	neuron projection development	3.7e-05
GO:0045907	positive regulation of vasoconstriction	5.3e-05
GO:0001666	response to hypoxia	5.8e-05
GO:0000188	inactivation of MAPK activity	5.8e-05
GO:0035335	peptidyl-tyrosine dephosphorylation	6.4e-05
GO:0006096	glycolysis	6.6e-05
GO:0048754	branching morphogenesis of an epithelial tube	7.2e-05
GO:0045597	positive regulation of cell differentiation	9.1e-05
GO:0051094	positive regulation of developmental process	0.00012
GO:0016337	cell-cell adhesion	0.00016
GO:0010604	positive regulation of macromolecule metabolic process	0.00019
GO:0045892	negative regulation of transcription, DNA-templated	0.00021

GO:0000028	ribosomal small subunit assembly	2.5e-06
GO:0000462	maturation of SSU-rRNA from tricistronic rRNA transcript	2.2e-05
GO:0006412	translation	4.8e-05
GO:0050921	positive regulation of chemotaxis	0.00017
GO:0006954	inflammatory response	0.00034
GO:0006749	glutathione metabolic process	0.00039
GO:0008347	glial cell migration	0.00069
GO:0001501	skeletal system development	0.00094
GO:0008284	positive regulation of cell proliferation	0.00107
GO:0032653	regulation of interleukin-10 production	0.00117
GO:0007193	adenylate cyclase-inhibiting G-protein coupled receptor sig. path.	0.00117
GO:0015986	ATP synthesis coupled proton transport	0.00124
GO:0061029	eyelid development in camera-type eye	0.00134
GO:0045087	innate immune response	0.00140
GO:0045071	negative regulation of viral genome replication	0.00178

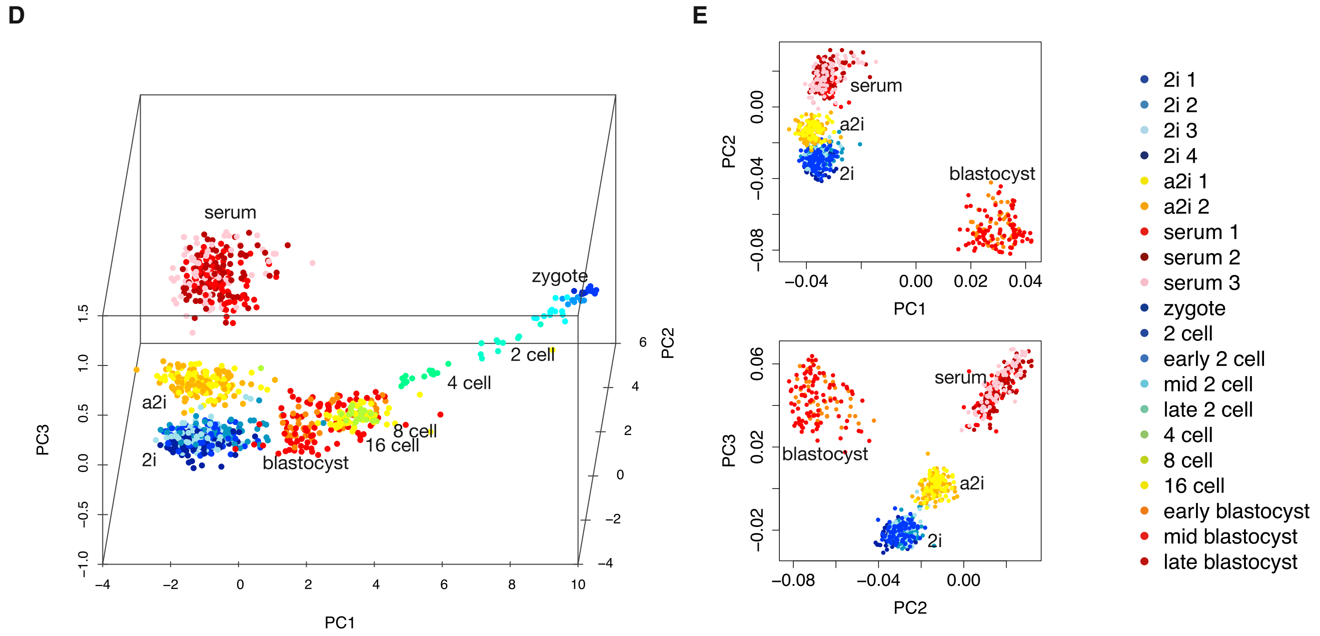

Figure 4. Clustering of mESCs Grown in Serum, 2i, and a2i Media

(A) All cells (n = 704) grown in the three different culture conditions are projected onto the first two principal components. All genes with mean normalized read counts larger than ten were considered and principal component analysis (PCA) was performed.

(B) Distribution of genes contributing to PC1.

(C) GO enrichment analysis of genes most strongly contributing to PC1 separation.

(legend continued on next page)

and differentiation genes are as in Figure 3). Zfp710 and Zfp640 show a similar but milder phenotype, while for Dpy30 there is no clear change in the expression of pluripotency genes (Figure S6C). The lack of effect of Dpy30 downregulation on pluripotency gene expression is consistent with a previous report (Jiang et al., 2011). Overall, these results suggest that Ptma and Zfp640, and potentially also Zfp710, are candidate genes involved in regulating the exit from pluripotency.

DISCUSSION

Here, using single cell RNA-sequencing, we quantified features of cell-to-cell gene expression heterogeneity in mESCs cultured in three different culture conditions. Previous studies had assumed, based on expression of key pluripotency genes, that cells cultured in mESCs are more heterogeneous. Surprisingly, we found that on a global level, cells grown in 2i, a2i, and serum are indistinguishable in terms of transcriptome-wide heterogeneity. Gene expression heterogeneity in specific subsets of genes instead uniquely defines each pluripotent state.

Our results show that mESCs form transcriptomically distinct cell populations depending upon the growth medium (serum, 2i, or a2i), with cells cultured in 2i and a2i being the most similar to each other. When compared to single cells from different stages of mouse embryonic development, all three sets of cultured mESCs are closest to cells from the blastocyst stage, which is the stage from which the cells were extracted originally. The 2i and a2i cultured ESCs seem more similar to the blastocyst cells than serum cells. Additionally, we observed that 2C-like cells are globally more similar to blastocysts than to two-cell stage embryonic cells.

Recently, single cell RNA-sequencing of serum-grown mESCs (Islam et al., 2014) showed a subpopulation with low *Nanog* expression. Additionally, a qPCR study using a panel of 48 pluripotency markers showed that cells cultured in serum exist in two distinct states, with a small number of cells appearing to reside in an intermediate state (Papatsenko et al., 2015). We extended this analysis to identify two smaller subsets of differentiated-committed and intermediate mESCs and a larger self-renewing population. The first shows clear downregulation of *Oct4* and *Sox2* and a slower cell cycle, suggestive of irreversible commitment. In contrast, the intermediate population with higher expression of *Oct4* and *Sox2* may retain the capacity to reacquire pluripotency. Importantly, we also found that the mESC subset that expresses high levels of Nanog in serum is not similar to ‘‘ground state pluripotency’’ 2i cells.

a2i medium has been described as an alternative ground state that can be achieved through the use of a different inhibitor (Shimizu et al., 2012). As expected, a2i is not identical to 2i, but we believe that it is rightfully called an alternative ground state: on the transcriptome level, especially with respect to pluripotency genes, a2i cells are similar to 2i and in vivo blastocyst cells. In 2i and a2i media, there are no subpopulations of differentiating mESCs; hence, pluripotency genes are expressed more homogeneously. Despite these similarities, it is intriguing to note that a2i cells have a cellular RNA content similar to serum-cultured cells, while 2i cells contain about half as much RNA on average, independent of cell-cycle stage. It should be noted that Myc is differentially upregulated in a2i cells compared to 2i cells. As Myc has recently been shown to behave as a transcriptional amplifier of active genes (Lin et al., 2012; Nie et al., 2012), it provides a potential mechanistic basis for the elevated mRNA content in a2i cells.

We observed a relationship between variability in the expression levels of cell-cycle genes and the length of the cell cycle. mESCs cultured in serum have the lowest level of gene expression heterogeneity and mESCs in 2i have the highest, which correlates negatively with doubling times in culture (doubling times were quickest for serum and slowest for 2i). For dividing populations where the cell cycle is very slow, such as HSCs, it is possible to assign cells to one of four cell-cycle stages, but this is more challenging for cells that cycle more quickly (Tsang et al., 2015).

In 2i, but not in a2i, we observed a subpopulation of 2C-like cells that also contribute to heterogeneity within the 2i population. As they are similar to the majority of 2i cells and rare, their contribution to the global heterogeneity of 2i cells is much smaller than the three distinct subpopulations in serum. It is worth noting that our results show that 2C-like cells are not particularly similar to cells at the two-cell stage of the embryo, as was suggested previously.

Finally, our data and methodology allowed us to find new genes involved in the pluripotency network, which we validated using CRISPR repression. We found that downregulating Zfp640, Zfp710, and Ptma affected the expression of both pluripotency and differentiation genes. Ptma repression resulted in the strongest deviation from control samples, and we infer that these cells deviate from pluripotency toward a differentiated state. Interestingly, Ptma is a well-known gene encoding prothymosin alpha, which upon cleavage becomes thymosin alpha, a peptide that has been well studied in the context of immunity and that is used in the treatment of Hepatitis B and C and cancer (Ciancio and Rizzetto, 2010; Garaci et al., 2012; Ioannou et al., 2012). The mode of action of Ptma has been studied in cancer and immune cells, and it has been shown to play a role in proliferation through mechanisms involving chromatin remodeling and interaction with numerous pathways associated with pluripotency maintenance such as the JAK-STAT pathway, the PI3K-Akt pathway, and the NF-κB pathway (George and Brown, 2010; Guo et al., 2015; Yang et al., 2004).

In summary, single-cell transcriptomics has allowed us to gain deep insights into the subpopulation structure within mESC cultures. These results emphasize the power of transcriptomics at single-cell resolution for understanding multiple biological processes.

(D) PCA loading plot of the Spearman’s rank correlation coefficients from mESCs and single cells of mouse preimplantation embryos (Deng et al., 2014) showing the mapping of mESCs in mouse development stages. The cells are visualized by loadings of the first three principal components of the Spearman’s rank correlation matrix between cells, where we used the same expression cutoff as that employed by Deng et al.

(E) PCA of Spearman’s rank correlation matrix between cells from three conditions and blastocyst. The first three components are shown.

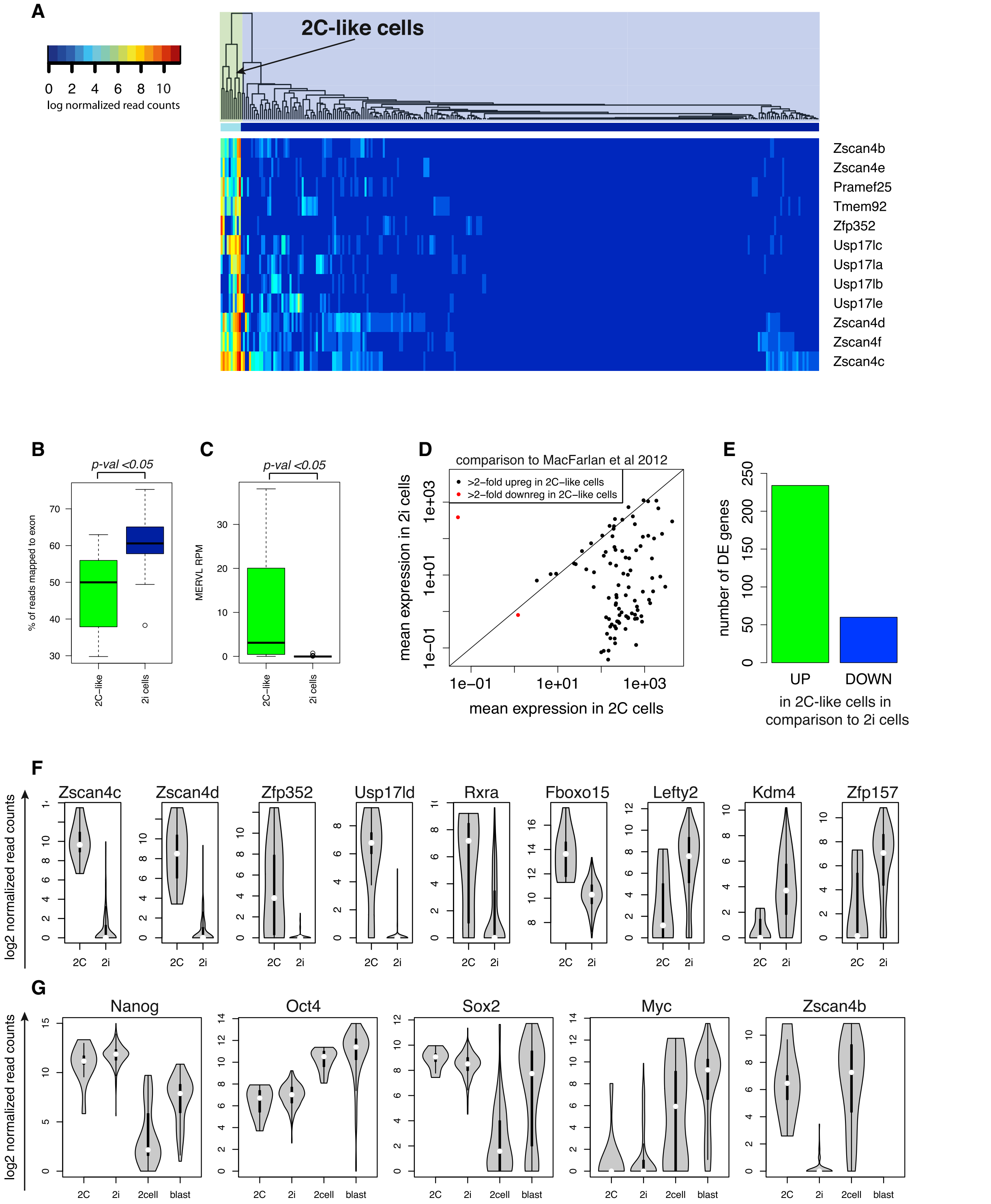

Figure 5. 2C-like Population

(A) Clustering of cells grown in 2i using markers of the 2C-like state (Macfarlan et al., 2012). Correlations were calculated using Spearman correlation. The dendrogram divides cells into two groups, one of which contains ten cells expressing 2C-markers.

(B) Boxplot showing percentage of reads mapping to the exons in both subpopulations of cells in 2i. p was calculated using a Wilcoxon test.

(C) Boxplot showing RPM (reads per million) mapping to the MERVL retrovirus in both subpopulations of cells in 2i. p was calculated using a Wilcoxon test.

(D) Mean expression of genes reported to be at least 2-fold upregulated or downregulated in 2C-like cells (Macfarlan et al., 2012) in cells that we identified as 2C-like cells and in the remaining 2i cells.

(legend continued on next page)

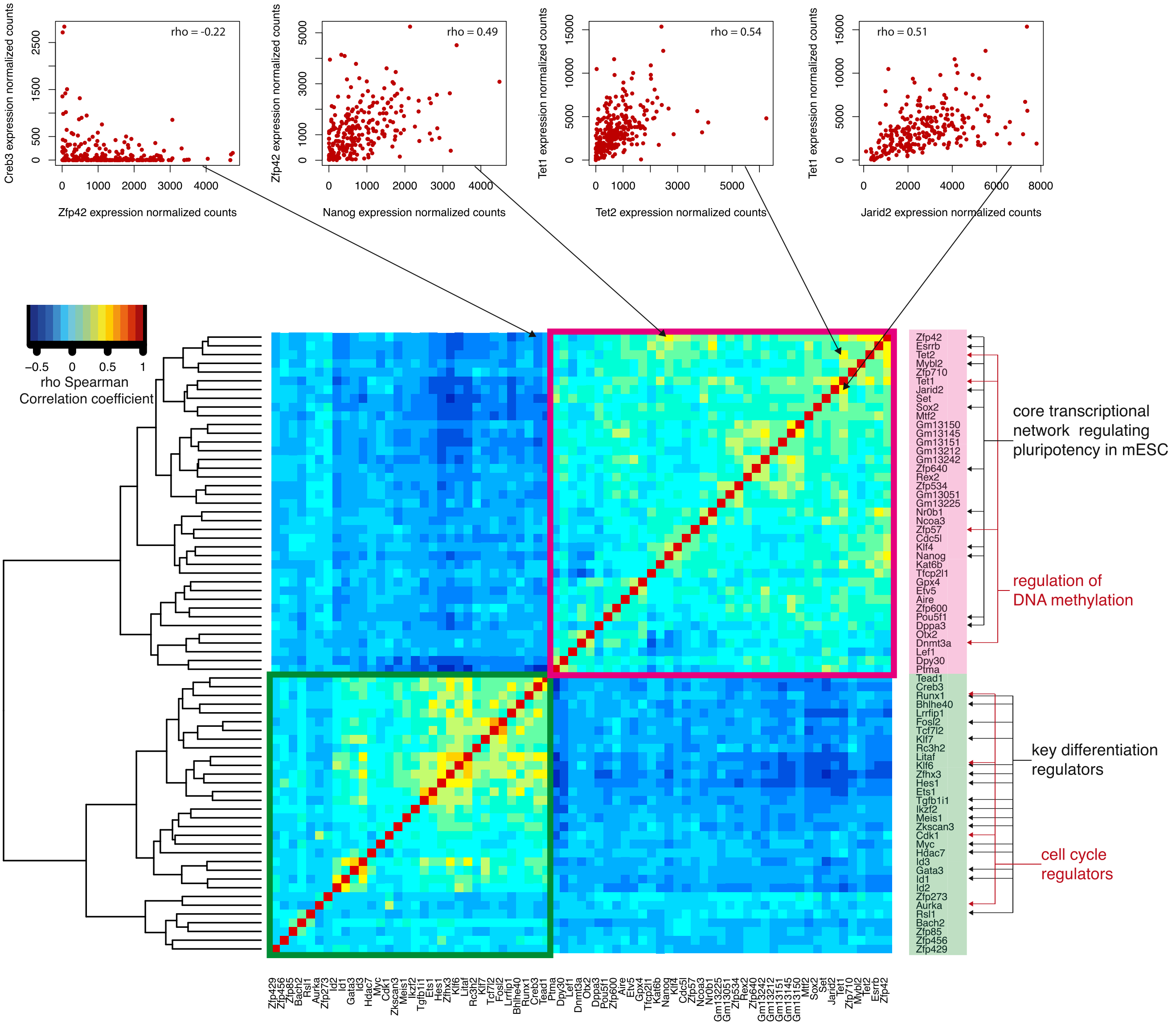

Figure 6. Spearman Correlation Matrix of Transcription Factors and Key Pluripotency Genes

The heatmap shows the correlation coefficients between a set of transcription factors and other key genes involved in pluripotency. Above are examples of genes with expression patterns that correlate positively and negatively (from the left: Zfp42 and Creb3, Zfp42 and Nanog, Tet1 and Tet2, Tet1 and Jarid2).

EXPERIMENTAL PROCEDURES

Cell Culture of mESCs

The G4 (C57BL/6Ncr x 129S6/SvEvTac) mouse hybrid (George et al., 2007) ESCs were obtained from Mount Sinai Hospital and were maintained on STO feeders in serum-containing media at 5% CO_2 and 37°C. They were sub-cloned, and a line with normal karyotype was selected for further analysis. The cells were split onto gelatinized plates (10 cm, Corning) and expanded in serum-containing media or chemically defined media (standard 2i or alternative 2i) for at least three passages. Cells were harvested by trypsinization (0.05% trypsin/EDTA, GIBCO) for 10 min, at which point they reached 70%–80% confluence for single-cell capture.

The three media are as follows:

(1) Serum-containing media: Knockout DMEM (GIBCO), 1X penicillin-streptomycin-glutamine (GIBCO), 1X non-essential amino acids (GIBCO), 100 U/ml recombinant human leukemia inhibitory factor (Millipore), 15% fetal bovine serum (HyClone), 0.1mM β-mercaptoethanol (Sigma).

(E) Barplot showing the number of significantly (DESeq, adjusted $p < 0.05$) upregulated and downregulated genes in 2C-like cells.

(F) Gene expression distributions of genes that become upregulated or downregulated in 2C-like cells (2C) in comparison to remaining cells grown in 2i media (2i).

(G) Expression of key pluripotency genes in 2C-like cells (2C), the rest of cells grown in 2i media (2i), cells from the two-cell stage (2cell), and cells from the blastocyst stage (blast) of the embryo.

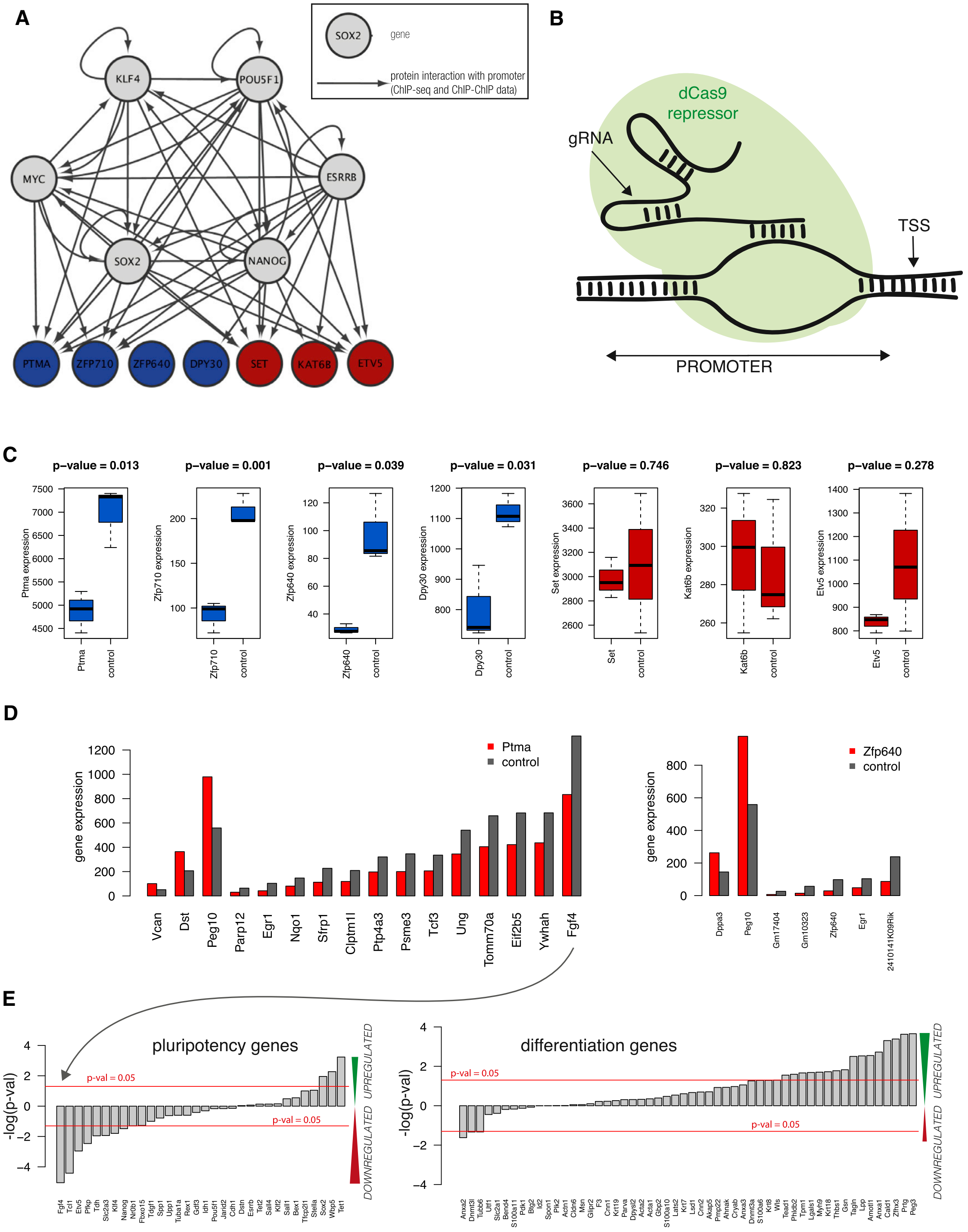

Figure 7. Validation of Putative Members of the Pluripotency Network

(A) Network showing known interactions of core pluripotency factors with the novel candidate genes. Data obtained from ChIP-seq and ChIP-ChIP experiments from ESCAPE database.

(B) Schematic showing experimental design. Catalytically inactive Cas9 and gRNA bind to the promoter of the targeted gene, occluding it and competing for binding with transcription factors and polymerases.

(C) Expression level of repressed genes in samples and control. Targets with significant repression are in blue.

(D) Barplot of gene expression levels of significantly differentially expressed genes in Ptma- and Zfp640-repressed samples (DESeq, multiple hypotheses testing adjusted $p < 0.05$).

(E) Barplots showing the logarithm of p values for differential expression from DESeq of pluripotency (left) and differentiation (right) genes in the Ptma knockdown samples. For genes that are downregulated, the numbers are negative, and they are positive for upregulated genes. The red line indicates a p threshold of 0.05.

(2) Standard 2i media: N2B27 basal media (NDiff 227, StemCells), 100 U/ml recombinant human LIF (Millipore), 1 μM PD0325901 (Stemgent), 3 μM CHIR99021 (Stemgent).
(3) Alternative 2i media: N2B27 basal media (NDiff 227, StemCells), 100 U/ml recombinant human LIF (Millipore), 1 μM CGP77675 (Sigma), 3 μM CHIR99021 (Stemgent).

cDNA Library Preparation from Single Cells using the Fluidigm C1

For each culture condition, 4,000 cells were loaded on to a 10–17 μm Fluidigm C1 Single-Cell Auto Prep IFC, and cell capture was performed according to the manufacturer's instructions. The capture efficiency was inspected using a microscope to remove samples from the analysis with more than one cell captured. Upon capture, reverse transcription and cDNA preamplification were performed in the 10–17 μm Fluidigm C1 Single-Cell Auto Prep IFC using the SMARTer PCR cDNA Synthesis Kit (Clontech) and the Advantage 2 PCR Kit (Ramsköld et al., 2012). cDNA was harvested and diluted to a range of 0.1–0.3 ng/μl and Nextera libraries were prepared using the Nextera XT DNA Sample Preparation Kit and the Nextera Index Kit (Illumina) following the instructions in the Fluidigm manual "Using the C1™ Single-Cell Auto Prep System to Generate mRNA from Single Cells and Libraries for Sequencing." Libraries from one chip were pooled, and paired-end 100 bp sequencing was performed on four lanes of an Illumina HiSeq2000.

Bulk RNA-Sequencing

Bulk RNA-sequencing libraries were prepared and sequenced using the Wellcome Trust Sanger Institute sample preparation pipeline with Illumina's TruSeq RNA Sample Preparation v2 Kit. RNA was extracted from 1–2 million cells using the QIAGEN RNA Purification Kit on a QiaCube robot. The quality of the RNA sample was checked using gel electrophoresis. For library preparation, poly-A RNA was purified from total RNA using oligo-dT magnetic pull-down. Subsequently, mRNA was fragmented using metal-ion catalyzed hydrolysis. The cDNA was synthesized using random hexamer priming, and end repair was performed to obtain blunt ends. A-tailing was done to enable subsequent ligation of Illumina paired-end sequencing adapters, and samples were multiplexed at this stage. The resulting library was amplified using 10 cycles of PCR, substituting the Kapa Hifi polymerase for the polymerase in the Illumina TruSeq Kit. Samples were diluted to 4nM, and 100 bp paired-end sequencing was carried out on an Illumina HiSeq2000. Sequencing Quality Control was performed by the Sanger sequencing facility.

Mapping Reads

Paired-end reads were mapped simultaneously to the *Mus musculus* genome (Ensembl version 38.73) using GSNAP (version gmap-2014-05-15_v2) using default parameters. Subsequently we counted reads for each gene with htseq-count and normalized them with size factors calculated from DESeq as reported previously (Brennecke et al., 2013). We also applied location and scale adjustments to the normalized read counts to remove technical variation among multiple batches (Supplemental Experimental Procedures).

Quality Control of Cells

To exclude poor quality cells from the downstream analysis, we removed cells according to the following criteria: (1) empty capture sites or capture sites with multiple cells or debris, as defined by visual inspection of the chip; (2) cells that had fewer than 500,000 reads mapped to exons; and (3) cells that had over 10% reads mapped to mitochondrial genes (refer to Supplemental Experimental Procedures for details).

Candidate Gene Expression Repression with CRISPR

45 guide RNAs targeting promoter regions of 7 candidate genes (Ptma, Set, Zfp640, Zfp710, Kat6b, Dpy30, and Etv5) were cloned into gRNA-mCherry plasmid (for a list of sequences, refer to Table S3). GFP-Oct4 reporter strain ESCs (Silva et al., 2008) were transfected with (1) Cas9 repressor-BFP, (2) transposase, and (3) a cocktail of gRNA plasmids (Gao et al., 2014) targeting the gene of interest in a 1:1 ratio using Lipofectamine2000 (Life Technologies). Subsequently, cells were cultured in medium containing 15% serum and LIF for 4 days before 10,000 mCherry and BFP-positive cells were sorted for each sample. RNA was extracted using QIAGEN RNeasy Mini Kit. The SmartSeq2 protocol was used for reverse transcription and amplification of cDNA (Picelli et al., 2014). Sequencing libraries were prepared using Nextera XT Kit according to the manufacturer's guidelines, barcoded with Nextera XT Dual Index Kit, and sequenced on an Illumina HiSeq2500 in rapid mode.

ACCESSION NUMBERS

Sequencing data are available in the ArrayExpress database (http://www.ebi.ac.uk/arrayexpress) under accession number E-MTAB-2600.

SUPPLEMENTAL INFORMATION

Supplemental Information for this article includes Supplemental Experimental Procedures, seven figures, and three tables and can be found with this article online at http://dx.doi.org/10.1016/j.stem.2015.09.011.

AUTHOR CONTRIBUTIONS

A.A.K. carried out single-cell and bulk RNA-sequencing experiments, analyzed and interpreted data, and prepared figures and the manuscript; J.K.K. developed and carried out statistical and bioinformatics analyses and figure preparation and contributed to manuscript preparation; J.T. cultured cells, performed the doubling time experiment, designed the CRISPR repression experiment, interpreted data, and contributed to the manuscript; T.I. carried out bioinformatics and statistical analyses, including that involved in figure preparation; J.H. developed the website; K.N.N. performed NPC single-cell differentiation time course experiments; A.C.T. performed NPC single-cell differentiation time course experiments; X.G. performed CRISPR repression experiments; M.B. supported NPC single-cell differentiation time course experiments; P.L. advised on cell culture conditions and ESC biology; J.C.M. developed and advised on statistics and bioinformatics methods and analysis and contributed to manuscript preparation; and S.A.T. designed experiments, advised on analysis, and contributed to manuscript preparation.

ACKNOWLEDGMENTS

We are grateful to David Adams and Anne Ferguson-Smith for mESCs, to Nathalie Smerdon for sequencing support, and to Xiuwei Zhang for helpful discussions. We thank Rebecca Berrens, Julia Tischler, Wolf Reik, and Azim Surani for their comments on the manuscript. We acknowledge the BBSRC for a CASE Studentship to A.A.K. and the Sanger-EBI Single Cell Genomics Centre for support. This work was supported by a Wellcome Trust Fellowship to A.T. (103977).

Received: June 17, 2014
Revised: March 7, 2015
Accepted: September 15, 2015
Published: October 1, 2015

REFERENCES

Anders, S., and Huber, W. (2010). Differential expression analysis for sequence count data. Genome Biol. *11*, R106.

Avilion, A.A., Nicolis, S.K., Pevny, L.H., Perez, L., Vivian, N., and Lovell-Badge, R. (2003). Multipotent cell lineages in early mouse development depend on SOX2 function. Genes Dev. *17*, 126–140.

Bibel, M., Richter, J., Lacroix, E., and Barde, Y.A. (2007). Generation of a defined and uniform population of CNS progenitors and neurons from mouse embryonic stem cells. Nat. Protoc. *2*, 1034–1043.

Boroviak, T., Loos, R., Bertone, P., Smith, A., and Nichols, J. (2014). The ability of inner-cell-mass cells to self-renew as embryonic stem cells is acquired following epiblast specification. Nat. Cell Biol. *16*, 516–528.

Boyer, L.A., Lee, T.I., Cole, M.F., Johnstone, S.E., Levine, S.S., Zucker, J.P., Guenther, M.G., Kumar, R.M., Murray, H.L., Jenner, R.G., et al. (2005). Core transcriptional regulatory circuitry in human embryonic stem cells. Cell *122*, 947–956.

Bradley, A., Evans, M., Kaufman, M.H., and Robertson, E. (1984). Formation of germ-line chimaeras from embryo-derived teratocarcinoma cell lines. Nature *309*, 255–256.

Brennecke, P., Anders, S., Kim, J.K., Kołodziejczyk, A.A., Zhang, X., Proserpio, V., Baying, B., Benes, V., Teichmann, S.A., Marioni, J.C., and Heisler, M.G. (2013). Accounting for technical noise in single-cell RNA-seq experiments. Nat. Methods *10*, 1093–1095.

Burdon, T., Stracey, C., Chambers, I., Nichols, J., and Smith, A. (1999). Suppression of SHP-2 and ERK signalling promotes self-renewal of mouse embryonic stem cells. Dev. Biol. *210*, 30–43.

Canham, M.A., Sharov, A.A., Ko, M.S., and Brickman, J.M. (2010). Functional heterogeneity of embryonic stem cells revealed through translational amplification of an early endodermal transcript. PLoS Biol. *8*, e1000379.

Chambers, I., Colby, D., Robertson, M., Nichols, J., Lee, S., Tweedie, S., and Smith, A. (2003). Functional expression cloning of Nanog, a pluripotency sustaining factor in embryonic stem cells. Cell *113*, 643–655.

Chambers, I., Silva, J., Colby, D., Nichols, J., Nijmeijer, B., Robertson, M., Vrana, J., Jones, K., Grotewold, L., and Smith, A. (2007). Nanog safeguards pluripotency and mediates germline development. Nature *450*, 1230–1234.

Chang, H.H., Hemberg, M., Barahona, M., Ingber, D.E., and Huang, S. (2008). Transcriptome-wide noise controls lineage choice in mammalian progenitor cells. Nature *453*, 544–547.

Chen, X., Xu, H., Yuan, P., Fang, F., Huss, M., Vega, V.B., Wong, E., Orlov, Y.L., Zhang, W., Jiang, J., et al. (2008). Integration of external signaling pathways with the core transcriptional network in embryonic stem cells. Cell *133*, 1106–1117.

Ciancio, A., and Rizzetto, M. (2010). Thymalfasin in the treatment of hepatitis B and C. Ann. N Y Acad. Sci. *1194*, 141–146.

Deng, Q., Ramsköld, D., Reinius, B., and Sandberg, R. (2014). Single-cell RNA-seq reveals dynamic, random monoallelic gene expression in mammalian cells. Science *343*, 193–196.

Ding, B., Zheng, L., Zhu, Y., Li, N., Jia, H., Ai, R., Wildberg, A., and Wang, W. (2015). Normalization and noise reduction for single cell RNA-seq experiments. Bioinformatics *31*, 2225–2227.

Etzrodt, M., Endele, M., and Schroeder, T. (2014). Quantitative single-cell approaches to stem cell research. Cell Stem Cell *15*, 546–558.

Evans, M.J., and Kaufman, M.H. (1981). Establishment in culture of pluripotential cells from mouse embryos. Nature *292*, 154–156.

Faddah, D.A., Wang, H., Cheng, A.W., Katz, Y., Buganim, Y., and Jaenisch, R. (2013). Single-cell analysis reveals that expression of nanog is biallelic and equally variable as that of other pluripotency factors in mouse ESCs. Cell Stem Cell *13*, 23–29.

Festuccia, N., Osorno, R., Halbritter, F., Karwacki-Neisius, V., Navarro, P., Colby, D., Wong, F., Yates, A., Tomlinson, S.R., and Chambers, I. (2012). Esrrb is a direct Nanog target gene that can substitute for Nanog function in pluripotent cells. Cell Stem Cell *11*, 477–490.

Ficz, G., Hore, T.A., Santos, F., Lee, H.J., Dean, W., Arand, J., Krueger, F., Oxley, D., Paul, Y.L., Walter, J., et al. (2013). FGF signaling inhibition in ESCs drives rapid genome-wide demethylation to the epigenetic ground state of pluripotency. Cell Stem Cell *13*, 351–359.

Gao, X., Tsang, J.C., Gaba, F., Wu, D., Lu, L., and Liu, P. (2014). Comparison of TALE designer transcription factors and the CRISPR/dCas9 in regulation of gene expression by targeting enhancers. Nucleic Acids Res. *42*, e155.

Garaci, E., Pica, F., Serafino, A., Balestrieri, E., Matteucci, C., Moroni, G., Sorrentino, R., Zonfrillo, M., Pierimarchi, P., and Sinibaldi-Vallebona, P. (2012). Thymosin α1 and cancer: action on immune effector and tumor target cells. Ann. N Y Acad. Sci. *1269*, 26–33.

George, E.M., and Brown, D.T. (2010). Prothymosin alpha is a component of a linker histone chaperone. FEBS Lett. *584*, 2833–2836.

George, S.H., Gertsenstein, M., Vintersten, K., Korets-Smith, E., Murphy, J., Stevens, M.E., Haigh, J.J., and Nagy, A. (2007). Developmental and adult phenotyping directly from mutant embryonic stem cells. Proc. Natl. Acad. Sci. USA *104*, 4455–4460.

Grün, D., Kester, L., and van Oudenaarden, A. (2014). Validation of noise models for single-cell transcriptomics. Nat. Methods *11*, 637–640.

Guo, Y., Chang, H., Li, J., Xu, X.Y., Shen, L., Yu, Z.B., and Liu, W.C. (2015). Thymosin alpha 1 suppresses proliferation and induces apoptosis in breast cancer cells through PTEN-mediated inhibition of PI3K/Akt/mTOR signaling pathway. Apoptosis *20*, 1109–1121.

Hayashi, K., Lopes, S.M., Tang, F., and Surani, M.A. (2008). Dynamic equilibrium and heterogeneity of mouse pluripotent stem cells with distinct functional and epigenetic states. Cell Stem Cell *3*, 391–401.

Ioannou, K., Samara, P., Livaniou, E., Derhovanessian, E., and Tsitsilonis, O.E. (2012). Prothymosin alpha: a ubiquitous polypeptide with potential use in cancer diagnosis and therapy. Cancer Immunol. Immunother. *61*, 599–614.

Islam, S., Zeisel, A., Joost, S., La Manno, G., Zajac, P., Kasper, M., Lönnerberg, P., and Linnarsson, S. (2014). Quantitative single-cell RNA-seq with unique molecular identifiers. Nat. Methods *11*, 163–166.

Jiang, H., Shukla, A., Wang, X., Chen, W.Y., Bernstein, B.E., and Roeder, R.G. (2011). Role for Dpy-30 in ES cell-fate specification by regulation of H3K4 methylation within bivalent domains. Cell *144*, 513–525.

Kalmar, T., Lim, C., Hayward, P., Muñoz-Descalzo, S., Nichols, J., Garcia-Ojalvo, J., and Martinez Arias, A. (2009). Regulated fluctuations in nanog expression mediate cell fate decisions in embryonic stem cells. PLoS Biol. *7*, e1000149.

Kim, J., Chu, J., Shen, X., Wang, J., and Orkin, S.H. (2008). An extended transcriptional network for pluripotency of embryonic stem cells. Cell *132*, 1049–1061.

Kumar, R.M., Cahan, P., Shalek, A.K., Satija, R., DaleyKeyser, A.J., Li, H., Zhang, J., Pardee, K., Gennert, D., Trombetta, J.J., et al. (2014). Deconstructing transcriptional heterogeneity in pluripotent stem cells. Nature *516*, 56–61.

Kunath, T., Saba-El-Leil, M.K., Almousailleakh, M., Wray, J., Meloche, S., and Smith, A. (2007). FGF stimulation of the Erk1/2 signalling cascade triggers transition of pluripotent embryonic stem cells from self-renewal to lineage commitment. Development *134*, 2895–2902.

Li, X., Zhu, L., Yang, A., Lin, J., Tang, F., Jin, S., Wei, Z., Li, J., and Jin, Y. (2011). Calcineurin-NFAT signaling critically regulates early lineage specification in mouse embryonic stem cells and embryos. Cell Stem Cell *8*, 46–58.

Lin, C.Y., Lovén, J., Rahl, P.B., Paranal, R.M., Burge, C.B., Bradner, J.E., Lee, T.I., and Young, R.A. (2012). Transcriptional amplification in tumor cells with elevated c-Myc. Cell *151*, 56–67.

Loh, Y.H., Wu, Q., Chew, J.L., Vega, V.B., Zhang, W., Chen, X., Bourque, G., George, J., Leong, B., Liu, J., et al. (2006). The Oct4 and Nanog transcription network regulates pluripotency in mouse embryonic stem cells. Nat. Genet. *38*, 431–440.

MacArthur, B.D., Sevilla, A., Lenz, M., Müller, F.J., Schuldt, B.M., Schuppert, A.A., Ridden, S.J., Stumpf, P.S., Fidalgo, M., Ma'ayan, A., et al. (2012). Nanog-dependent feedback loops regulate murine embryonic stem cell heterogeneity. Nat. Cell Biol. *14*, 1139–1147.

Macfarlan, T.S., Gifford, W.D., Driscoll, S., Lettieri, K., Rowe, H.M., Bonanomi, D., Firth, A., Singer, O., Trono, D., and Pfaff, S.L. (2012). Embryonic stem cell potency fluctuates with endogenous retrovirus activity. Nature *487*, 57–63.

Marks, H., Kalkan, T., Menafra, R., Denissov, S., Jones, K., Hofemeister, H., Nichols, J., Kranz, A., Stewart, A.F., Smith, A., and Stunnenberg, H.G. (2012). The transcriptional and epigenomic foundations of ground state pluripotency. Cell *149*, 590–604.

Marson, A., Levine, S.S., Cole, M.F., Frampton, G.M., Brambrink, T., Johnstone, S., Guenther, M.G., Johnston, W.K., Wernig, M., Newman, J., et al. (2008). Connecting microRNA genes to the core transcriptional regulatory circuitry of embryonic stem cells. Cell *134*, 521–533.

Martello, G., Sugimoto, T., Diamanti, E., Joshi, A., Hannah, R., Ohtsuka, S., Göttgens, B., Niwa, H., and Smith, A. (2012). Esrrb is a pivotal target of the Gsk3/Tcf3 axis regulating embryonic stem cell self-renewal. Cell Stem Cell *11*, 491–504.

Martin, G.R. (1981). Isolation of a pluripotent cell line from early mouse embryos cultured in medium conditioned by teratocarcinoma stem cells. Proc. Natl. Acad. Sci. USA *78*, 7634–7638.

Mitsui, K., Tokuzawa, Y., Itoh, H., Segawa, K., Murakami, M., Takahashi, K., Maruyama, M., Maeda, M., and Yamanaka, S. (2003). The homeoprotein Nanog is required for maintenance of pluripotency in mouse epiblast and ES cells. Cell *113*, 631–642.

Newman, J.R., Ghaemmaghami, S., Ihmels, J., Breslow, D.K., Noble, M., DeRisi, J.L., and Weissman, J.S. (2006). Single-cell proteomic analysis of S. cerevisiae reveals the architecture of biological noise. Nature *441*, 840–846.

Nichols, J., and Smith, A. (2009). Naive and primed pluripotent states. Cell Stem Cell *4*, 487–492.

Nichols, J., Zevnik, B., Anastassiadis, K., Niwa, H., Klewe-Nebenius, D., Chambers, I., Schöler, H., and Smith, A. (1998). Formation of pluripotent stem cells in the mammalian embryo depends on the POU transcription factor Oct4. Cell *95*, 379–391.

Nie, Z., Hu, G., Wei, G., Cui, K., Yamane, A., Resch, W., Wang, R., Green, D.R., Tessarollo, L., Casellas, R., et al. (2012). c-Myc is a universal amplifier of expressed genes in lymphocytes and embryonic stem cells. Cell *151*, 68–79.

Niwa, H., Ogawa, K., Shimosato, D., and Adachi, K. (2009). A parallel circuit of LIF signalling pathways maintains pluripotency of mouse ES cells. Nature *460*, 118–122.

Papatsenko, D., Darr, H., Kulakovskiy, I.V., Waghray, A., Makeev, V.J., MacArthur, B.D., and Lemischka, I.R. (2015). Single-Cell Analyses of ESCs Reveal Alternative Pluripotent Cell States and Molecular Mechanisms that Control Self-Renewal. Stem Cell Reports *5*, 207–220.

Picelli, S., Faridani, O.R., Björklund, A.K., Winberg, G., Sagasser, S., and Sandberg, R. (2014). Full-length RNA-seq from single cells using Smart-seq2. Nat. Protoc. *9*, 171–181.

Ramsköld, D., Luo, S., Wang, Y.C., Li, R., Deng, Q., Faridani, O.R., Daniels, G.A., Khrebtukova, I., Loring, J.F., Laurent, L.C., et al. (2012). Full-length mRNA-Seq from single-cell levels of RNA and individual circulating tumor cells. Nat. Biotechnol. *30*, 777–782.

Reynolds, N., Latos, P., Hynes-Allen, A., Loos, R., Leaford, D., O'Shaughnessy, A., Mosaku, O., Signolet, J., Brennecke, P., Kalkan, T., et al. (2012). NuRD suppresses pluripotency gene expression to promote transcriptional heterogeneity and lineage commitment. Cell Stem Cell *10*, 583–594.

Shi, W., Wang, H., Pan, G., Geng, Y., Guo, Y., and Pei, D. (2006). Regulation of the pluripotency marker Rex-1 by Nanog and Sox2. J. Biol. Chem. *281*, 23319–23325.

Shimizu, T., Ueda, J., Ho, J.C., Iwasaki, K., Poellinger, L., Harada, I., and Sawada, Y. (2012). Dual inhibition of Src and GSK3 maintains mouse embryonic stem cells, whose differentiation is mechanically regulated by Src signaling. Stem Cells *30*, 1394–1404.

Silva, J., Barrandon, O., Nichols, J., Kawaguchi, J., Theunissen, T.W., and Smith, A. (2008). Promotion of reprogramming to ground state pluripotency by signal inhibition. PLoS Biol. *6*, e253.

Singh, A.M., Hamazaki, T., Hankowski, K.E., and Terada, N. (2007). A heterogeneous expression pattern for Nanog in embryonic stem cells. Stem Cells *25*, 2534–2542.

Smith, A.G., Heath, J.K., Donaldson, D.D., Wong, G.G., Moreau, J., Stahl, M., and Rogers, D. (1988). Inhibition of pluripotential embryonic stem cell differentiation by purified polypeptides. Nature *336*, 688–690.

Stegle, O., Teichmann, S.A., and Marioni, J.C. (2015). Computational and analytical challenges in single-cell transcriptomics. Nat. Rev. Genet. *16*, 133–145.

Toyooka, Y., Shimosato, D., Murakami, K., Takahashi, K., and Niwa, H. (2008). Identification and characterization of subpopulations in undifferentiated ES cell culture. Development *135*, 909–918.

Tsang, J.C.H., Yu, Y., Burke, S., Buettner, F., Wang, C., Kolodziejczyk, A.A., Teichmann, S.A., Lu, L., and Liu, P. (2015). Single-cell transcriptomic reconstruction reveals cell cycle and multi-lineage differentiation defects in Bcl11a-deficient hematopoietic stem cells. Genome Biol. *16*, http://dx.doi.org/10.1186/s13059-015-0739-5.

van den Berg, D.L., Zhang, W., Yates, A., Engelen, E., Takacs, K., Bezstarosti, K., Demmers, J., Chambers, I., and Poot, R.A. (2008). Estrogen-related receptor beta interacts with Oct4 to positively regulate Nanog gene expression. Mol. Cell. Biol. *28*, 5986–5995.

Williams, R.L., Hilton, D.J., Pease, S., Willson, T.A., Stewart, C.L., Gearing, D.P., Wagner, E.F., Metcalf, D., Nicola, N.A., and Gough, N.M. (1988). Myeloid leukaemia inhibitory factor maintains the developmental potential of embryonic stem cells. Nature *336*, 684–687.

Wray, J., Kalkan, T., Gomez-Lopez, S., Eckardt, D., Cook, A., Kemler, R., and Smith, A. (2011). Inhibition of glycogen synthase kinase-3 alleviates Tcf3 repression of the pluripotency network and increases embryonic stem cell resistance to differentiation. Nat. Cell Biol. *13*, 838–845.

Xu, H., Baroukh, C., Dannenfelser, R., Chen, E.Y., Tan, C.M., Kou, Y., Kim, Y.E., Lemischka, I.R., and Ma'ayan, A. (2013). ESCAPE: database for integrating high-content published data collected from human and mouse embryonic stem cells. Database (Oxford) *2013*, bat045.

Yang, C.H., Murti, A., Baker, S.J., Frangou-Lazaridis, M., Vartapetian, A.B., Murti, K.G., and Pfeffer, L.M. (2004). Interferon induces the interaction of prothymosin-alpha with STAT3 and results in the nuclear translocation of the complex. Exp. Cell Res. *298*, 197–206.

Ye, S., Li, P., Tong, C., and Ying, Q.L. (2013). Embryonic stem cell self-renewal pathways converge on the transcription factor Tfcp2l1. EMBO J. *32*, 2548–2560.

Yeo, J.C., Jiang, J., Tan, Z.Y., Yim, G.R., Ng, J.H., Göke, J., Kraus, P., Liang, H., Gonzales, K.A., Chong, H.C., et al. (2014). Klf2 is an essential factor that sustains ground state pluripotency. Cell Stem Cell *14*, 864–872.

Ying, Q.L., Nichols, J., Chambers, I., and Smith, A. (2003). BMP induction of Id proteins suppresses differentiation and sustains embryonic stem cell self-renewal in collaboration with STAT3. Cell *115*, 281–292.

Ying, Q.L., Wray, J., Nichols, J., Batlle-Morera, L., Doble, B., Woodgett, J., Cohen, P., and Smith, A. (2008). The ground state of embryonic stem cell self-renewal. Nature *453*, 519–523.

Zhang, P., Andrianakos, R., Yang, Y., Liu, C., and Lu, W. (2010). Kruppel-like factor 4 (Klf4) prevents embryonic stem (ES) cell differentiation by regulating Nanog gene expression. J. Biol. Chem. *285*, 9180–9189.

Cell Stem Cell

Supplemental Information

Single Cell RNA-Sequencing of Pluripotent States Unlocks Modular Transcriptional Variation

Aleksandra A. Kolodziejczyk, Jong Kyoung Kim, Jason C.H. Tsang, Tomislav Ilicic, Johan Henriksson, Kedar N. Natarajan, Alex C. Tuck, Xuefei Gao, Marc Bühler, Pentao Liu, John C. Marioni, and Sarah A. Teichmann

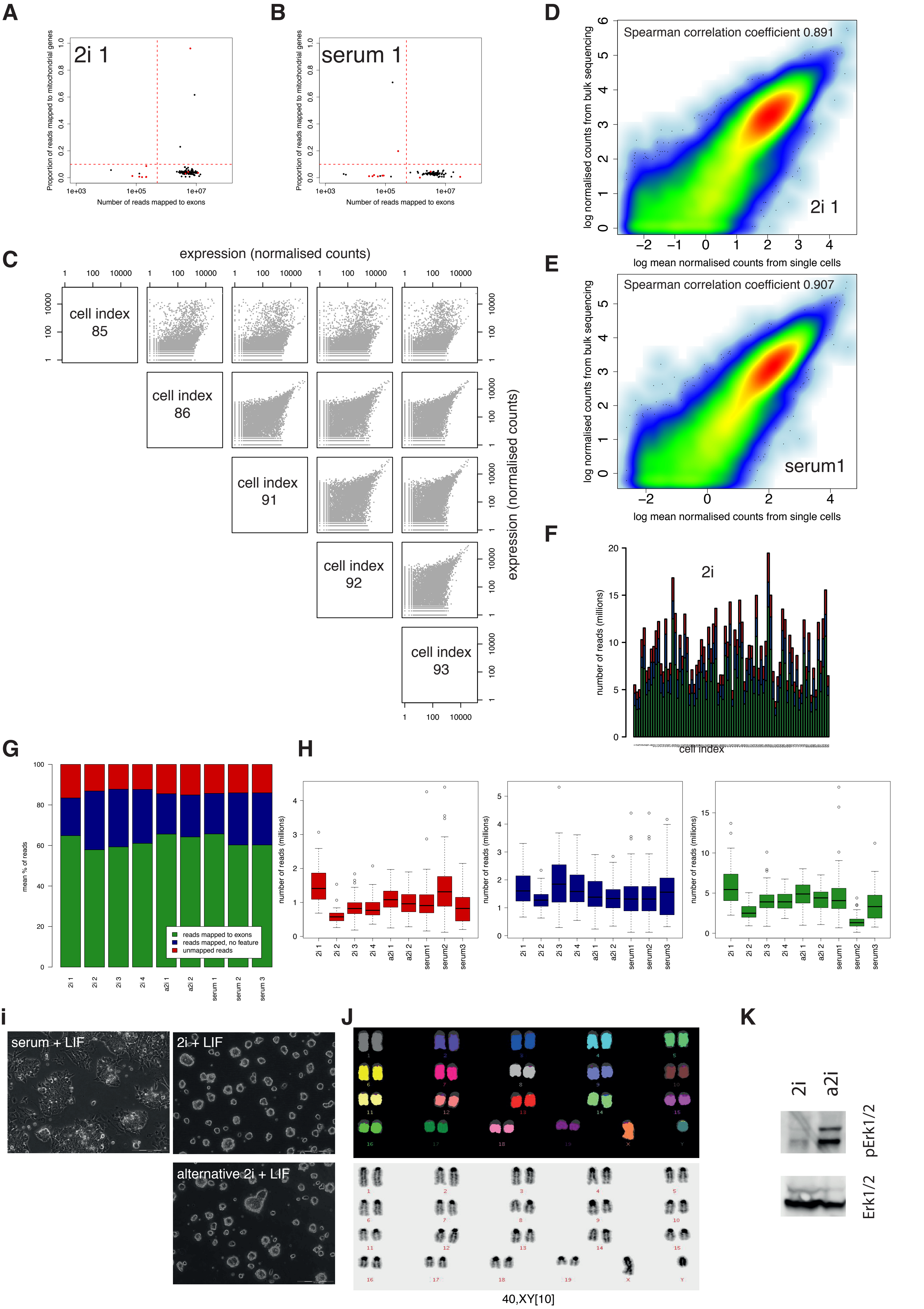
A
B
C
D
E
F
G
H
i
J
K
2i 1
serum 1
Proportion of reads mapped to mitochondrial genes
Number of reads mapped to exons
expression (normalised counts)
cell index 85
cell index 86
cell index 91
cell index 92
cell index 93
Spearman correlation coefficient 0.891
Spearman correlation coefficient 0.907
log normalised counts from bulk sequencing
log mean normalised counts from single cells
serum1
2i
number of reads (millions)
cell index
mean % of reads
reads mapped to exons
reads mapped, no feature
unmapped reads
serum + LIF
2i + LIF
alternative 2i + LIF
40,XY[10]
a2i
pErk1/2
Erk1/2

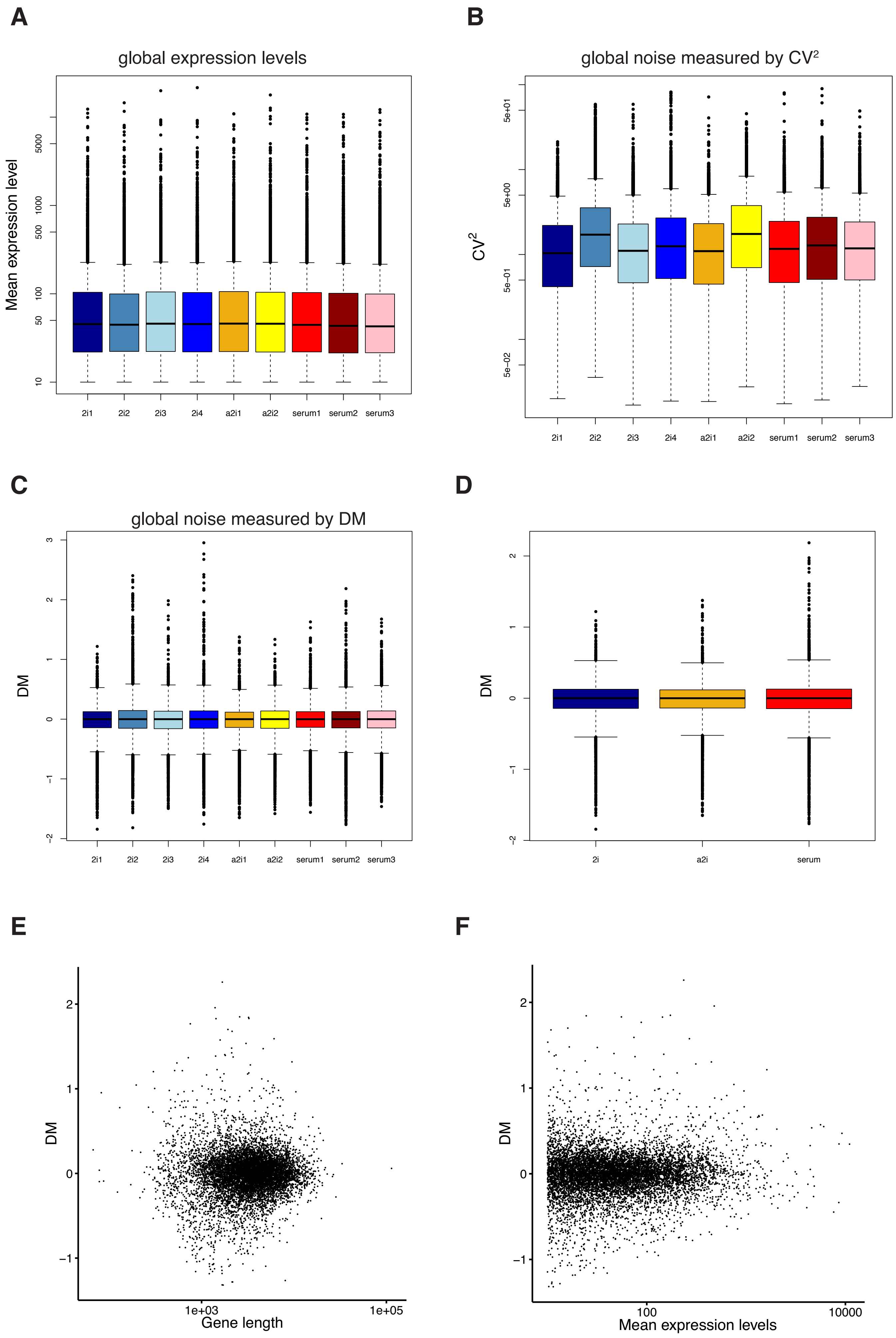

A
global expression levels
Mean expression level
10
50
100
500
1000
5000
2i1
2i2
2i3
2i4
a2i1
a2i2
serum1
serum2
serum3
B
global noise measured by CV²
CV²
5e-02
5e-01
5e+00
5e+01
2i1
2i2
2i3
2i4
a2i1
a2i2
serum1
serum2
serum3
C
global noise measured by DM
DM
-2
-1
0
1
2
3
2i1
2i2
2i3
2i4
a2i1
a2i2
serum1
serum2
serum3
D
DM
-2
-1
0
1
2
2i
a2i
serum
E
DM
-1
0
1
2
1e+03
1e+05
Gene length
F
DM
-1
0
1
2
100
10000
Mean expression levels

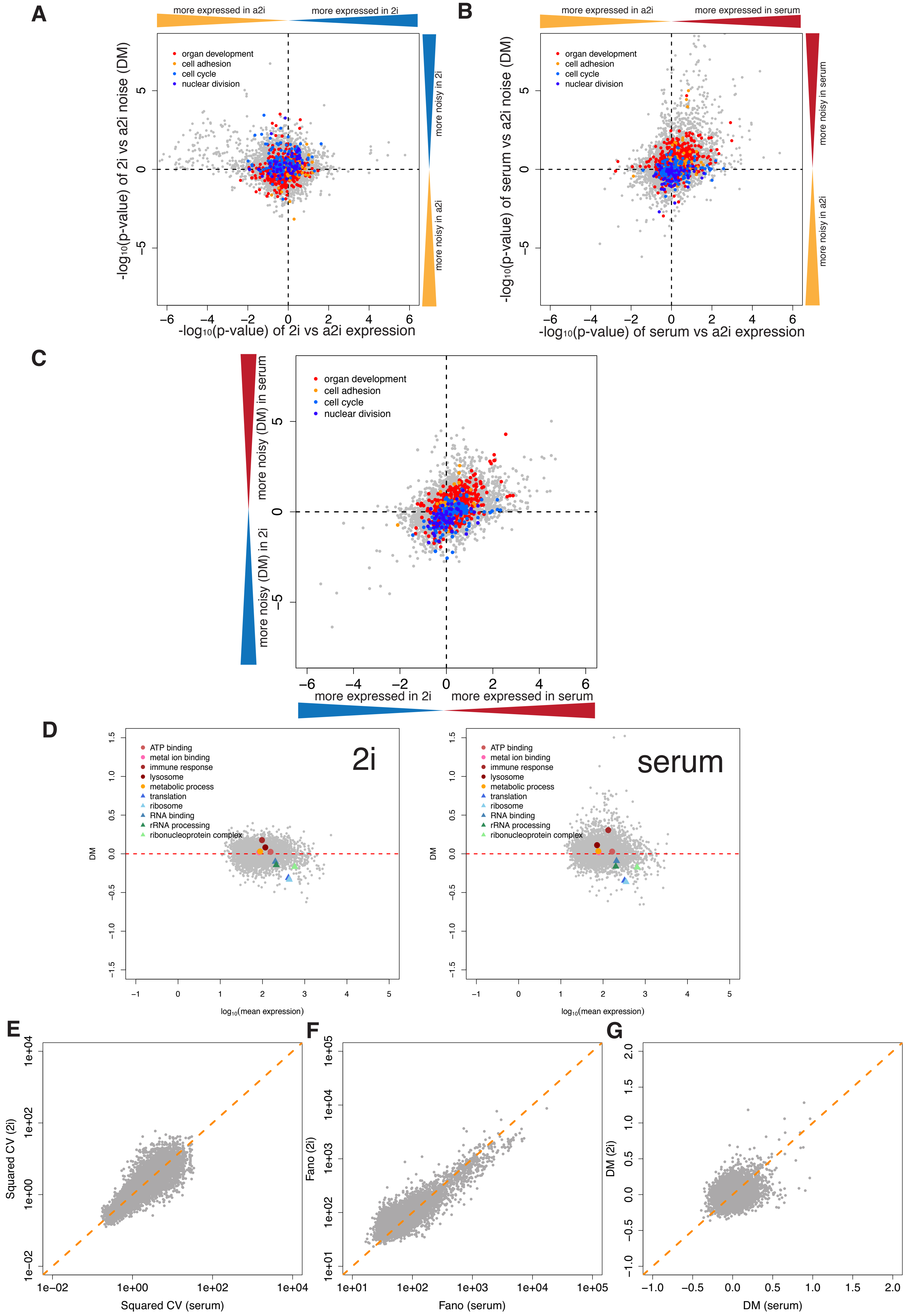
A
more expressed in a2i
more expressed in 2i
more noisy in 2i
more noisy in a2i
organ development
cell adhesion
cell cycle
nuclear division
-log10(p-value) of 2i vs a2i noise (DM)
-log10(p-value) of 2i vs a2i expression
B
more expressed in a2i
more expressed in serum
more noisy in serum
more noisy in a2i
organ development
cell adhesion
cell cycle
nuclear division
-log10(p-value) of serum vs a2i noise (DM)
-log10(p-value) of serum vs a2i expression
C
more noisy (DM) in serum
more noisy (DM) in 2i
organ development
cell adhesion
cell cycle
nuclear division
more expressed in 2i
more expressed in serum
D
2i
serum
ATP binding
metal ion binding
immune response
lysosome
metabolic process
translation
ribosome
RNA binding
rRNA processing
ribonucleoprotein complex
DM
log10(mean expression)
E
Squared CV (2i)
Squared CV (serum)
F
Fano (2i)
Fano (serum)
G
DM (2i)
DM (serum)

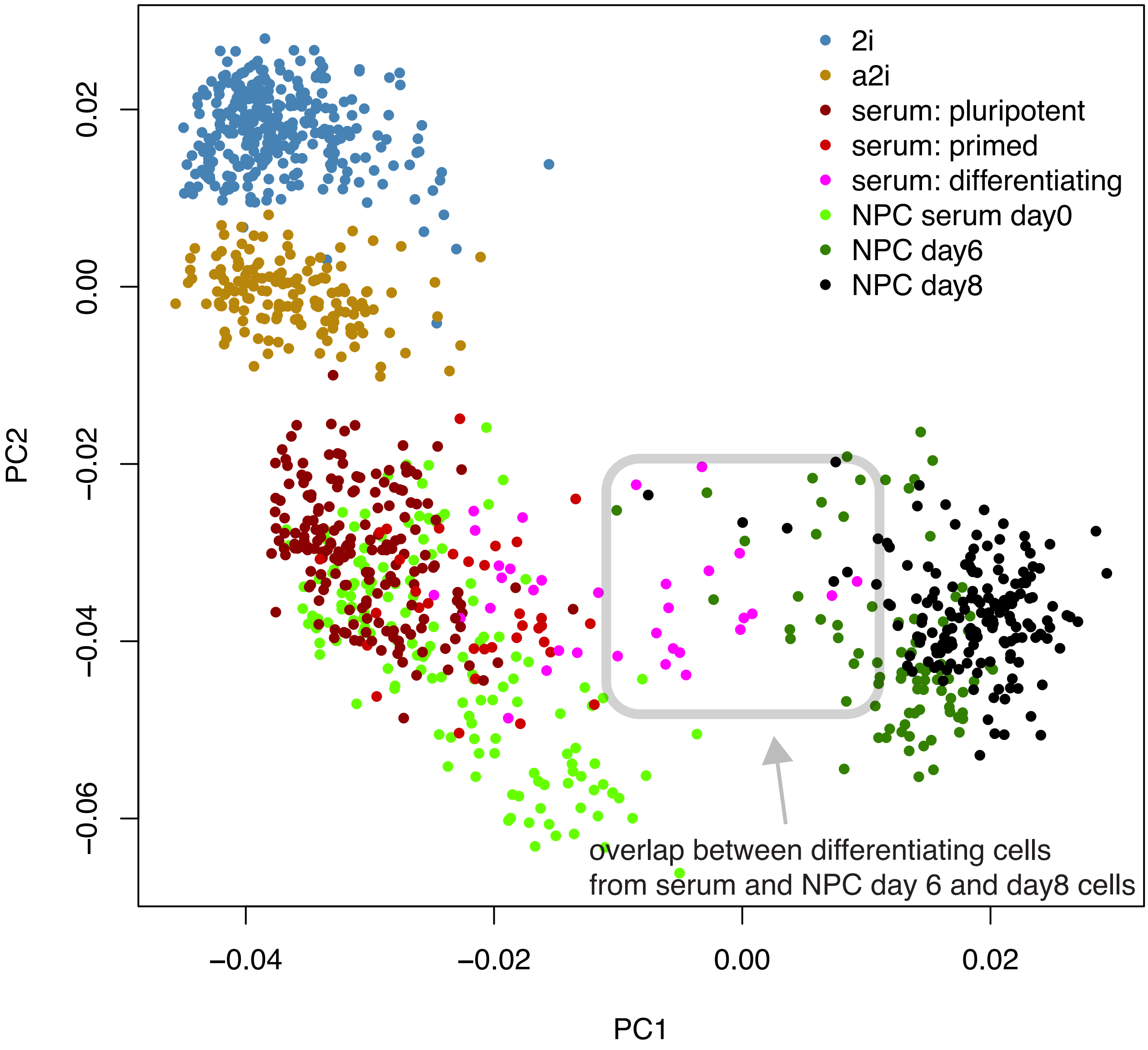

2i
a2i
serum: pluripotent
serum: primed
serum: differentiating
NPC serum day0
NPC day6
NPC day8
overlap between differentiating cells
from serum and NPC day 6 and day8 cells
PC2
0.02
0.00
−0.02
−0.04
−0.06
−0.04
−0.02
0.00
0.02
PC1

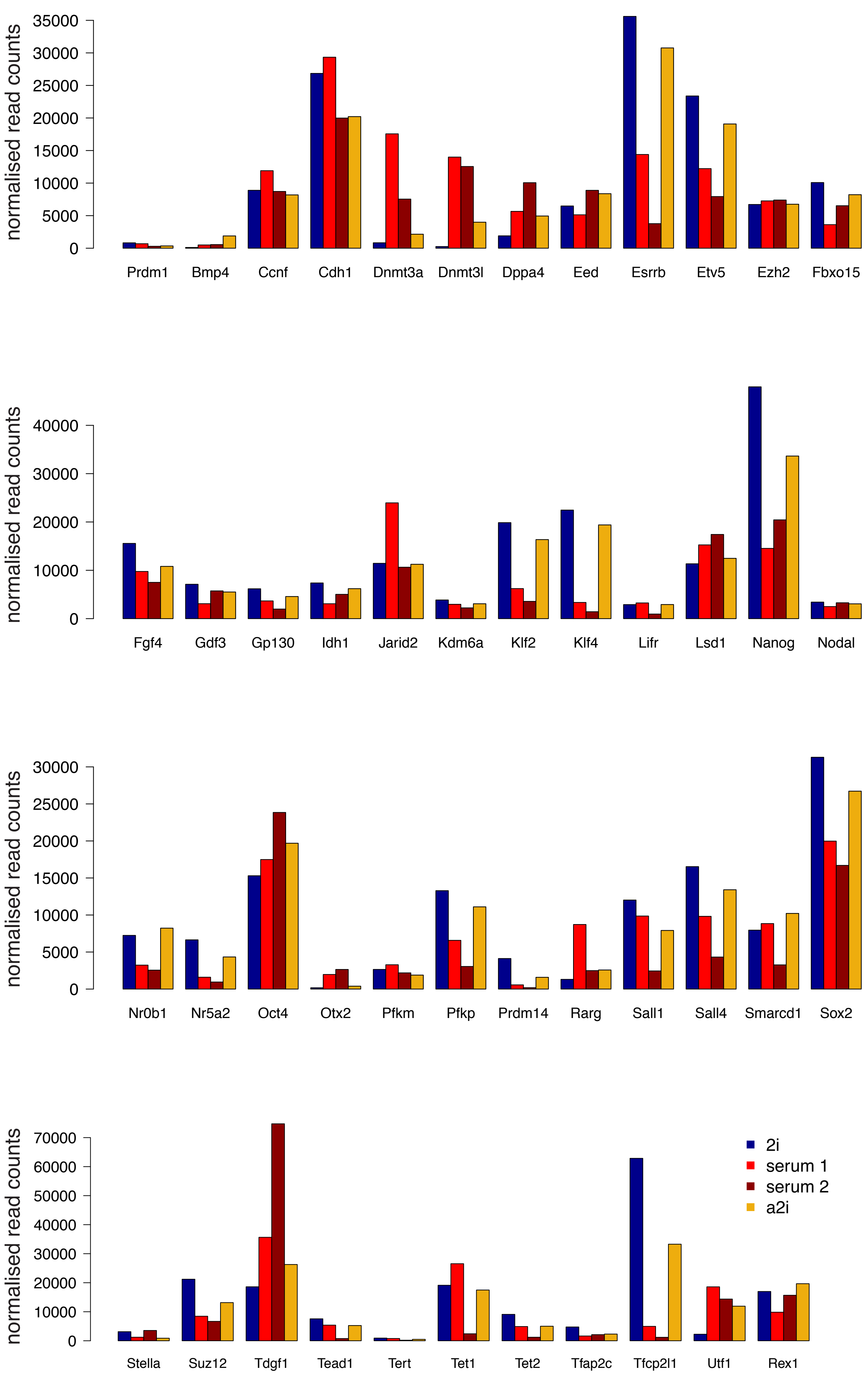

normalised read counts
35000
30000
25000
20000
15000
10000
5000
0
Prdm1
Bmp4
Ccnf
Cdh1
Dnmt3a
Dnmt3l
Dppa4
Eed
Esrrb
Etv5
Ezh2
Fbxo15
normalised read counts
40000
30000
20000
10000
0
Fgf4
Gdf3
Gp130
Idh1
Jarid2
Kdm6a
Klf2
Klf4
Lifr
Lsd1
Nanog
Nodal
normalised read counts
30000
25000
20000
15000
10000
5000
0
Nr0b1
Nr5a2
Oct4
Otx2
Pfkm
Pfkp
Prdm14
Rarg
Sall1
Sall4
Smarcd1
Sox2
normalised read counts
70000
60000
50000
40000
30000
20000
10000
0
Stella
Suz12
Tdgf1
Tead1
Tert
Tet1
Tet2
Tfap2c
Tfcp2l1
Utf1
Rex1
2i
serum 1
serum 2
a2i

A

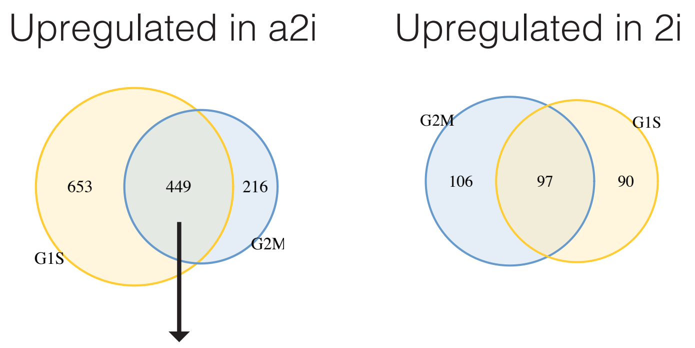

KEGG pathway	*p-val*
Ribosome	2.48e-05
Biosynthesis of amino acids	3.54e-05
Carbon metabolism	7.60e-05
Spliceosome	0.00330
RNA transport	0.00899

B

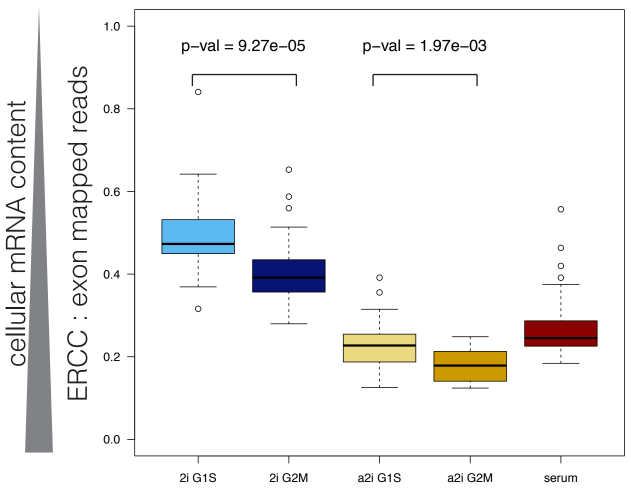

C

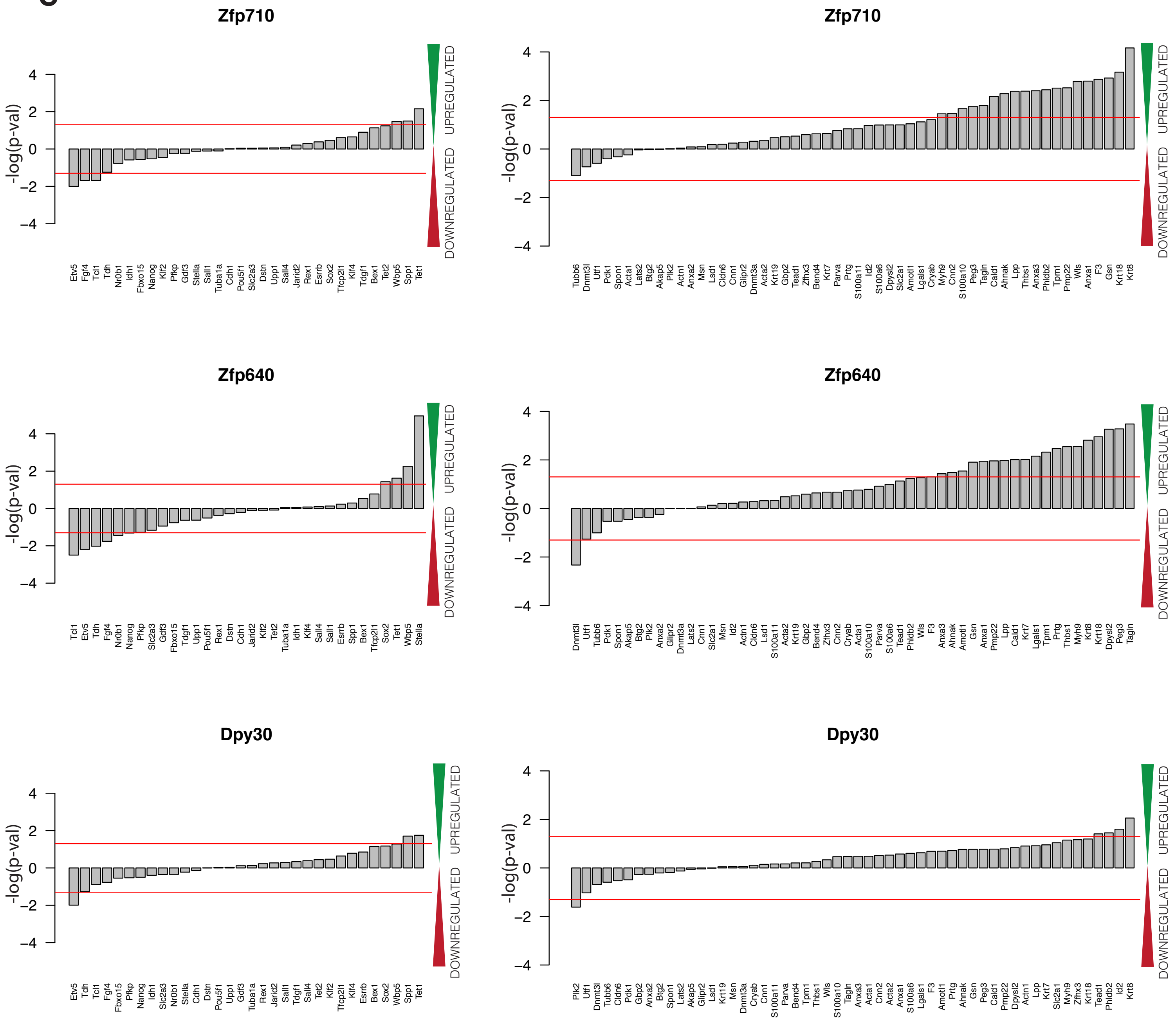

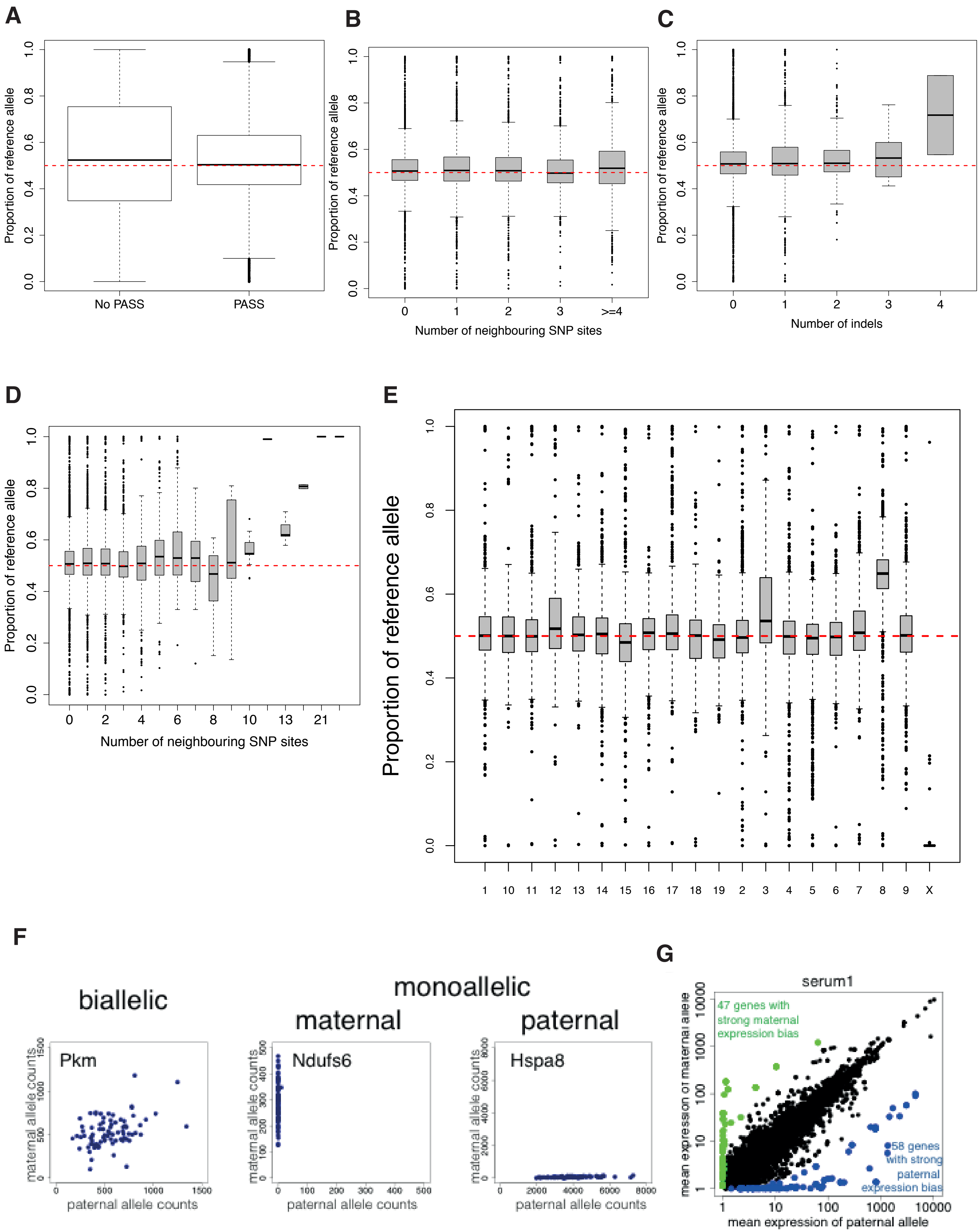

A
Proportion of reference allele
No PASS
PASS
B
Proportion of reference allele
Number of neighbouring SNP sites
C
Proportion of reference allele
Number of indels
D
Proportion of reference allele
Number of neighbouring SNP sites
E
Proportion of reference allele
F
biallelic
monoallelic
maternal
paternal
Pkm
Ndufs6
Hspa8
maternal allele counts
paternal allele counts
G
serum1
47 genes with strong maternal expression bias
58 genes with strong paternal expression bias
mean expression of maternal allele
mean expression of paternal allele

Figure S1. Quality control and mapping statistics, related to Figure 1

(A-B) Measures taken for quality control are (1) the number of reads mapping to exons being above 0.5 million, and (2) the proportion of reads mapped to mitochondrial genes being below 10%. Red dashed lines represent the thresholds we used. Red cells represent double cells, no cells or debris upon examination of the C1 chip. Cells cultured in 2i 1 are in panel A and serum 1 cells in B. (C) Correlation of gene expression levels in single cells. Expression levels correlate with each other as shown representatively for cells index 86, 91, 92 and 93 (serum 1). We removed cell 85, as it is substantially different than any other cell, suggesting failure of the experimental protocol. (D-E) Comparison of gene expression levels between bulk and single cells. Scatter plot between expression level in bulk experiment and mean of gene expression from single cells in each condition: 2i (D), serum1 (E). (F-H) Mapping statistics for each cell for each sample. GSNAP was used for mapping, and HTSeq for generating read count tables. The average percentage of reads mapping to Ensembl exons (green), to the genome outside Ensembl annotated regions (blue) and unmapped reads (red) for each of nine experiments. (I) Morphology and karyotype of G4 mES cell line. Light microscopy photograph depicting morphology of cells grown in three conditions. (J) Karyotyping result of the G4 (C57BL/6Ncr x 129S6/SvEvTac) mouse hybrid embryonic stem cells, showing no chromosomal aberrations. (K) Western blot of phosphorylation state of Erk1/2 – samples from left to right: 2i, a2i.

Figure S2. Gene expression noise measures, related to Figure 2

(A-C) Comparison of global gene expression across three culture conditions. Gene expression (A), squared coefficient of variation (B) and DM (C) distribution in all conditions. Gene expression is quantified in terms of counts normalised by reads per million. (D-E) Comparison of DM of all expressed genes among conditions processed in the same batch not including 2C-like cells. (E-F) DM (y-axis) is not correlated with gene length (E, Spearman's rho=0.0206) or mean normalized read counts (F, Spearman's rho=0.0200).

Figure S3. Gene expression noise comparison, related to Figure 2

(A-B) Comparison of gene expression noise for GO categories between 2i and a2i (A), or between serum and a2i (B). See the legend of Figure 6 for details. (C) Comparison of gene expression noise

for GO categories between 2i and a2i including 2C-like cells into 2i population (D) Specific functional categories of genes have a higher or lower level of noise in 2i1, and serum2. In the scatter plot, each point represents a GO term and some GO terms are determined to be significant (higher or lower noise than expected by chance) if a *P*-value from gene set enrichment analysis using DM is lower than 10^{-4}. See main text for details. (E-G) Noise comparison between 2i and serum data published in Grun et al., 2014. Scatterplots showing squared CV (E), Fano factor (F) and DM (G) for each gene between two conditions.

Figure S4. Differentiating mESC cells from serum culture overlap on PCA with differentiating NPC cells, related to Figure 3

mESC and NPC differentiating cells projected on the first two principal components. All genes with mean normalized counts larger than 50 were considered and PCA was performed on the Spearman's rank correlation matrix between cells.

Figure S5. Expression of key pluripotency factors in bulk mRNA sequencing data, related to Figure 5

Barplots showing size factor normalized counts for 64 genes involved in pluripotency

Figure S6. Comparison between 2i and a2i grown cells, related to Figure 5

(A) Venn diagrams showing number of differentially expressed genes between G1/S stage and G2/M stage 2i and a2i cells, KEGG pathway analysis (done using DAVID http://david.abcc.ncifcrf.gov/). (B) Comparison of mRNA content in cells using ratio of reads mapping to ERCCs (constant number of molecules spiked in in three conditions) to all exon mapped reads. (C) Barplots showing sign(log(fold change)) * –log(p-value) for differential expression of pluripotency (left) and differentiation genes. The genes that are downregulated have negative numbers, and genes that are upregulated positive. Red line indicates p-value 0.05.

Figure S7. Quality control for SNPs and, related to Figure 1

(A) SNPs not annotated as "PASS" in the FILTER column of the VCF file are in general biased toward the reference allele in the bulk data of the serum condition. (B-D) The number of

neighbouring SNP sites (B-C) and indels (D) is positively correlated with the proportion of the reference allele. (E) SNPs on chromosome 3, 8, and 12 are biased toward the reference allele. See Supplemental Methods for details. (F) Examples of genes from three gene expression categories: (1) biallelic, (2) monoallelic maternal and (3) monoallelic paternal. (G) Global overview of mean gene expression of both alleles for each gene in serum. Expression levels shown are numbers of reads mapping to the longest transcript of a gene. Genes identified as monoallelic maternal are highlighted in green and monoallelic paternal genes in blue.

Table S1. Enriched Gene Ontology categories in each condition, related to Figure 4. Results of Gene Ontology analysis of differentially expressed genes in three comparisons, serum with 2i, 2i with a2i and serum with a2i.

Table S2. Monoallelic genes, related to Figure 1.
List of genes that exhibit monoallelic expression in all culture conditions

Table S3. gRNA sequences, related to Figure 7.
Primer sequences used for cloning into gRNA vector

Extended Experimental Procedures

Bulk RNA sequencing

Bulk mRNA sequencing libraries were prepared and sequenced using the Wellcome Trust Sanger Institute sample preparation pipeline with Illumina's TruSeq RNA Sample Preparation v2 kit. RNA was extracted from 1-2 million cells using the Qiagen RNA Purification Kit on a QiaCube robot. The quality of the RNA sample was checked using gel electrophoresis. For library preparation, poly-A RNA was purified from total RNA using oligo-dT magnetic pull-down. Subsequently, mRNA was fragmented using metal-ion catalyzed hydrolysis. The cDNA was synthesized using random hexamer priming, and end repair was performed to obtain blunt ends. A-tailing was done to enable subsequent ligation of Illumina paired-end sequencing adapters, and samples were multiplexed at this stage. The resulting library was amplified using 10 cycles of PCR, substituting the Kapa Hifi polymerase for the polymerase in the Illumina TruSeq kit. Samples were diluted to 4nM, and 100bp paired end sequencing carried out on an Illumina HiSeq2000. Sequencing Quality Control was performed by the Sanger sequencing facility.

Gene-level alignment of reads

For each cell, paired-end reads were aligned to the *Mus musculus* genome (GRCm38) using GSNAP (version gmap-2014-05-15_v2) with default options (Wu and Nacu, 2010). To detect splice junctions in reads, we used a set of known splice sites from the GTF file for GRCm38 provided by Ensembl (release 73). Only reads uniquely mapped to the genome were counted for each gene using htseq-count and the same GTF file (Anders et al., 2014).

Allele-level alignment of reads

To count reads at each SNP between C57BL/6Ncr and 129S6/SvEvTac, we constructed a set of SNPs common to all the 129 strains by taking an intersection of SNPs present in the three 129 strains (129S1/SvImJ, 129S5/SvEvBrd and 129P2/OlaHsd) that are available in the Sanger mouse genomes project (Keane et al., 2011). We also confirmed that the resulting 5,038,206 SNPs do not overlap with SNPs in C57BL/6NJ. The genome of the 129 strain was constructed by computationally mutating the SNP positions between 129 and C57BL/6J. The paired-end reads were mapped to both the reference and mutated genomes using GSNAP (version gmap-2014-05-15_v2) with default options (Wu and Nacu, 2010). Reads mapped to multiple locations were excluded. We extracted 120,036 exonic SNPs using the GTF file for GRCm38 provided by Ensembl (release 73) and found that a total of 31,695 transcripts have at least one exonic SNP. We counted reads for each exonic SNP using mpileup of samtools (version 0.1.19) (Li et al., 2009).

Quality control on SNPs

The 120,036 exonic SNPs were further examined to avoid bias in measures of allele-specific expression. First, we found that SNPs not annotated as "PASS" in the FILTER column of the VCF file provided by the Mouse Genomes Project (version 3) were biased toward the reference allele in the bulk data of serum (hereafter we will use the same data to test the quality of SNPs), suggesting SNPs that do not pass all filter criteria for a genotype call are unreliable. Second, the extent of unequal allelic abundance increased with the number of neighbouring SNP sites (i.e. the number of other SNP sites located within the SNP-flanking region, ± read length), which is consistent with a previous study showing the impact of the density of neighbouring SNP sites on the allelic abundance (Stevenson et al., 2013). Third,

increasing the number of insertions or deletions (indels) between 129 and C57BL/6J within the SNP-flanking region impaired the measures of allelic abundance, suggesting that the presence of indels can prevent the alignment of reads from 129 alleles to the mutated genome. Finally, SNPs on chromosomes 3, 8, and 12 were biased toward the reference allele. To exclude SNPs affected by the systematic bias toward the reference allele, we used the following criteria:

1) SNP quality annotation: only SNPs annotated as "PASS" were considered.

2) Density of neighbouring SNP sites: SNPs with the number of neighbouring SNP sites larger than or equal to 4 were removed.

3) Indels: SNPs with at least one indel nearby were removed.

4) Chromosome: SNPs on chromosome 3, 8, 12, and X (since the mouse is male) were removed.

In total, we have 66,291 exonic SNPs and 25,104 transcripts with at least one such SNP. As a quality check for the final exonic SNPs, we investigated the proportion of reads mapped to the maternal alleles of the X chromosome and found that 97.6-98.9% of reads mapped to SNPs on the X chromosome (i.e. that passed the first three criteria above) were correctly assigned to the maternal allele (129S6/SvEvTac) in the bulk data of the three culture conditions.

Identification of monoallelic genes

To identify genes with monoallelic expression, we computed the following three quantities for each transcript with at least one exonic SNP: 1) Proportion of paternal allele in the bulk

(αb), 2) P-value of binomial exact test in the bulk (P), where the null hypothesis is that the two alleles are equally expressed in the bulk, 3) Average proportion of paternal allele in the single cells (αs). If a transcript has multiple SNPs, we summed the read counts of these SNPs. A transcript is called monoallelic if ($\alpha b<0.05$ or $\alpha b>0.95$) and $P\leq0.01$ and ($\alpha s<0.05$ or $\alpha s>0.95$).

Expression of maternal *versus* paternal alleles defines monoallelic expression patterns.

In all analyses so far, we consider expression of a gene as the sum of expression from the maternal and paternal alleles. However, the SNPs between the two parental strains of our hybrid mESCs allow us to map reads to either the maternal or paternal allele for many genes, and quantify allele specific expression (Methods, Figure S7). To avoid potential confounding factors we karyotyped the cells and observed no abnormalities (Figure S3B). There are 13,642 genes with at least one SNP. After filtering lowly expressed genes and genes with uncertain SNPs, we were left with an average of 4,201 genes per condition for which we can distinguish maternal and paternal expression.

The previous literature on allele-specific expression of genes in mESCs contains various results, some of which appear to be contradictory. RNA-FISH for *Nanog* in 2i medium showed that *Nanog* expression is biallelic in most cells, while its expression in serum was reported to be lower and mostly monoallelic. Interestingly, other genes that have similar expression profiles to *Nanog* in our experiments, such as *Rex1*, were observed to be biallelically expressed according to RNA-FISH (Miyanari and Torres-Padilla, 2012). At the protein level, reporter constructs suggest that both alleles of *Nanog* are expressed. It has been pointed out that burst-like transcription rather than the traditional concept of monoallelic expression may be the origin of expression of one allele per cell (Faddah et al., 2013; Filipczyk et al., 2013).

Monoallelic expression is defined as all cells expressing only one allele. We found 155 monoallelically-expressed genes (91 paternal and 63 maternal)(Full list in Table S2). This corresponds to only 2% of the genes in our data set (Figure 7). 53 genes are consistently monoallelically expressed in all conditions. Monoallelic expression can be due to imprinting, or it can be a random process, mediated by a different, unknown mechanism, including burst-like kinetics of transcription.

We do not observe big differences in the number of monoallelic genes identified between conditions. There are 96 monoallelic genes in 2i1, 105 in serum1, 93 in serum2 and 81 in a2i1. We found only one instance when a transcript was identified as monoallelic in one condition, and biallelic in another. Some genes that are monoallelically expressed in one condition have low expression level in the other and do not pass through our expression level filter, so are not classified as either monoallelic or biallelic. Based on these results, monoallelic expression seems to be independent of the culture condition.

Quality control of cells

To exclude poor quality libraries from downstream analysis, we first removed cells that correspond to empty capture sites, capture sites with multiple cells, or capture sites containing cell debris on the C1 chip by visually inspecting them under microscope. Second, it has been known that some cells suffer from cell rupture during the process of microfluidic cell capture (Islam et al., 2014). To identify these abnormal cells, we calculated two quantities for each cell: the number of reads mapped to exons, and the proportion of reads (of all reads mapped to exons) mapped to 37 genes on the mitochondrial chromosome. We identified two populations of cells in serum1 in terms of the above two quantities and most of

the cells corresponding to empty cells or cell debris are in one of the two populations. Based on this, we set the following criteria to remove abnormal cells:

1) Cells that have fewer than 500,000 reads mapped to exons

2) Cells that have greater than 10% reads mapped to mitochondrial genes

We also validated the above criteria using the cells in 2i1, and found that 7 of 9 cells (empty or cell debris identified by visual inspection) were not accepted (Figure S1AB). Finally, we compared the normalised read counts of genes between cells in the same condition, and found that in one cell (cell "85" in the first replicate of serum) there was a problem in library preparation and many genes were abnormally amplified (Figure S1C). We removed the cell from further analysis. In summary, we have the following number of cells for the analysis: 81, 90 and 79 for serum replicates, 82, 59,72 and 82 for 2i replicates, 93 and 66 for a2i replicates, where the total number of cells across conditions is 704.

Adjusting for batch effects

To remove technical variation across multiple batches, we applied location and scale adjustments to the normalized read counts by using the ComBat function of the sva package of R with default options (Johnson et al., 2007). We first log10-transformed the normalized read counts (after removing lowly expressed genes whose mean normalised read counts are less than 10) and after adding a pseudo count of 1. Second, we adjusted for batch effects using ComBat with the known batch covariate and sample conditions. Finally, we re-transformed the batch-adjusted expression values x back to the original scale (10^x-1). If the re-transformed values are less than 0 or the original read counts are 0, we set the re-transformed values to 0.

Differential expression analysis

Differentially expressed genes were identified from bulk data and single cell data using the DESeq package. Genes that differed in expression by two fold and with a multiple testing adjusted p-value was < 0.05 were considered differentially expressed. For single cell differential expression analysis each cell was used as a replicate of the condition it came from and genes that had mean expression below 50 (DESeq size factor normalised) counts were removed.

Calculating DM as a measure of noise

To account for the confounding effects of gene length and mean expression level on the CV, we computed the DM values for each gene using rolling medians of the squared CV. First, we computed gene lengths by taking the union of all exons within a gene based on the Ensembl annotation. Here we excluded all exons annotated as "retained_intron". We also removed lowly expressed genes whose mean normalised read counts (reads per million) are less than 10, since we cannot distinguish biological noise from technical noise for these genes. Second, we computed rolling medians from the scatter plot between the mean normalised read counts and the squared CV values, where the x- and y-axis are log10 transformed. Third, we calculated the mean-corrected residual of the squared CV of gene i to its corresponding rolling median $f(i)$ such that

$$r(i) = \log_{10} CV(i)^2 - f(i).$$

Finally, to correct for the effect of gene length on the mean corrected residual, we calculated the difference between the mean corrected residual of the squared CV of gene i and its expected residual by using the following formula

$$DM(i) = r(i) - g(i),$$

where $g(i)$ is the rolling median of gene i from the scatter plot between $r(i)$ and log10 transformed gene lengths. To compute the rolling medians, we used the rollapply function of the zoo package of R (Zeileis and Grothendieck, 2005) and the following parameters: the number of genes in the window is 50 and the number of overlapping genes between adjacent windows is 25. This relative noise measure, which is referred to as DM, does not depend on either gene expression levels or gene lengths (Spearman's ρ=0.0200 for gene expression levels and ρ=0.0206 for gene length in the serum condition).

Analyzing the published single cell RNA-seq data of Grun *et al.* 2014

We performed noise analysis using data published previously (Grun et al., 2014) and validated our observation that there is no significant difference in noise levels between cells grown in 2i and serum. We observed that development and differentiation related GO categories were noisier in serum, while cell cycle related ones were noisier in 2i.

The expression levels of 10,809 genes that are expressed in both conditions are significantly higher in 2i than in the serum condition ($P<2.2\times10^{-16}$ by the Wilcoxon signed-rank test), while the estimated biological variance differs significantly in the same direction ($P<2.2\times10^{-16}$). This leads to a significant difference in the distribution of CV values between conditions ($P<2.2\times10^{-16}$, higher in serum, Figure S8A) but to a significant difference in the distribution of Fano factors in the opposite direction ($P<2.2\times10^{-16}$, higher in 2i, Figure S8B). To properly account for the confounding effect of expression level on noise measures, we used the abundance-corrected noise measure (DM). We observed no

significant difference of DM values between the two culture conditions (P=0.8740, Figure S8C). Since we cannot conclude that the expression of genes across the whole dynamic range of expression levels is more heterogeneous in the serum condition than in 2i, we asked whether noise levels in specific functional categories differ between the two culture conditions. We found that 92 Gene Ontology terms (out of 17,849) show a significant difference in their DM values (P<0.01 by the paired t-test). Notably, genes that play a role in mitosis have higher levels of noise in 2i compared to serum. In contrast, genes involved in development are more heterogeneous in serum than in 2i.

Testing the absolute level of cell-to-cell variation of a functional category within a culture condition

To test whether genes belonging to a defined functional category have a high or low level of expression heterogeneity within a culture condition, we performed gene set enrichment analysis using the Piano package of Bioconductor (Varemo et al., 2013). We used the DM values for gene-level statistics and calculated the mean DM values as a gene-set statistic for each GO term. The associations between Ensembl gene IDs and GO terms were obtained from the biomaRt package of Bioconductor (Kasprzyk, 2011). Since gene set enrichment analysis tends to bias towards large or small categories in terms of their number of genes, we considered only gene sets with between 3 and 2,000 genes. The *P*-value for each GO term was then computed by randomly taking a set of genes of the same size as in the GO term, and by repeating this 10,000 times.

Testing the relative difference in expression heterogeneity of a functional category across culture conditions

To explore further the difference of the three culture conditions in terms of gene expression noise, we compared two sets of DM values for each GO term between two culture conditions using the two-sided paired t-test. We only considered GO terms with at least 2 genes having DM values. The associations between GO terms and their offspring terms were obtained from the GO.db annotation package of Bioconductor

(http://www.bioconductor.org/packages/release/data/annotation/html/GO.db.html).

Doubling time estimation of mouse embryonic stem cells in different conditions

Fifty thousands G4 mouse ES cells were plated in single wells on gelatinized 6-well plates, and maintained in the three culture conditions of interest (total 12 wells for each culture condition): serum-containing media, standard 2i media and alternative 2i media. Three wells were harvested and quantified on a hemocytometer every 24 hours for 4 days to estimate the doubling time of mouse ES cells in each condition.

Western Blotting

Cells were cultured in standard 2i media: N2B27 basal media (NDiff 227, StemCells), 1 μM PD0325901 (Stemgent), 3 μM CHIR99021 (Stemgent) and alternative 2i media: N2B27 basal media (NDiff 227, StemCells), 1 μM CGP77675 (Sigma), 3 μM CHIR99021 (Stemgent). Samples were prepared from 5mln of cells, lysed, and diluted in to final volume of 200ul 1x Laemmli buffer. 10ul of each sample was run on 10% Mini-PROTEAN® TGX™ Precast Protein Gel and blotted to nitrocellulose membrane. The efficiency of blotting was inspected using Ponceau stain. Blots were blocked with 5%BSA in TBS and washed with 0.05% Tween20 TBS. Primary antibodies used were p44/42 MAPK (Erk1/2) #9102 and Phospho-p44/42 MAPK (Erk1/2) (Thr202/Tyr204) #9101 from Cell Signalling. HRP conjugated

secondary antibodies were used and Thermo Scientific Pierce ECL Western Blotting Substrates were used for detection.

References

Anders, S., Pyl, P.T., and Huber, W. (2014). HTSeq – A Python framework to work with high-throughput sequencing data. bioRxiv preprint.
Faddah, D.A., Wang, H., Cheng, A.W., Katz, Y., Buganim, Y., and Jaenisch, R. (2013). Single-cell analysis reveals that expression of nanog is biallelic and equally variable as that of other pluripotency factors in mouse ESCs. Cell stem cell *13*, 23-29.
Filipczyk, A., Gkatzis, K., Fu, J., Hoppe, P.S., Lickert, H., Anastassiadis, K., and Schroeder, T. (2013). Biallelic expression of nanog protein in mouse embryonic stem cells. Cell stem cell *13*, 12-13.
Grun, D., Kester, L., and van Oudenaarden, A. (2014). Validation of noise models for single-cell transcriptomics. Nat Methods *11*, 637-640.
Islam, S., Zeisel, A., Joost, S., La Manno, G., Zajac, P., Kasper, M., Lönnerberg, P., and Linnarsson, S. (2014). Quantitative single-cell rna-seq with unique molecular identifiers. Nat Methods *11*, 163.
Johnson, W.E., Li, C., and Rabinovic, A. (2007). Adjusting batch effects in microarray expression data using empirical Bayes methods. Biostatistics *8*, 118-127.
Kasprzyk, A. (2011). BioMart: driving a paradigm change in biological data management. Database *2011*.
Keane, T.M., Goodstadt, L., Danecek, P., White, M.A., Wong, K., Yalcin, B., Heger, A., Agam, A., Slater, G., Goodson, M., *et al.* (2011). Mouse genomic variation and its effect on phenotypes and gene regulation. Nature *477*, 289-294.
Li, H., Handsaker, B., Wysoker, A., Fennell, T., Ruan, J., Homer, N., Marth, G., Abecasis, G., Durbin, R., and Genome Project Data Processing, S. (2009). The Sequence Alignment/Map format and SAMtools. Bioinformatics *25*, 2078-2079.
Miyanari, Y., and Torres-Padilla, M.E. (2012). Control of ground-state pluripotency by allelic regulation of Nanog. Nature *483*, 470-473.
Stevenson, K.R., Coolon, J.D., and Patricia J Wittkopp, P.J. (2013). Sources of bias in measures of allele-specific expression derived from RNA-seq data aligned to a single reference genome. BMC genomics *14*.
Varemo, L., Nielsen, J., and Nookaew, I. (2013). Enriching the gene set analysis of genome-wide data by incorporating directionality of gene expression and combining statistical hypotheses and methods. Nucleic acids research *41*, 4378-4391.
Wu, T.D., and Nacu, S. (2010). Fast and SNP-tolerant detection of complex variants and splicing in short reads. Bioinformatics *26*, 873-881.
Zeileis, A., and Grothendieck, G. (2005). zoo: S3 Infrastructure for Regular and Irregular Time Series. J Stat Soft *14*.
